# Measurement Properties of the Patient Health Questionnaire–15 and Somatic Symptom Scale–8: A Systematic Review and Meta-Analysis

**DOI:** 10.1001/jamanetworkopen.2024.46603

**Published:** 2024-11-20

**Authors:** Jonna Hybelius, Amanda Kosic, Sigrid Salomonsson, Caroline Wachtler, John Wallert, Steven Nordin, Erland Axelsson

**Affiliations:** 1 Division of Family Medicine and Primary Care, Department of Neurobiology, Care Sciences and Society, Karolinska Institutet, Huddinge, Sweden; 2 Liljeholmen University Primary Health Care Centre, Academic Primary Health Care Centre, Region Stockholm, Stockholm, Sweden; 3 School of Law, Psychology and Social Work, Örebro University, Örebro, Sweden; 4 Centre for Psychiatry Research, Department of Clinical Neuroscience, Karolinska Institutet, Stockholm, Sweden; 5 Department of Psychology, Umeå University, Umeå, Sweden

## Abstract

**Importance:**

The subjective experience of somatic symptoms is a key concern throughout the health care system. Valid and clinically useful instruments are needed.

**Objective:**

To evaluate the measurement properties of 2 widespread patient-reported outcomes: the Patient Health Questionnaire–15 (PHQ-15) and Somatic Symptom Scale–8 (SSS-8).

**Data Sources:**

Medline, PsycINFO, and Web of Science were last searched February 1, 2024.

**Study Selection:**

English-language studies reporting estimates pertaining to factor analysis, taxometric analysis, internal consistency, construct validity, mean scores in relevant groups, cutoffs, areas under the receiver operating characteristic curves (AUROCs), minimal clinically important difference, test-retest reliability, or sensitivity to change.

**Data Extraction and Synthesis:**

Search hits were reviewed by independent raters. Cronbach α, Pearson *r*, means, and between-group effect sizes indicative of sensitivity to change were pooled in random-effects meta-analysis. Study quality was assessed using 3 instruments. Reporting followed the Preferred Reporting Items for Systematic Reviews and Meta-Analyses 2020 reporting guideline.

**Main Outcomes and Measures:**

Comprehensive overview of evidence pertaining to the measurement properties of the PHQ-15 and SSS-8.

**Results:**

A total of 305 studies with 361 243 participants were included. Most concerned routine care (178 studies) and the general population (27 studies). In factor analyses, both scales reflected a combination of domain-specific factors (cardiopulmonary, fatigue, gastrointestinal, pain) and a general symptom burden factor. The pooled PHQ-15 α was 0.81 (95% CI, 0.80-0.82), but with low item-total correlations for items concerning menstrual problems, fainting spells, and sexual problems (item-total correlations <0.40), and the SSS-8 α was 0.80 (0.77-0.83). Pooled correlations with other measures of somatic symptom burden were 0.71 (95% CI, 0.64-0.78) for the PHQ-15 and 0.82 (95% CI, 0.72-0.92) for the SSS-8. Reported AUROCs for identification of somatoform disorders ranged from 0.63 (95% CI, 0.50-0.76) to 0.79 (95% CI, 0.73-0.85) for the PHQ-15 and from 0.71 (95% CI, 0.66-0.77) to 0.73 (95% CI, 0.69-0.76) for the SSS-8. The minimal clinically important difference on both scales was 3 points. Test-retest reliability could not be pooled and was inconsistent for the PHQ-15 (PHQ-15: *r* = 0.65-0.93; ICC, 0.87; SSS-8: *r* = 0.996, ICC = 0.89). The PHQ-15 showed tentative sensitivity to change (*g* = 0.32; 95% CI, 0.08-0.56), but data for the SSS-8 were lacking.

**Conclusions and Relevance:**

In this systematic review and meta-analysis, findings supported use of the PHQ-15 and SSS-8 for the assessment of symptom burden, but users should be aware of the complex, multifactorial structures of these scales. More evidence is needed concerning longitudinal measurement properties.

## Key Points

**Question:**

What is known about the measurement properties of the Patient Health Questionnaire–15 (PHQ-15) and Somatic Symptom Scale–8 (SSS-8)?

**Findings:**

This systematic review and meta-analysis of 305 studies with 361 243 participants found that general and symptom domain–specific factors contributed to response patterns. The PHQ-15 (α = 0.81) and SSS-8 (α = 0.80) exhibited adequate internal consistency, but with redundant PHQ-15 items. Correlations with other scales generally supported construct validity; a difference of 3 or greater constituted a relevant change on both scales; and screening properties for the identification of somatoform disorders were suboptimal.

**Meaning:**

The findings of this study suggest that the PHQ-15 and SSS-8 can be recommended for assessment and monitoring of somatic symptom burden, but clinicians need to be aware that such scores reflect complex, multifactorial structures.

## Teaser

This systematic review and meta-analysis examines the measurement properties of 2 commonly used patient-reported outcome scales: the Patient Health Questionnaire–15 (PHQ-15) and the Somatic Symptom Scale–8 (SSS-8).

## Introduction

The subjective experience of somatic symptoms is a core aspect of many medical conditions. This is a complex and multifactorial phenomenon, influenced by biological, psychological, and social aspects,^1,2,3^ and associated with substantial distress, functional impairment, and societal costs.^4,5^ In the general medical setting, persistent physical symptoms that last for months or more are among the major reasons for consultations.^6^ This problem is seen across a wide spectrum of conditions, including in cancer survivors, patients with cardiovascular disease and inflammatory bowel disease, and among individuals with conditions with a less clear etiology.^1,7,8,9,10,11^ An elevated burden of somatic symptoms is also common in psychiatric disorders, such as depression and anxiety disorders.^12,13^ For the clinician, managing prevalent persistent symptoms, such as fatigue or pain, can be demanding,^14,15^ and patients commonly remain bothered by their symptoms even after receiving standard medical treatment.

Time-efficient, noninvasive measurement strategies are needed to aid clinicians in the assessment and monitoring of somatic symptom burden. Considering that many patients are bothered by multiple symptoms (eg, pain, fatigue, and gastrointestinal complaints^6^) at the same time, a desirable feature of such an instrument is that it captures a broad set of common physical symptoms, thereby prompting further action and facilitating personalized care.^16^ To be useful in the hectic routine care environment,^17^ instruments should also be brief and easy to administer.

The Patient Health Questionnaire–15 (PHQ-15) and its abbreviated version, the Somatic Symptom Scale–8 (SSS-8), are widespread patient-reported measures of somatic symptom burden. Originally derived from the Primary Care Evaluation of Mental Disorders (PRIME-MD) interview,^18,19^ the PHQ-15 evaluates the severity of somatic symptoms experienced during the past 4 weeks. Each of the 15 items quantifies a common physical symptom, such as back pain, shortness of breath, or constipation, and is scored from 0 (not bothered at all) to 2 (bothered a lot), resulting in a sum score ranging from 0 to 30.^20^ The SSS-8 comprises 8 items, each scored from 0 (not at all) to 4 (very much), resulting in a sum score ranging from 0 to 32 and reflecting somatic symptom burden over the preceding 7 days.^21^ When the SSS-8 was developed, 3 items originally included in PHQ-15 were removed (menstrual cramps, sexual problems, and fainting spells) due to low prevalence and limited variance shared with the other items. Additionally, 5 items relating to cardiopulmonary and gastrointestinal symptoms were reduced to 2.

The PHQ-15 and SSS-8 have gained popularity in the general medical setting, and the PHQ-15 is recommended in the *Diagnostic and Statistical Manual of Mental Disorders* (Fifth Edition) (*DSM-5*), for the assessment of somatic symptoms across psychiatric disorders.^22^ Empirical reviews have recommended the PHQ-15 for use in clinical trials,^23^ large-scale studies,^24^ and the primary health care setting.^25,26^ However, existing reviews evaluating the measurement properties of these scales have been limited in scope, and the emerging literature pertaining to the SSS-8 has never been compiled in a systematic manner.

We conducted a comprehensive systematic review and meta-analysis of the measurement properties of the PHQ-15 and SSS-8. This included the latent structure of subjective somatic symptom burden and clinically oriented outcomes, such as the suitability for identifying somatoform disorders and studying symptom change. In this work, the term *somatoform disorders* is used as an umbrella term for psychiatric disorders characterized by elevated somatic symptom burden (eg, somatic symptom disorder, somatization disorder), regardless of whether the etiology of the physical symptoms are fully understood. Thus, this wider category includes both the somatoform disorders of the *Diagnostic and Statistical Manual of Mental Disorders* (Fourth Edition) and the somatic symptom and related disorders of the *DSM-5*. Preregistered specific targets and hypotheses are listed in eAppendix 1 in Supplement 1.

## Methods

This systematic review and meta-analysis was conducted at Karolinska Institutet and Liljeholmen University Primary Health Care Centre, Stockholm, Sweden. Reporting followed the Preferred Reporting Items for Systematic Reviews and Meta-Analyses (PRISMA) 2020 reporting guideline.^27^ This study was preregistered in PROSPERO (CRD42022342827) and also at the Open Science Framework.^28^

### Search Strategy and Eligibility Criteria

The final search was conducted on February 1, 2024. We searched Medline, PsycINFO, and Web of Science, using index and free-text terms. Terms for the scales of interest (eg, *PHQ*, *SSS-8*) were combined with terms for measurement evaluation (eg, psychometric*, valid*). We also searched for records citing the primary publications for the PHQ-15^20^ and SSS-8.^21^ No limitations were applied for the search to maximize sensitivity. A separate search was added for clinical trials, relevant for the study of sensitivity to change. This search combined terms for randomized clinical trials (RCTs) with terms for distress related to persistent physical symptoms (eg, *somatoform*), and was restricted to English publications from the year 2000 and later. Reference lists of previous reviews of more limited scope^24,25,29,30^ were reviewed by J. H. For full search terms, see eAppendix 2 in Supplement 1.

From the main search, we included studies published as peer-reviewed journal articles, written in English, that primarily included adults. Studies had to report at least 1 of the following: factor analysis, taxometric analysis, internal consistency, correlations relevant for construct validity, mean scores from general population or clinical samples, receiver operating characteristic curves, minimal clinically important difference (MCID), test-retest reliability, or sensitivity to change. Based on the separate clinical trials search, RCTs were included that evaluated either antidepressants or cognitive behavioral therapy (CBT; found to be effective therapies based on current evidence^1,31^) vs a rudimentary control condition (waiting list or treatment-as-usual; where a true between-group effect would be highly likely) in a population for whom somatic symptom burden is central (eg, somatic symptom disorder or functional somatic syndrome).

Unique search hits were systematically identified using Endnote version 21,^32^ and imported into Rayyan.^33^ Titles and abstracts were assessed for eligibility by 2 independent reviewers (2 of J.H., A.K., and E.A.). Articles not unanimously excluded were reviewed in full text (2 of J.H., A.K., S.S., C.W., J.W., S.N., and E.A.). Disagreements were resolved through discussion, or, if not possible, through voting.

### Data Extraction and Study Risk of Bias

Extraction of study characteristics and outcomes was conducted by J.H. or E.A. A random subset of 20 studies was assessed by both to evaluate consistency. Authors were contacted to ensure that data from the same study were not tabulated multiple times and for efficacy estimates relevant for sensitivity to change. We assessed study risk of bias based on 3 structured instruments: First, an adapted version of the instrument by Reilly and colleagues,^34^ focusing on investigator bias (high risk if the authors were developers of the PHQ-15 or SSS-8), sampling method (high risk if sample not representative for its setting), sample size, and time delay (applicable for studies reporting correlations with other scales; high risk if scales not administered the same day). Second, studies evaluating cutoffs were also assessed using the revised Quality Assessment of Diagnostic Accuracy Studies (QUADAS-2).^35^ Third, studies reporting efficacy outcomes relevant to the assessment of sensitivity to change were also assessed using the Cochrane Risk of Bias tool version 2 (RoB 2).^36^ eTable 2 in Supplement 1 provides further details on the coding schema for the assessment of study risk of bias, and eTable 3 in Supplement 1 presents the coding scheme for medical conditions, used for the pooling of means and Cronbach α.

### Statistical Analysis

All statistical analyses were performed in R version 4.3.1 (R Project for Statistical Computing), with the metafor package version 4.4-0.^37^ We pooled Pearson *r* indicative of construct validity, Cronbach α, mean scores, and CBT efficacy estimates in random-effects meta-analyses whenever the number of studies per stratum was at least 3 and summarized the remaining outcomes narratively. Hedges *g* standardized effect sizes were calculated as the posttreatment mean difference, divided by the pooled SD. Statistical heterogeneity was quantified in terms of the *Q*, τ^2^, and *I*^2^ statistics. Possible publication bias was assessed by visual inspection of funnel plots, the Duval and Tweedie trim-and-fill method,^38^ and the Egger test.^39^ This study used a 95% confidence level. Details about the statistical analysis, including methods applied for transformation and weighting of original data, general principles for result interpretation, and sensitivity analyses appear in eTable 1 and eAppendix 3 in Supplement 1.

## Results

### Study Selection

**Figure 1.**
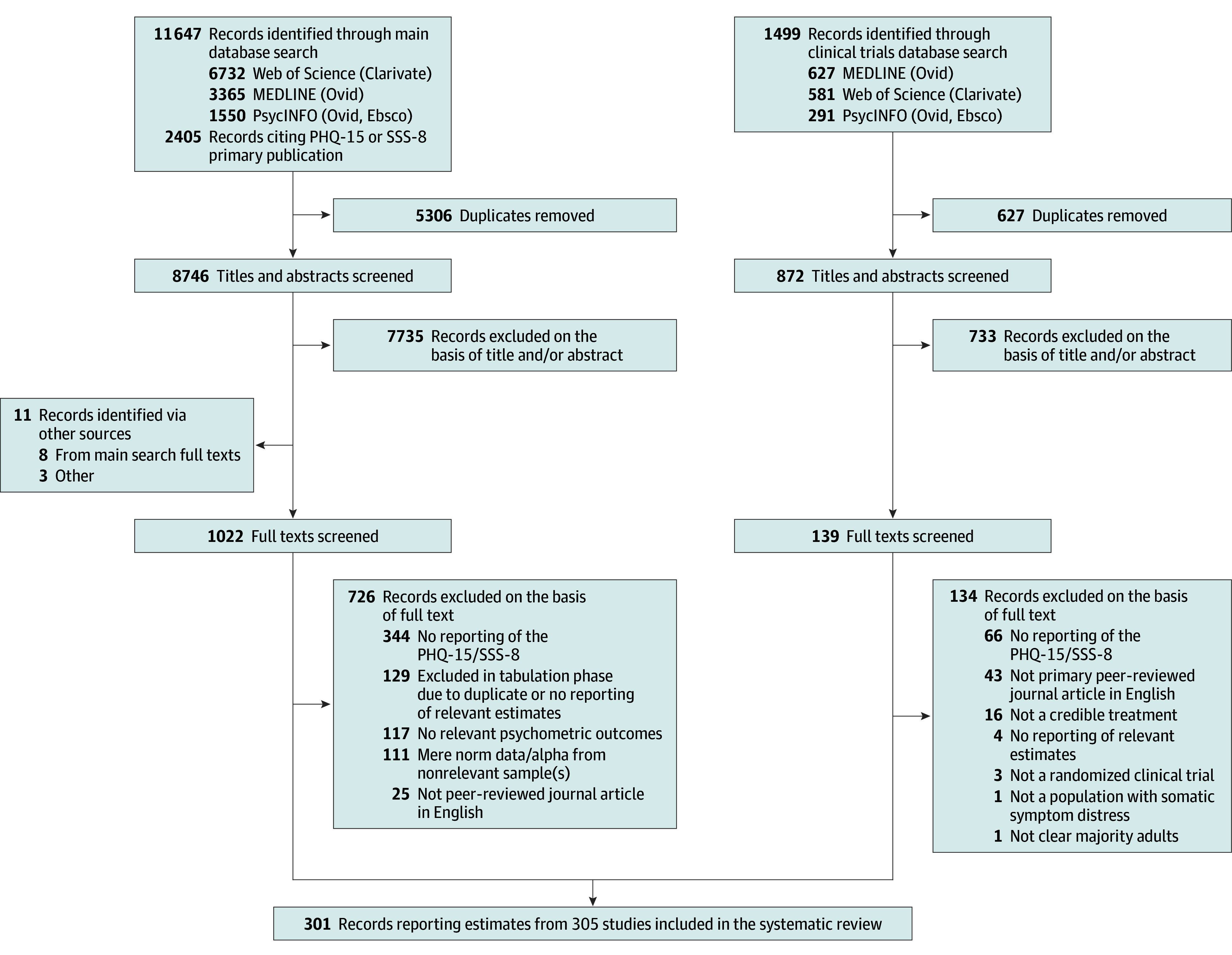
Study Flowchart Illustrating the Identification of Studies Interrater agreement for 2 independent reviewers was excellent (κ = 0.78). PHQ-15 indicates the Patient Health Questionnaire–15; SSS-8, Somatic Symptom Scale–8.

The literature searches identified 15 552 records. After deduplication, we assessed 9618 titles and abstracts, and then 1161 full-text articles. Ultimately, 301 records encompassing 305 studies were included (Figure 1).^13,20,21,40,41,42,43,44,45,46,47,48,49,50,51,52,53,54,55,56,57,58,59,60,61,62,63,64,65,66,67,68,69,70,71,72,73,74,75,76,77,78,79,80,81,82,83,84,85,86,87,88,89,90,91,92,93,94,95,96,97,98,99,100,101,102,103,104,105,106,107,108,109,110,111,112,113,114,115,116,117,118,119,120,121,122,123,124,125,126,127,128,129,130,131,132,133,134,135,136,137,138,139,140,141,142,143,144,145,146,147,148,149,150,151,152,153,154,155,156,157,158,159,160,161,162,163,164,165,166,167,168,169,170,171,172,173,174,175,176,177,178,179,180,181,182,183,184,185,186,187,188,189,190,191,192,193,194,195,196,197,198,199,200,201,202,203,204,205,206,207,208,209,210,211,212,213,214,215,216,217,218,219,220,221,222,223,224,225,226,227,228,229,230,231,232,233,234,235,236,237,238,239,240,241,242,243,244,245,246,247,248,249,250,251,252,253,254,255,256,257,258,259,260,261,262,263,264,265,266,267,268,269,270,271,272,273,274,275,276,277,278,279,280,281,282,283,284,285,286,287,288,289,290,291,292,293,294,295,296,297,298,299,300,301,302,303,304,305,306,307,308,309,310,311,312,313,314,315,316,317,318,319,320,321,322,323,324,325,326,327,328,329,330,331,332,333,334,335,336,337^ Interrater agreement was excellent (κ = 0.78). eFigure 1 in Supplement 1 illustrates studies included per year of publication.

### Study Characteristics

This review included data from 361 243 participants (60% women; mean [SD] age, 47 [18] years). The studies were from 44 countries in total, mainly Germany (74 studies), the United States (45 studies), China (24 studies), the United Kingdom (21 studies), South Korea (16 studies), Australia (11 studies), Iran (11 studies), and the Netherlands (11 studies). Sampling strategies included secondary and tertiary care (99 studies), psychiatry (35 studies), general population (27 studies), primary care (27 studies), psychosomatic clinics (17 studies; see Zipfel et al^338^), and students (10 studies). A total of 247 studies concerned the PHQ-15 only,^13,20,40,41,42,43,44,45,46,47,48,49,50,52,53,54,55,56,57,58,59,61,62,63,64,65,67,68,69,70,71,72,73,74,75,76,77,78,80,81,82,83,84,85,87,88,89,90,91,93,94,95,97,98,99,101,102,103,104,107,108,109,111,112,114,116,117,118,119,120,121,122,124,125,126,127,128,129,130,132,133,134,136,137,138,139,140,141,142,143,144,145,146,147,149,150,151,152,153,156,157,158,159,160,161,162,163,164,167,168,169,170,173,174,176,177,178,179,180,181,182,183,184,185,188,189,190,191,193,194,195,196,197,198,199,200,201,202,203,205,206,207,208,209,210,211,213,216,217,218,219,220,221,222,224,225,226,227,228,229,230,231,232,233,234,235,236,238,241,242,243,244,245,246,247,249,250,251,253,254,255,256,257,258,259,260,261,262,264,265,266,267,268,269,270,271,272,273,274,275,276,277,278,279,280,281,282,283,284,285,287,289,290,293,294,295,296,297,298,299,300,302,304,305,310,311,313,317,318,320,321,322,323,325,326,328,329,330,331,332,333,334,337^ 52 the SSS-8 only,^21,51,66,79,86,92,96,100,105,106,113,115,123,131,135,148,154,155,165,166,171,175,186,187,192,204,214,215,223,237,239,240,252,263,286,288,291,292,301,303,306,307,308,309,312,314,316,319,324,327,335,336^ and 6 concerned both.^60,110,172,212,248,315^ eTable 4 in Supplement 1 presents additional study characteristics.

### Quality and Risk of Bias

**Figure 2.**
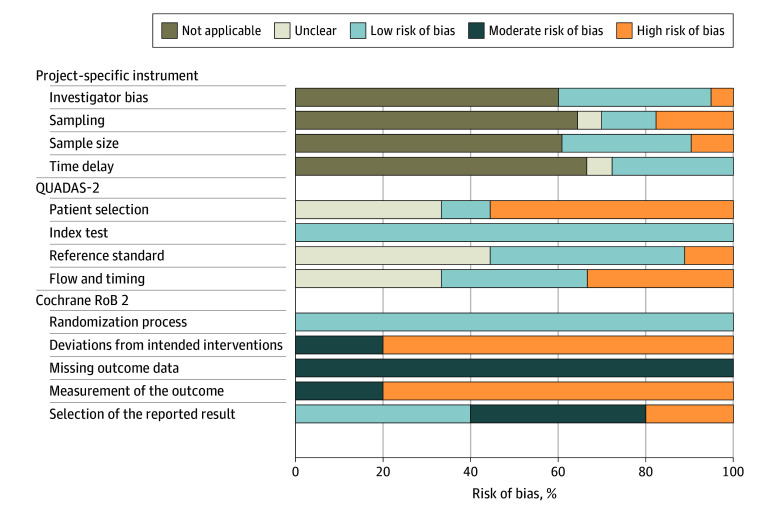
Study Risk of Bias Assessment of study quality and risk of bias was based on 3 structured instruments. The project-specific instrument was used to evaluate all included studies, while Quality Assessment of Diagnostic Accuracy Studies (QUADAS-2) was used for evaluation of studies reporting cutoffs, and the Cochrane Risk of Bias version 2 (Cochrane RoB 2) tool was used to assess RCTs included for investigation of sensitivity to change.

The interrater reliability of the project-specific instrument was nearly perfect (κ = 0.95-1.00). Risk of bias was indicated primarily with respect to sampling method, typically due to studies not establishing psychiatric disorders based on a structured interview. Interrater reliability of the QUADAS-2 was substantial (κ = 0.66-1.00). Risk of bias was indicated primarily in terms of selection bias, typically due to missing data or nonrepresentative sampling. The interrater reliability of the RoB 2 domains was moderate (weighted κ = 0.58). Risk of bias in the clinical trials was indicated primarily in terms of measurement strategy, primarily the lack of blinding. Figure 2 presents an overview of all risk-of-bias ratings.

### Measurement Properties of the PHQ-15 and SSS-8

#### Latent Structure of Subjective Somatic Symptom Burden: Factor and Taxometric Analyses

**Figure 3.**
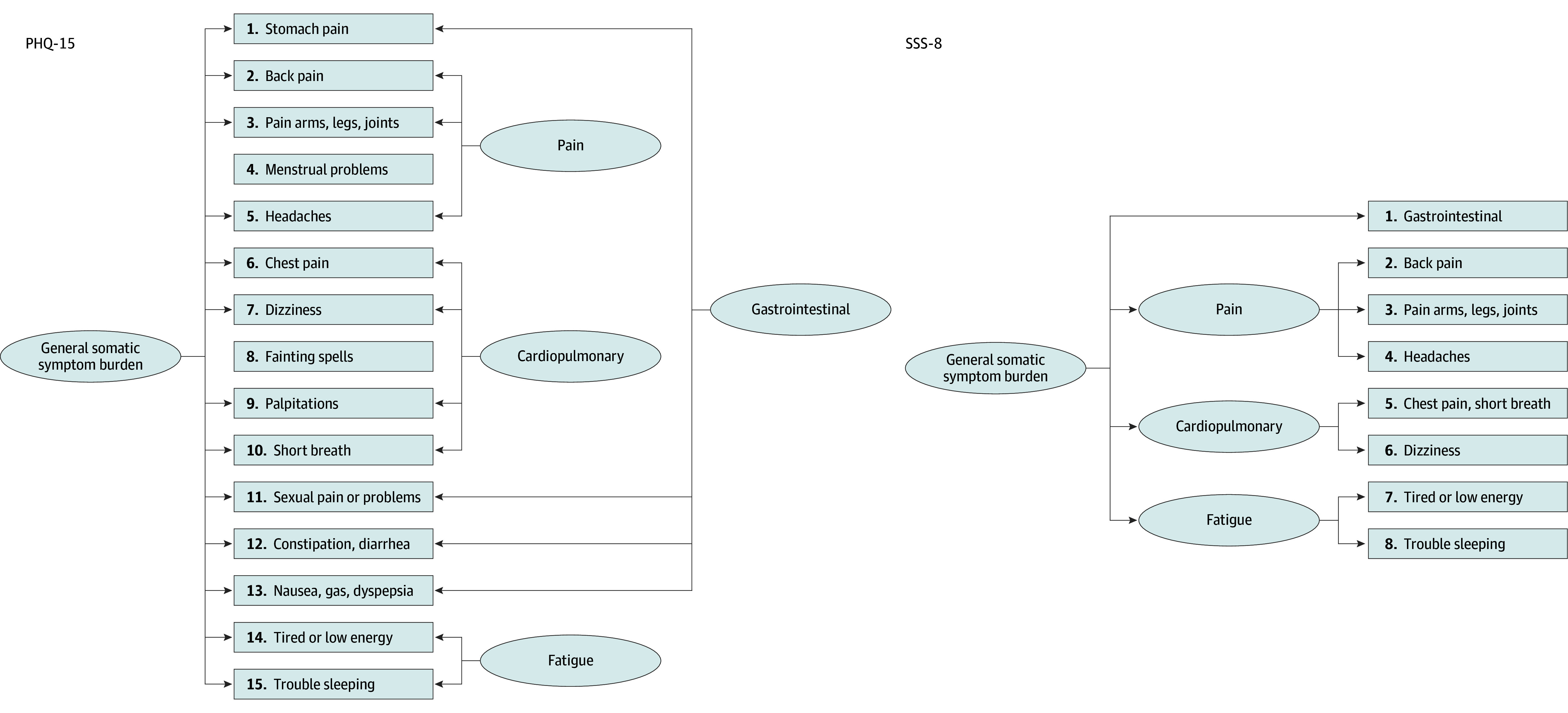
Factor Structure of the Patient Health Questionnaire–15 (PHQ-15) and Somatic Symptom Scale–8 (SSS-8) This figure illustrates the typical factor structure of the PHQ-15 and SSS-8 among included studies. For the PHQ-15, items 4 (menstrual problems) and 8 (fainting spells) were not included in the general somatic symptom burden. An overview of all factor analyses is provided in eTables 5 to 8 in Supplement 1.

For the PHQ-15, a bifactor model similar to that proposed by Witthöft et al^295^ exhibited the best model fit in a clear majority of studies where such a model was evaluated (13 studies^83,84,95,145,170,178,254,295,328^ of 16 total studies^61,83,84,95,145,170,178,254,295,328,332^) (Figure 3). In factor analyses that did not allow for a bifactor or hierarchical structure, it was more common with multifactorial (15 studies of 16 total studies^57,64,101,119,177,229,232,233,299,302^) than unifactorial solutions. For the SSS-8, a hierarchical model similar to that proposed by Gierk et al^21^ was the best-fitting model in 3 studies^21,166,315^ and showed an equally good fit as a unifactorial model in 1 study^301^ of 5 relevant studies^21,166,213,301,315^ (Figure 3). A unifactorial model was also deemed adequate in 3 studies^171,192,316^ of 5 total studies^171,192,213,301,316^ where this was evaluated. The average variance extracted (AVE) for the general somatic symptom burden factor was 0.19 to 0.45 for the PHQ-15 and 0.30 to 0.51 for the SSS-8. eTables 5 to 8 in Supplement 1 provide more information about the factor analyses. Two studies^119,339^ reported taxometric analyses of the PHQ-15 and indicated that general somatic symptom burden is best regarded as a dimensional phenomenon, differing by degree (rather than categories) throughout the population (14 of 15 comparison curve fix indices, <0.50).

#### Internal Consistency

Based on Cronbach α, internal consistency was good with minimal heterogeneity (PHQ-15: overall pooled α = 0.81 [95% CI, 0.80-0.82]; 89 studies; τ^2^ = 0.01; *I*^2^ = 96%; SSS-8: overall pooled α = 0.80 [95% CI, 0.77-0.83]; 20 studies; τ^2^ = 0.01; *I^2^* = 96%). Sensitivity analyses based on the English-language versions only and secondary pooled αs per setting and in various diagnoses appear in eTable 9 in Supplement 1. Item-total correlations were high for the SSS-8, but low (<0.40) for PHQ-15 items that concerned menstrual problems, fainting spells, and sexual problems (eTables 10 and 11 in Supplement 1).

#### Pooled Correlations and Mean Scores Relevant for Construct Validity

Pooled Pearson correlations with other key clinical characteristics are reported in Table 1. Sensitivity analyses in various settings, with *z* transformation, with English-language studies only, and excluding studies at risk of bias due to time delay showed predominantly similar results (eTables 12 and 13 in Supplement 1). Mean scores in various settings and diagnostic groups ranged from 4.1 to 15.1 for the PHQ-15, with high heterogeneity, and the corresponding SSS-8 mean scores varied from 6.9 to 14.7, also with high heterogeneity (Table 2). Sensitivity analyses of pooled mean scores when excluding studies deemed at high risk of bias due to sampling method showed largely similar results, which was also the case for analyses based solely on the English-language versions of the scales (eTable 14 in Supplement 1).

**Table 1.** Pooled Pearson Correlations vs Other Measures Relevant for Construct Validity

Construct	Studies, No.^a^	Pooled *r* (95% CI)	Heterogeneity statistics		
			τ^2^	*I*^2^, %	*Q* (df)
**PHQ-15**					
Somatic symptom burden^b^	9	0.71 (0.64-0.78)	0.01	84%	59.7 (8)^c^
Health anxiety^d^	11	0.52 (0.40-0.65)	0.01	94%	293.5 (10)^c^
Symptom preoccupation^e^	5	0.45 (0.39-0.51)	<0.01	83%	30.3 (4)^c^
General anxiety	34	0.54 (0.49-0.59)	0.01	87%	279.7 (33)^c^
Depression	53	0.62 (0.56-0.67)	0.01	95%	1020.0 (52)^c^
**SSS-8**					
Somatic symptom burden^b^	7	0.82 (0.72-0.92)	0.01	93%	136.2 (6)^c^
Health anxiety^d^	3	0.59 (0.54-0.64)	<0.01	14%	3.7 (2)
Symptom preoccupation^e^	6	0.66 (0.53-0.79)	0.01	95%	171.1 (5)^c^
General anxiety	13	0.55 (0.50-0.60)	<0.01	62%	107.4 (12)^c^
Depression	18	0.52 (0.42-0.61)	<0.01	93%	516.0 (17)^c^

Abbreviations: PHQ-15, Patient Health Questionnaire–15; SSS-8, Somatic Symptom Scale–8.

^a^ Number of studies included in random-effects meta-analysis.

^b^ Somatic symptom burden refers to other measures that focus on the subjective experience of physical symptoms.

^c^ *P* < .05.

^d^ Health anxiety refers to the fear of, or preoccupation with, having or developing a serious disease.

^e^ Symptom preoccupation refers to the tendency to respond strongly to, and engage in behaviors contingent on, somatic symptoms.

**Table 2.** Meta-Analysis of Mean Scores per Setting and Condition^a^

Setting	PHQ-15					SSS-8				
	Studies, No.	Pooled mean (95% CI)^a^	τ^2^	*I*^2^, %	*Q* (df)	Studies, No.	Pooled M (95% CI)^a^	τ^2^	*I*^2^, %	*Q* (df)
General population^b^	13	4.7 (3.8-5.6)	2.7	100	2355.9 (12)^c^	8	6.9 (4.0-9.8)	17.3	100	41245.2 (7)^c^
Low risk of bias sampling only	2	4.9 (2.3-7.6)	3.6	100	970.2 (1)^c^	2	6.5 (1.3-11.7)	14.0	100	288.8 (1)^c^
Weighted^d^	12	4.1 (2.4-5.7)	3.0	100	2291.3 (10)^c^	8	7.8 (2.9-12.8)	19.5	100	39491.3 (6)^c^
Primary care, general	11	10.4 (7.7-13.1)	21.4	100	2034.2 (10)^c^	1	NA	NA	NA	NA
Primary care, mental health	13	12.3 (11.0-13.6)	5.5	99	1070.6 (12)^c^	1	NA	NA	NA	NA
Psychiatry	26	10.7 (9.7-11.6)	5.8	99	897.9 (25)^c^	7	12.0 (11.5-12.6)	0.0	0	5.0 (6)
Psychosomatic specialist	11	12.7 (11.5-13.9)	3.8	96	279.2 (10)^c^	5	13.7 (8.5-18.9)	34.8	99	356.9 (4)^c^
Other medical care	74	10.4 (9.7-11.2)	9.9	99	11392.3 (73)^c^	18	11.8 (9.3-14.3)	29.3	100	2408.4 (17)^c^
Functional symptoms or syndromes	45	12.4 (11.4-13.3)	9.8	99	4918.1 (44)^c^	8	10.4 (6.7-14.2)	29.0	100	578.1 (7)^c^
Persistent pain, any	26	11.0 (9.8-12.3)	10.3	100	6019.3 (25)^c^	7	8.9 (7.7-10.0)	2.2	98	87.4 (6)^c^
Persistent physical symptoms	5	12.4 (10.0-14.8)	7.4	99	167.7 (4)^c^	1	NA	NA	NA	NA
Organic disease, any	29	9.3 (8.4-10.3)	6.5	99	3074.5 (28)^c^	8	8.9 (8.1-9.8)	1.2	92	76.9 (7)^c^
Functional somatic syndromes										
Fibromyalgia	5	15.6 (13.4-17.9)	6.6	99	261.4 (4)^c^	2	NA	NA	NA	NA
Irritable bowel syndrome	8	12.6 (10.4-14.7)	9.8	99	2405.2 (7)^c^	1	NA	NA	NA	NA
Noncardiac chest pain	3	11.4 (9.9-12.8)	1.4	93	37.1 (2)^c^	0	NA	NA	NA	NA
Persistent pelvic pain	4	8.8 (6.4-11.2)	5.7	96	98.8 (3)^c^	0	NA	NA	NA	NA
Psychiatric disorders^e^										
Anxiety disorder, any	5	12.9 (11.8-14.0)	1.0	76	19.9 (4)^c^	2	NA	NA	NA	NA
Depression	11	11.1 (9.8-12.3)	4.2	98	328.3 (10)^c^	3	11.7 (11.1-12.3)	0.0	0	0.9 (2)
Somatoform disorder, any	24	12.6 (11.7-13.4)	4.3	95	582.4 (23)^c^	7	14.4 (12.2-16.5)	7.7	96	240.3 (6)^c^
Pathological health anxiety	5	12.4 (10.6-14.2)	3.7	92	71.3 (4)^c^	0	NA	NA	NA	NA
Somatic symptom disorder	9	12.4 (10.8-14.1)	6.2	97	312.6 (8)^c^	5	14.7 (11.7-17.7)	10.9	98	221.6 (4)^c^

Abbreviations: NA, not applicable; PHQ-15, Patient Health Questionnaire–15; PTSD, posttraumatic stress disorder; SSS-8, Somatic Symptom Scale–8.

^a^ Meta-analysis was conducted for strata with at least 3 studies, except in the sensitivity analysis based on low risk of bias sampling only. Whenever categories overlapped or subcategories were reported in original studies, the same original study could contribute to multiple pooled estimates. NA indicates that there were too few studies available to pool data.

^b^ Indicators of publication bias for the pooled general population mean on the SSS-8 were contradictory. See eTable 18 in Supplement 1 for further details concerning publication bias.

^c^ *P* < .05.

^d^ This mean was weighted to represent a sample with 50% women.

^e^ Categories for eating disorders and posttraumatic stress disorder were also tabulated, with 2 included PHQ-15 studies^257,325^ and 1 included PHQ-15 study,^141^ respectively. However, due to the included studies being fewer than 3 per stratum, these were not pooled in meta-analysis.

#### Cutoffs to Identify Patients with Somatoform Disorders

For the PHQ-15, the overall AUROC from 9 studies for identification of somatoform disorder ranged from 0.63 (95% CI, 0.50-0.76) for sick-listed employees^329^ to 0.79 (95% CI, 0.73-0.85) for the general population.^251^ Optimal cutoffs, weighting sensitivity and specificity equally, ranged from 6 in the general population to between 11 and 14 in psychosomatic clinics. For the SSS-8, overall AUCs were 0.71 (95% CI, 0.66-0.77) and 0.73 (95% CI, 0.69-0.76) from 2 studies.^110,212^ The optimal weighted cutoff was 9 in a mixed hospital and healthy sample and 14 in a psychosomatic clinic. A full account of the included studies that evaluated cutoffs appears in eTables 15 and 16 in Supplement 1.

#### MCID

Two studies^187,340^ presented estimates of the MCID, ie, the smallest difference of clinical relevance, on the SSS-8 and argued for an MCID of 3. One study^340^ estimated that the MCID for the PHQ-15, although excluding 1 item, was 2.3.^340^

#### Test-Retest Reliability

Few included studies evaluated test-retest reliability. For the PHQ-15, estimates were inconsistent over 10 to 14 days (*r* = 0.65 in a sample of general psychiatry patients^236^; *r* = 0.93 in a mixed sample with unclear reporting of sample size^302^; intraclass correlation [ICC], 0.87 in a small student sample^299^). In the 2 identified relevant publications for the SSS-8,^301,315^ test-retest reliability appeared adequate over 7 to 14 days (*r* = 0.996 in a secondary care pain sample^315^; ICC, 0.89 in a general population sample^301^). eTable 17 in Supplement 1 provides more details.

#### Sensitivity to Change

There was a significant and moderate pooled effect size of CBT vs rudimentary controls on the PHQ-15 in trials for conditions where somatic symptom burden is central (*g* = 0.32 [95% CI, 0.08-0.56]; 5 studies; τ^2^ = 0.04; *I^2^* = 57%) (eFigure 2 in Supplement 1). No study evaluated the corresponding outcome for the SSS-8. One study^341^ reported a correlation between change in the PHQ-15 and change in symptom preoccupation over the course of exposure-based treatment (*r* = 0.68). One study^187^ reported correlations between change on the SSS-8 and change in anxiety (*r* = 0.68), depression (*r* = 0.62), and disability (*r* = 0.51) during treatment for common mental disorders.

#### Threat of Publication Bias

Indicators of possible publication bias were evaluated for the Cronbach α, correlations with other scales, means, and Hedges *g*. Most pooled outcomes appeared robust, an exception being the pooled general population mean on the SSS-8, for which publications bias indicators were contradictory (eTable 18 in Supplement 1).

## Discussion

This systematic review and meta-analysis concerned the measurement properties of 2 widespread patient-reported measures of overall somatic symptom burden: the PHQ-15 and the SSS-8. We included 305 studies with 361 243 participants, based in a variety of clinical and other settings. The PHQ-15 and SSS-8 were found to reflect both domain-specific factors (cardiopulmonary, fatigue, gastrointestinal, pain) and a general somatic symptom burden factor of moderate strength. Both scales exhibited overall adequate internal consistency, and most correlations with other measures were supportive of construct validity. The scales’ accuracy in identifying somatoform disorders was only borderline acceptable. Test-retest reliability over 7 to 14 days was evaluated in a small number of studies and was found to be inconsistent for the PHQ-15. Tentatively, this parameter appeared adequate for the SSS-8, based on 2 relevant studies.^301,315^ The PHQ-15 showed preliminary evidence of being sensitive to change, but data for the SSS-8 are lacking. Overall, most preregistered targets were met. An overview is provided in eTable 19 in Supplement 1.

### Latent Structure of Subjective Somatic Symptom Burden and Its Implications

The latent structure of the PHQ-15 and SSS-8, with 1 general factor, and 3 to 4 domain-specific factors—usually cardiopulmonary, fatigue, gastrointestinal, and pain—appears to be a relatively robust finding that is in line with the broader literature.^342^ Factor loadings indicated that approximately 19% to 45% of the variance in the PHQ-15 and approximately 30% to 51% in the SSS-8 can be explained by the general somatic symptom burden factor. For the purpose of sum scoring, all estimates except those reported in the primary publication for the SSS-8^21^ were lower than the typical target of 50% or greater.^343^ On one hand, this suggests that the PHQ-15 and SSS-8 sums need to be routinely interpreted alongside the distribution over item subtypes (eg, cardiopulmonary, gastrointestinal, fatigue, pain). On the other, the moderately strong general factor also illustrates that domain-specific questionnaires, such as fatigue- or pain-only instruments, are likely to capture phenomena that are common to a much broader set of physical symptoms. This lends some support for both lumping and splitting perspectives on persistent physical symptoms.^344,345^ Mechanistic studies focusing on the general tendency of being symptomatic would be recommended to consider modeling strategies that recognize both general and domain-specific contributions to the patient’s health state. In routine care, the PHQ-15 and SSS-8 enable a broad overview of somatic symptom burden, but clinicians are advised to complement the sum score with a review of the distribution of individual symptom domains that dominate the clinical presentation for the specific patient.

### Cutoffs to Identify Somatoform Disorders

Focusing on the identification of somatoform disorders, the screening ability of the PHQ-15 and SSS-8 was only borderline acceptable. We can also note that the widely used cutoff of 10 was close to the pooled mean score in many health care settings (Table 2). As with all clinimetric instruments, the choice of cutoff should be informed by its purpose—such as to identify all cases or to achieve the highest possible correct classification rate—and the setting in which it is used.^346^ Generally speaking, however, based on the existing evidence base, the sum scores on the PHQ-15, and tentatively also the SSS-8, appear to be of limited use for the identification of somatoform disorders.

### Study of Change Using the PHQ-15 and SSS-8

To enable reliable measurement of somatic symptom burden in routine care and clinical trials, instruments need to be able to detect change. It is also important to know what constitutes the smallest clinically relevant change. Based on the present study, there is tentative evidence that the PHQ-15 can detect change in somatic symptom burden caused by treatment. For the SSS-8, only 1 study^187^ of relevance for sensitivity to change could be identified. Even though the results were promising, further evaluation is warranted. Regarding the smallest difference in sum score considered clinically relevant, the results suggest 2.3 points (in practice, 3 points) for the PHQ-15 and 3 points for the SSS-8. In summary, the current evidence base is cautiously supportive of further use of the PHQ-15 and SSS-8 in the study of change in somatic symptom burden.

### Comparison of the PHQ-15 and the SSS-8

In this systematic review, the PHQ-15 and SSS-8 performed about equally well in most regards. Advantages of the PHQ-15 include more substantial reference data and that there is arguably more existing evidence of sensitivity to change. Advantages of the SSS-8 include its brief format without seemingly redundant items and that it was developed with many response options and a 1-week time frame to facilitate repeated administration, something that has not yet frequently been evaluated on an empirical basis. At the present point in time, no unanimous recommendation concerning which scale is preferable across settings and applications can be given. While the PHQ-15 could be the better choice when this facilitates comparisons with previous studies and does not require new translations, the SSS-8 is likely preferable when quick administration is of particular importance.

### Limitations

This study had limitations. Concerning the search strategy, had we surveyed additional databases such as CENTRAL or Embase or added non–English language publications, this would have further expanded the scope of the present work. On the other hand, this review included more than 300 studies and tabulated data from more than one-third of a million study participants. Cultural and language differences can affect measurement properties, and most likely added to the variance seen in this review.^347^ That stated, sensitivity analyses including only English-version scales resulted in largely similar results. Furthermore, results from factor analyses have supported the existence of a general somatic symptom factor across cultures,^95^ and this systematic review focused clearly on this general factor as opposed to the symptom-specific factors. Additionally, most included RCTs indicative of sensitivity to change were at high risk of bias due to threats to the measurement strategy,^36^ in part caused by the lack of blinding, and it is possible that the association of CBT with changes in symptom burden may also reflect expectancy effects. Furthermore, this systematic review did not survey the acceptability or clinical utility of the scales in the eyes of patients and clinicians. These aspects are also important and could be reviewed in future studies.

## Conclusions

This comprehensive systematic review and meta-analysis supports the use and facilitates the interpretation of the PHQ-15 and SSS-8 across various health care settings. These instruments capture both general and domain-specific aspects of somatic symptom burden. Clinicians need to be aware that sum scores reflect a complex interplay of biological, psychological, and social factors, which necessitates careful interpretation of item distributions. Additional empirical investigation is warranted concerning test-retest reliability and sensitivity to change.

## Notes

Supplement 1. **eAppendix 1.** Elaboration on Hypotheses and Benchmarking Targets **eAppendix 2.** Search Strategy **eFigure 1.** Studies Included per Publication Year **eAppendix 3.** Supplementary Methods **eTable 1.** Methods of Meta-Analysis, as Specified in the R Metafor Package **eTable 2.** Coding Scheme for the Assessment of Risk of Bias, Loosely Based on Reilly et al (2015) **eTable 3.** Coding Scheme for Medical Conditions, Used for the Pooling of Means and Cronbach α **eTable 4.** Study Characteristics **eTable 5.** Factor Analyses of the Patient Health Questionnaire–15 **eTable 6.** Model Fit in Factor Analyses of the Patient Health Questionnaire–15 **eTable 7.** Factor Analyses of the Somatic Symptom Scale–8 **eTable 8.** Average Variance Extracted (AVE)a Pertaining to the General Somatic Symptom Burden Factor **eTable 9.** Meta-Analysis of Cronbach α per Language, Setting, and Condition **eTable 10.** Item-Total Correlations for the Patient Health Questionnaire–15 **eTable 11.** Item-Total Correlations for the Somatic Symptom Scale–8 **eTable 12.** Sensitivity Analyses Focusing on Pearson Correlations Relevant for Construct Validity **eTable 13.** Sensitivity Analyses Focusing on Pearson Correlations vs Other Constructs: Low Risk of Bias **eTable 14.** Sensitivity Analyses of Mean Scores Based on the English Version and Low Risk of Bias Due to Sampling Only **eTable 15.** Clinical Cutoffs on the PHQ-15 According to Setting **eTable 16.** Clinical Cutoffs on the SSS-8 According to Setting **eTable 17.** Test-Retest Reliability **eFigure 2.** Effect Sizes for Cognitive-Behavioral Therapy Compared With Rudimentary Controls on Somatic Symptom Burden, as Measured Using the PHQ-15 in Somatoform Conditions and Functional Somatic Symptoms and Syndromes **eTable 18.** Indicators of Possible Publication Bias **eTable 19.** Summary of Targets, Hypotheses, Outcomes, and Conclusions
eReferences.

Supplement 2.
Data Sharing Statement
