# Error in Figure 3

**DOI:** 10.1001/jamanetworkopen.2025.13410

**Published:** 2025-04-29

In the Original Investigation titled “Measurement Properties of the Patient Health Questionnaire–15 and Somatic Symptom Scale–8: A Systematic Review and Meta-Analysis,”^1^ published November 20, 2024, there was an error in Figure 3. In the right-hand panel, there should have been an arrow between “Pain” and “Headaches,” not between “Cardiopulmonary” and “Headaches.” This article has been corrected.^1^
