# Supplementary material for: Measurement Properties of the Patient Health Questionnaire–15 and Somatic Symptom Scale–8: A Systematic Review and Meta-Analysis
Source: JAMA Netw Open. 2024 Nov 20;7(11):e2446603. doi: 10.1001/jamanetworkopen.2024.46603 (PMC11579800; doi:10.1001/jamanetworkopen.2024.46603)
Supplement: Supplement 1. — eAppendix 1. Elaboration on Hypotheses and Benchmarking Targets eAppendix 2. Search Strategy eFigure 1. Studies Included per Publication Year eAppendix 3. Supplementary Methods eTable 1. Methods of Meta-Analysis, as Specified in the R Metafor Package eTable 2. Coding Scheme for the Assessment of Risk of Bias, Loosely Based on Reilly et al (2015) eTable 3. Coding Scheme for Medical Conditions, Used for the Pooling of Means and Cronbach α eTable 4. Study Characteristics eTable 5. Factor Analyses of the Patient Health Questionnaire–15 eTable 6. Model Fit in Factor Analyses of the Patient Health Questionnaire–15 eTable 7. Factor Analyses of the Somatic Symptom Scale–8 eTable 8. Average Variance Extracted (AVE)a Pertaining to the General Somatic Symptom Burden Factor eTable 9. Meta-Analysis of Cronbach α per Language, Setting, and Condition eTable 10. Item-Total Correlations for the Patient Health Questionnaire–15 eTable 11. Item-Total Correlations for the Somatic Symptom Scale–8 eTable 12. Sensitivity Analyses Focusing on Pearson Correlations Relevant for Construct Validity eTable 13. Sensitivity Analyses Focusing on Pearson Correlations vs Other Constructs: Low Risk of Bias eTable 14. Sensitivity Analyses of Mean Scores Based on the English Version and Low Risk of Bias Due to Sampling Only eTable 15. Clinical Cutoffs on the PHQ-15 According to Setting eTable 16. Clinical Cutoffs on the SSS-8 According to Setting eTable 17. Test-Retest Reliability eFigure 2. Effect Sizes for Cognitive-Behavioral Therapy Compared With Rudimentary Controls on Somatic Symptom Burden, as Measured Using the PHQ-15 in Somatoform Conditions and Functional Somatic Symptoms and Syndromes eTable 18. Indicators of Possible Publication Bias eTable 19. Summary of Targets, Hypotheses, Outcomes, and Conclusions eReferences. [file jamanetwopen-e2446603-s001.pdf]

Supplemental Online Content

Hybelius J, Kosic A, Salomonsson S, et al. Measurement properties of the Patient Health Questionnaire–15 and Somatic Symptom Scale–8: a systematic review and meta-analysis. *JAMA Netw Open.* 2024;7(11):e2446603. doi:10.1001/jamanetworkopen.2024.46603

eAppendix 1. Elaboration on Hypotheses and Benchmarking Targets

eAppendix 2. Search Strategy

eFigure 1. Studies Included per Publication Year

eAppendix 3. Supplementary Methods

eTable 1. Methods of Meta-Analysis, as Specified in the R Metafor Package

eTable 2. Coding Scheme for the Assessment of Risk of Bias, Loosely Based on Reilly et al (2015)

eTable 3. Coding Scheme for Medical Conditions, Used for the Pooling of Means and Cronbach α

eTable 4. Study Characteristics

eTable 5. Factor Analyses of the Patient Health Questionnaire–15

eTable 6. Model Fit in Factor Analyses of the Patient Health Questionnaire–15

eTable 7. Factor Analyses of the Somatic Symptom Scale–8

eTable 8. Average Variance Extracted (AVE)a Pertaining to the General Somatic Symptom Burden Factor

eTable 9. Meta-Analysis of Cronbach α per Language, Setting, and Condition

eTable 10. Item-Total Correlations for the Patient Health Questionnaire–15

eTable 11. Item-Total Correlations for the Somatic Symptom Scale–8

eTable 12. Sensitivity Analyses Focusing on Pearson Correlations Relevant for Construct Validity

eTable 13. Sensitivity Analyses Focusing on Pearson Correlations vs Other Constructs: Low Risk of Bias

eTable 14. Sensitivity Analyses of Mean Scores Based on the English Version and Low Risk of Bias Due to Sampling Only

eTable 15. Clinical Cutoffs on the PHQ-15 According to Setting

eTable 16. Clinical Cutoffs on the SSS-8 According to Setting

eTable 17. Test-Retest Reliability

eFigure 2. Effect Sizes for Cognitive-Behavioral Therapy Compared With Rudimentary Controls on Somatic Symptom Burden, as Measured Using the PHQ-15 in Somatoform Conditions and Functional Somatic Symptoms and Syndromes

eTable 18. Indicators of Possible Publication Bias

eTable 19. Summary of Targets, Hypotheses, Outcomes, and Conclusions

eReferences.

This supplemental material has been provided by the authors to give readers additional information about their work.

eAppendix 1. Elaboration on Hypotheses and Benchmarking Targets

The Patient Health Questionnaire-15 (PHQ-15) and Somatic Symptom Scale-8 (SSS-8): A Systematic Review (PROSPERO: CRD42022342827)

Factor structure

PHQ-15: Expected to be in support of a bifactor model, with one general factor and four domain-specific factors (gastrointestinal, pain, fatigue, cardiopulmonary).[1,2]

SSS-8: Expected to be in support of a higher-order general factor model with an overarching somatic symptom factor, and four domain-specific factors (gastrointestinal, pain, cardiopulmonary, fatigue).[3]

Internal consistency

PHQ-15 and SSS-8: Expected to be adequate, as indicated by Cronbach's αs ≥ ca 0.70, and most item-total correlations ≥ ca 0.4-0.5.

Correlations versus (a) other measures of somatic symptom burden or symptom preoccupation, (b) anxiety, (c) depression, (d) functional impairment, and I healthcare use

PHQ-15 and SSS-8: Based on the original publication,[4] we expect the following:

- Pearson correlations ≈ 0.40-0.85 versus other measures of somatic symptom burden or symptom preoccupation. In the higher end of the range versus other measures of somatic symptom burden (i.e., the SCL-90[5]). In the lower end of the range versus specific types of symptom preoccupation, such as trait health anxiety (i.e., the HAI[6]).

- Pearson correlations ≈ 0.30-0.60 versus anxiety, depression, functional impairment, and healthcare use.

© 2024 Hybelius J et al. *JAMA Network Open.*

Pooled mean scores (norms) for various clinical settings and patient groups

PHQ-15 and SSS-8: We expect to see pooled mean scores similar to the norms reported in the early and original publications. For example, a pooled general population PHQ-15 mean close to 3.8 (± 4.1) and an SSS-8 mean of 3.23 (±3.96).[3,7]

Cut-offs to identify patients with substantial somatic symptom distress, such as the functional somatic syndromes and somatic symptom and related disorders

PHQ-15: Based on the original publication[4] and depending on the precise application, we expect cut-offs to approximate guidelines for at least moderate (ca 10+), or more likely high (ca 15+) sum scores.

SSS-8: Based on the original publication[3] and depending on the precise application, we expect cut-offs to approximate guidelines for at least high (ca 12+), or possibly very high (ca 16+) sum scores.

Criteria for a minimal clinically important difference, and reliable change on an individual basis

Exploratory research question.

Test-retest reliability

PHQ-15 and SSS-8: Expected to be adequate. Considering that somatic symptom burden is typically conceived of as moderately stable over time,[3,4,8,9] adequate parameter estimates over ca two weeks should approximate rs $\geq$ ca 0.70 and ICCs $\geq$ 0.50, ideally $\geq$ 0.75.[10,11]

Sensitivity to change (responsiveness)

PHQ-15 and SSS-8: Expected to be adequate, as evidenced by a significant and moderately sized ($g \geq$ ca 0.35) pooled effect of cognitive behavioral therapy versus rudimentary waitlist and usual care controls for patients with somatic symptom distress in randomized controlled trials.[12,13] Also, moderate correlations, $r \approx 0.30\text{-}0.70$, in change versus change in other measures of somatic symptom burden or symptom preoccupation.

© 2024 Hybelius J et al. *JAMA Network Open.*

References

1. Witthoft M, Hiller W, Loch N, Jasper F. The latent structure of medically unexplained symptoms and its relation to functional somatic syndromes. Int J Behav Med. Jun;20(2):172–83.

2. Witthoft M, Fischer S, Jasper F, Rist F, Nater UM. Clarifying the latent structure and correlates of somatic symptom distress: A bifactor model approach. Psychol Assess. Jan;28(1):109–15.

3. Gierk B, Kohlmann S, Kroenke K, Spangenberg L, Zenger M, Brähler E, et al. The Somatic Symptom Scale–8 (SSS-8): A Brief Measure of Somatic Symptom Burden. JAMA Intern Med. 2014 Mar 1;174(3):399–407.

4. Kroenke K, Spitzer RL, Williams JBW. The PHQ-15: Validity of a New Measure for Evaluating the Severity of Somatic Symptoms. Psychosom Med. 2002 Apr;64(2):258–66.

5. Derogatis LR, Rickels K, Rock AF. The SCL-90 and the MMPI: A Step in the Validation of a New Self-Report Scale. Br J Psychiatry. 1976 Mar;128(3):280–9.

6. Salkovskis PM, Rimes KA, Warwick HMC, Clark DM. The Health Anxiety Inventory: development and validation of scales for the measurement of health anxiety and hypochondriasis. Psychol Med. 2002 Jul;32(5):843–53.

7. Kocalevent RD, Hinz A, Brähler E. Standardization of a screening instrument (PHQ-15) for somatization syndromes in the general population. BMC Psychiatry. 2013 Mar 20;13(1):91.

8. Atasoy S, Henningsen P, Sattel H, Baumert J, Rückert-Eheberg IM, Kraus U, et al. Stability and predictors of somatic symptoms in men and women over 10 years: A real-world perspective from the prospective MONICA/KORA study. J Psychosom Res. 2022 Nov;162:111022.

9. American Psychiatric Association. Somatic Symptom and Related Disorders. In: Diagnostic and Statistical Manual of Mental Disorders. Arlington, VA: American Psychiatric Association; 2013. 315–8.

10. Ahn S, Myers ND, Jin Y. Use of the estimated intraclass correlation for correcting differences in effect size by level. Behav Res Methods. 2012 Jun 1;44(2):490–502.

11. Koo TK, Li MY. A Guideline of Selecting and Reporting Intraclass Correlation Coefficients for Reliability Research. J Chiropr Med. 2016 Jun;15(2):155–63.

12. Liu J, Gill NS, Teodorczuk A, Li Z jiang, Sun J. The efficacy of cognitive behavioural therapy in somatoform disorders and medically unexplained physical symptoms: A meta-analysis of randomized controlled trials. J Affect Disord. 2019 Feb;245:98–112.

13. Vugts MAP, Joosen MCW, van der Geer JE, Zedlitz AMEE, Vrijhoef HJM. The effectiveness of various computer-based interventions for patients with chronic pain or functional somatic syndromes: A systematic review and meta-analysis. Yang J, editor. PLOS ONE. 2018 May 16;13(5):e0196467.

© 2024 Hybelius J et al. *JAMA Network Open.*

eAppendix 2. Search Strategy

Documentation of search strategies
University Library search consultation group

Date: 1 February 2024

Topic/research question: PHQ-15 / SSS-8

Name of researcher(s): Erland Axelsson, Jonna Hybelius

Librarian(s): Emma-Lotta Säätelä

Databases:

1. Ovid (Medline)
2. Web of Science Core Collection (Clarivate)
3. Psycinfo (Ebsco)

Total number of hits

- Before deduplication: 11,647
- After deduplication: 3,069 (+ 4,626 from previous search)

Comments:

After the original search was performed on 9 October 2021, the search was last updated on 1 February 2024 using the methods described by Bramer et al (1).

De-duplication was done using the method described by Bramer et al (2). One final, extra step was added to compare DOIs.

References

1. Bramer W, Bain P. (2017). Updating search strategies for systematic reviews using EndNote. *Journal of the Medical Library Association: JMLA*, 105(3):285-289. doi: 10.5195/jmla.2017.183.
2. Bramer, W. M., Giustini, D., de Jonge, G. B., Holland, L., & Bekhuis, T. (2016). De-duplication of database search results for systematic reviews in EndNote. *Journal of the Medical Library Association: JMLA*, 104(3), 240-243. doi: 10.3163/1536-5050.104.3.014

1. Medline

Interface: Ovid MEDLINE(R) ALL<br><br>Date of Search: 1 February 2024<br><br>Number of hits: 3,365<br><br>Comment: In Ovid, two or more words are automatically searched as phrases; i.e. no quotation marks are needed	Field labels<br><br>• exp/ = exploded MeSH term<br>• / = non exploded MeSH term<br>• .ti,ab,kf. = title, abstract and author keywords<br>• adjx = within x words, regardless of order<br>• * = truncation of word for alternate endings

Database(s): **Ovid MEDLINE(R) ALL** 1946 to January 26, 2024
Search Strategy:

#	Searches	Results
1	exp Patient Health Questionnaire/	976
2	("patient health questionnaire*" or PHQ or "somatic symptom scale 8" or SSS-8).ti,ab,kf.	14321
3	1 or 2	14612
4	exp "Factor Analysis, Statistical"/ or exp Psychometrics/ or exp "Reproducibility of Results"/ or exp "Sensitivity and Specificity"/	1042064
5	(psychometric* or valid* or factor analy* or sensitivity or specificity or test-retest).ti,ab,kf.	2290510
6	4 or 5	2888177
7	3 and 6	3365

© 2024 Hybelius J et al. *JAMA Network Open.*

2. Web of Science Core Collection

Interface: Clarivate Analytics<br><br>Editions = A&HCI , ESCI , SCI-EXPANDED , SSCI<br><br>Date of Search: 1 February 2024<br><br>Number of hits: 6,732	Field labels<br><br>• TS/Topic = title, abstract, author keywords and Keywords Plus<br>• NEAR/x = within x words, regardless of order<br>• * = truncation of word for alternate endings<br><br>Note: the *Exact search*-function was used for all the searches

#	Search Query	Results
1	TS=("patient health questionnaire*" or PHQ or "somatic symptom scale 8" or SSS-8)	15839
2	TS=(psychometric* or valid* or "factor analy*" or sensitivity or specificity or "test-retest")	4033364
3	#2 AND #1	6732

© 2024 Hybelius J et al. *JAMA Network Open.*

3. Psycinfo

Interface: EBSCO<br><br>Date of Search: 1 Februay 2024<br><br>Number of hits: 1,550	Field labels<br><br>• DE = subject heading<br>• TI = title<br>• AB = abstract<br>• KW = author keywords<br>• Nx = within x words, regardless of order<br>• * = truncation of word for alternate endings<br><br>Note: the *Apply equivalent subjects*-function was used for all the searches

#	Query	Results
S5	S1 AND S4	1,550
S4	S2 OR S3	658,123
S3	TI (psychometric* or valid* or "factor analy*" or sensitivity or specificity or "test-retest") OR AB (psychometric* or valid* or "factor analy*" or sensitivity or specificity or "test-retest") OR KW (psychometric* or valid* or "factor analy*" or sensitivity or specificity or "test-retest")	499,081
S2	DE "Psychometrics" OR DE "Classical Test Theory" OR DE "Consistency (Measurement)" OR DE "Error of Measurement" OR DE "External Validity" OR DE "Factor Analysis" OR DE "Internal Validity" OR DE "Item Analysis (Test)" OR DE "Item Response Theory" OR DE "Measurement Invariance" OR DE "Measurement Models" OR DE "Multivariate Analysis" OR DE "Test Construction" OR DE "Test Reliability" OR DE "Test Sensitivity" OR DE "Test Specificity" OR DE "Test Validity" OR DE "Variability Measurement" OR DE "Factor Analysis" OR DE "Confirmatory Factor Analysis" OR DE "Exploratory Factor Analysis" OR DE "Factor Structure" OR DE "Item Analysis (Statistical)" OR DE "Statistical Rotation" OR DE "Item Analysis (Test)" OR DE "Differential Item Functioning" OR DE "Measurement Models" OR DE "Mixture Modeling" OR DE "Structural Equation Modeling" OR DE "Multivariate Analysis" OR DE "Factor Analysis" OR DE "Mixture Modeling" OR DE "Multiple Regression" OR DE "Path Analysis" OR DE "Principal Component Analysis" OR DE "Test Reliability" OR DE "Internal Consistency" OR DE "Interrater Reliability" OR DE "Split-Half Reliability" OR DE "Test-Retest Reliability" OR DE "Test Validity" OR DE "Clinical Validity" OR DE "Construct Validity" OR DE "Content Validity" OR DE "Criterion Validity" OR DE "Face Validity" OR DE "Factorial Validity" OR DE "Variability Measurement" OR DE "Analysis of Covariance" OR DE "Analysis of Variance" OR DE "Interaction Variance" OR DE "Standard Deviation" OR DE "Statistical Rotation" OR DE "Oblique Rotation" OR DE "Orthogonal Rotation" OR DE "Mixture Modeling" OR DE "Latent Class Analysis" OR DE "Latent Profile Analysis" OR DE "Structural Equation Modeling" OR DE "Latent Class Analysis" OR DE "Clinical Validity" OR DE "Discriminative Validity" OR DE "Test Responsiveness" OR DE "Construct Validity" OR DE "Convergent Validity" OR DE "Discriminant Validity" OR DE "Nomological Validity" OR DE "Criterion Validity" OR DE "Concurrent Validity" OR DE "Incremental Validity" OR DE "Predictive Validity"	327,271
S1	TI ("patient health questionnaire" or PHQ or "somatic symptom scale 8" or SSS-8) OR AB ("patient health questionnaire" or PHQ or "somatic symptom scale 8" or SSS-8) OR KW ("patient health questionnaire" or PHQ or "somatic symptom scale 8" or SSS-8)	3,125

© 2024 Hybelius J et al. *JAMA Network Open.*

Documentation of search strategies
University Library search consultation group

Date: 2 February 2024

Topic/research question: Sensitivity to change

Name of researcher(s): Erland Axelsson, Jonna Hybelius

Librarian(s): Emma-Lotta Säätelä

Databases:

4. Medline (Ovid)
5. Web of Science Core Collection (Clarivate)
6. Psycinfo (Ebsco)

Total number of hits:

- Before deduplication: 1,499
- After deduplication: 151 (+ 721 from previous search)

© 2024 Hybelius J et al. *JAMA Network Open.*

1. Medline

Interface: Ovid MEDLINE(R) ALL<br><br>Date of Search: 2 February 2024<br><br>Number of hits: 627<br><br>Comment: In Ovid, two or more words are automatically searched as phrases; i.e. no quotation marks are needed	Field labels<br><br>• exp/ = exploded MeSH term<br>• / = non exploded MeSH term<br>• .ti,ab,kf. = title, abstract and author keywords<br>• adjx = within x words, regardless of order<br>• * = truncation of word for alternate endings

Database(s): **Ovid MEDLINE(R) ALL** 1946 to February 01, 2024
Search Strategy:

#	Searches	Results
1	randomized controlled trial.pt.	607974
2	controlled clinical trial.pt.	95543
3	randomi?ed.ab.	756173
4	placebo.ab.	245414
5	clinical trials as topic.sh.	201704
6	randomly.ab.	426320
7	trial.ti.	302148
8	or/1-7	1625156
9	(systematic review or meta-analy*).ti.	330057
10	8 not 9	1528481
11	exp animals/ not humans.sh.	5193302
12	10 not 11	1402310
13	exp Patient Health Questionnaire/	979
14	("patient health questionnaire" or PHQ or "somatic symptom scale 8" or "8-item somatic symptom scale" or SSS-8 or somatization or "somatic symptom burden" or "somatic symptom severity" or "somatic symptom distress").ti,ab,kf.	19758
15	or/13-14	20046
16	12 and 15	2621
17	Behavioral Medicine/ or Psychosomatic Medicine/ or exp Somatoform Disorders/ or Medically Unexplained Symptoms/ or Burning Mouth Syndrome/ or exp Colonic Diseases, Functional/ or Dyspepsia/ or Fatigue Syndrome, Chronic/ or Fibromyalgia/ or Hyperventilation/ or Multiple Chemical Sensitivity/ or exp Pain/ or exp Premenstrual Syndrome/ or Temporomandibular Joint Dysfunction Syndrome/ or Tension-Type Headache/ or Whiplash Injuries/	546124
18	transdiagnostic.ti,ab,kf.	4653

19	(somatoform or somatiz* or "somatic symptom and related disorders" or "somatic symptom disorder" or SSD or "somatic symptom distress" or "persistent somatic symptoms" or "bodily distress" or "medically unexplained" or MUS or "functional somatic" or "burning mouth" or "irritable bowel syndrome" or IBS or dyspepsia or "chronic fatigue" or CFS or fibromyalgia or "globus syndrome" or hyperventilation or "multiple chemical" or (chronic adj3 pain) or (persistent adj3 pain) or "atypical chest pain" or "non-cardiac chest pain" or "premenstrual syndrome" or "pseudo-epileptic" or "temporomandibular joint dysfunction syndrome" or "tension headache" or whiplash or neurasthenia or conversion).ti,ab,kf.	468778
20	or/17-19	912211
21	16 and 20	763
22	limit 21 to yr="2000 -Current"	661
23	limit 22 to english language	627

© 2024 Hybelius J et al. *JAMA Network Open.*

2. Web of Science Core Collection

Interface: Clarivate Analytics<br><br>Editions = A&HCI , ESCI , SCI-EXPANDED , SSCI<br><br>Date of Search: 2 February 2024<br><br>Number of hits: 581	Field labels<br><br>• TS/Topic = title, abstract, author keywords and Keywords Plus<br>• NEAR/x = within x words, regardless of order<br>• * = truncation of word for alternate endings<br><br>Note: the *Exact search*-function was used for all the searches

#	Search Query	Results
1	TI=(”randomi$ed” OR ”randomi$ed” OR ”randomi$ation” OR ”randomi$ation” OR placebo* OR (random* AND (allocat* OR assign*)) OR (blind* AND (”single” OR ”double” OR ”treble” OR ”triple”))) OR AB=(”randomi$ed” OR ”randomi$ed” OR ”randomi$ation” OR ”randomi$ation” OR placebo* OR (random* AND (allocat* OR assign*)) OR (blind* AND (”single” OR ”double” OR ”treble” OR ”triple”)))	1154499
2	TI=(”systematic review” or ”meta-analy*”)	388018
3	#1 NOT #2	1057679
4	TS=("patient health questionnaire" OR PHQ OR "somatic symptom scale 8" OR "8-item somatic symptom scale" OR SSS-8 OR somatization OR "somatic symptom burden" OR "somatic symptom severity" OR "somatic symptom distress")	24103
5	#3 AND #4	2358
6	TS=(somatoform OR somatiz* OR "somatic symptom and related disorders" OR "somatic symptom disorder" OR SSD OR "somatic symptom distress" OR "persistent somatic symptoms" OR "bodily distress" OR "medically unexplained" OR MUS OR "functional somatic" OR "burning mouth" OR "irritable bowel syndrome" OR IBS OR dyspepsia OR "chronic fatigue" OR CFS OR fibromyalgia OR "globus syndrome" OR hyperventilation OR "multiple chemical" OR (chronic NEAR/2 pain) OR (persistent NEAR/2 pain) OR "atypical chest pain" OR "non-cardiac chest pain" OR "premenstrual syndrome" OR "pseudo-epileptic" OR "temporomandibular joint dysfunction syndrome" OR "tension headache" OR whiplash OR neurasthenia OR conversion OR transdiagnostic)	1057316
7	#5 AND #6 and Proceeding Paper or Meeting Abstract (Exclude – Document Types)<br>Timespan: 2000-01-01 to 2024-12-31	581

© 2024 Hybelius J et al. *JAMA Network Open.*

3. Psycinfo

Interface: EBSCO<br><br>Date of Search: 2 Februay 2024<br><br>Number of hits: 291	Field labels<br><br>• DE = subject heading<br>• TI = title<br>• AB = abstract<br>• KW = author keywords<br>• Nx = within x words, regardless of order<br>• * = truncation of word for alternate endings<br><br>Note: the *Apply equivalent subjects*-function was used for all the searches

#	Query	Results
S15	S10 AND S14<br>Limiters - Publication Date: 20000101-20240131<br>Source type: Academic journals	291
S14	S11 OR S12 OR S13	115,446
S13	TI (somatoform OR somatiz* OR "somatic symptom and related disorders" OR "somatic symptom disorder" OR SSD OR "somatic symptom distress" OR "persistent somatic symptoms" OR "bodily distress" OR "medically unexplained" OR MUS OR "functional somatic" OR "burning mouth" OR "irritable bowel syndrome" OR IBS OR dyspepsia OR "chronic fatigue" OR CFS OR fibromyalgia OR "globus syndrome" OR hyperventilation OR "multiple chemical" OR (chronic N2 pain) OR (persistent N2 pain) OR "atypical chest pain" OR "non-cardiac chest pain" OR "premenstrual syndrome" OR "pseudo-epileptic" OR "temporomandibular joint dysfunction syndrome" OR "tension headache" OR whiplash OR neurasthenia OR conversion) OR AB (somatoform OR somatiz* OR "somatic symptom and related disorders" OR "somatic symptom disorder" OR SSD OR "somatic symptom distress" OR "persistent somatic symptoms" OR "bodily distress" OR "medically unexplained" OR MUS OR "functional somatic" OR "burning mouth" OR "irritable bowel syndrome" OR IBS OR dyspepsia OR "chronic fatigue" OR CFS OR fibromyalgia OR "globus syndrome" OR hyperventilation OR "multiple chemical" OR (chronic N2 pain) OR (persistent N2 pain) OR "atypical chest pain" OR "non-cardiac chest pain" OR "premenstrual syndrome" OR "pseudo-epileptic" OR "temporomandibular joint dysfunction syndrome" OR "tension headache" OR whiplash OR neurasthenia OR conversion) OR KW (somatoform OR somatiz* OR "somatic symptom and related disorders" OR "somatic symptom disorder" OR SSD OR "somatic symptom distress" OR "persistent somatic symptoms" OR "bodily distress" OR "medically unexplained" OR MUS OR "functional somatic" OR "burning mouth" OR "irritable bowel syndrome" OR IBS OR dyspepsia OR "chronic fatigue" OR CFS OR fibromyalgia OR "globus syndrome" OR hyperventilation OR "multiple chemical" OR (chronic N2 pain) OR (persistent N2 pain) OR "atypical chest pain" OR "non-cardiac chest pain" OR "premenstrual syndrome" OR "pseudo-epileptic" OR "temporomandibular joint dysfunction syndrome" OR "tension headache" OR whiplash OR neurasthenia OR conversion)	61,842
S12	TI transdiagnostic OR AB transdiagnostic OR KW transdiagnostic	4,990
S11	DE ("Behavioral Medicine" OR "Psychosomatic Medicine" OR "Somatoform Disorders" OR "Conversion Disorder" OR "Factitious Disorders" OR "Neurasthenia" OR "Somatization Disorder" OR "Somatoform Pain Disorder" OR "Irritable Bowel Syndrome" OR Dyspepsia OR "Premenstrual Syndrome" OR Pain OR "Hyperventilation syndrome" OR "Chronic Fatigue Syndrome" OR "Muscle Contraction Headache" OR Whiplash)	70,807
S10	S8 AND S9	747

© 2024 Hybelius J et al. *JAMA Network Open.*

S9	TI ("patient health questionnaire" OR PHQ OR "somatic symptom scale 8" OR "somatic symptom scale-8" OR "8-item somatic symptom scale" OR SSS-8 OR somatization OR "somatic symptom burden" OR "somatic symptom severity" OR "somatic symptom distress") OR AB ("patient health questionnaire" OR PHQ OR "somatic symptom scale 8" OR "somatic symptom scale-8" OR "8-item somatic symptom scale" OR SSS-8 OR somatization OR "somatic symptom burden" OR "somatic symptom severity" OR "somatic symptom distress") OR KW ("patient health questionnaire" OR PHQ OR "somatic symptom scale 8" OR "somatic symptom scale-8" OR "8-item somatic symptom scale" OR SSS-8 OR somatization OR "somatic symptom burden" OR "somatic symptom severity" OR "somatic symptom distress")	9,198
S8	S6 NOT S7	205,932
S7	TI (”systematic review” OR meta-analy*).	56,925
S6	S1 OR S2 OR S3 OR S4 OR S5	216,888
S5	TI trial	49,565
S4	AB randomly	85,634
S3	AB placebo	44,159
S2	AB randomi?ed	101,511
S1	DE ”Randomized Controlled Trials”	1,048

eFigure 1. Studies Included per Publication Year

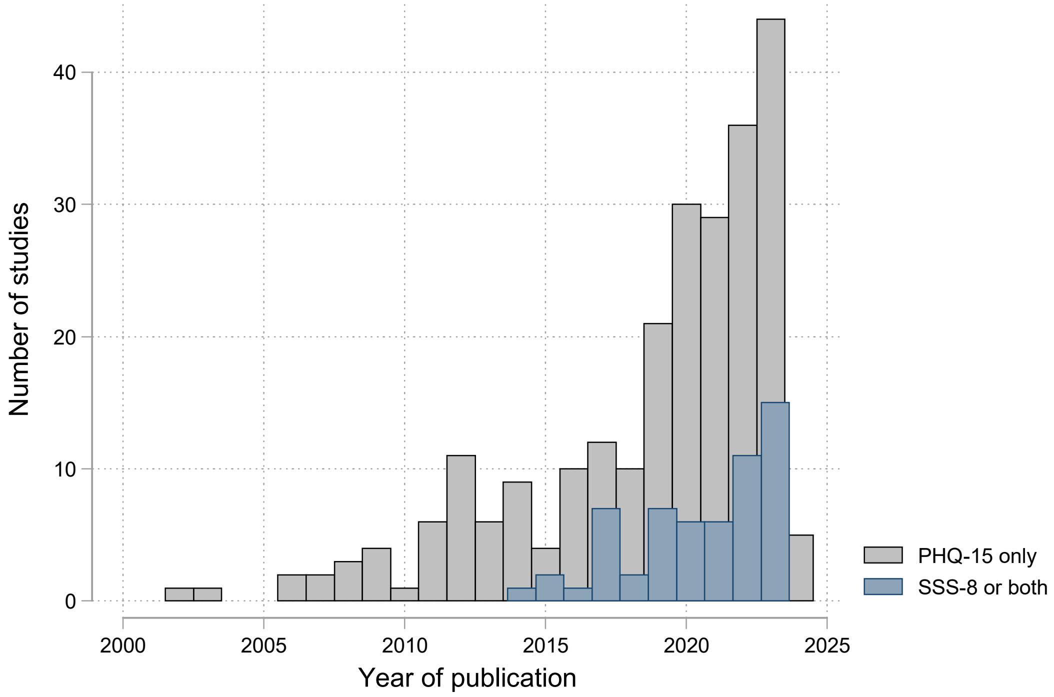

Abbreviations: PHQ-15, Patient Health Questionnaire 15; SSS-8, Somatic Symptom Scale 8.

© 2024 Hybelius J et al. *JAMA Network Open.*

eAppendix 3. Supplementary Methods

Details Pertaining to the Statistical Analysis

Separate analyses were conducted for the PHQ-15 and the SSS-8. Random-effects meta-analyses focused on internal consistency (Cronbach's α), mean scores, various correlations (Pearson's r), and standardized mean differences indicative of responsiveness (Hedges' g). The pooling of α was based on transformation and weighting in accordance with Hakstian-Whalen.[1] The pooling of r followed Hunter-Schmidt.[2] Standardized mean differences (g) were bias-corrected intention-to-treat between-groups estimates[3] calculated as the post-treatment mean difference, divided by the pooled standard deviation. The large-scale approximation of the sampling variance was used to enable the pooling of such standardized mean effects.[4] Absolute values for g of 0.20 are usually regarded small, 0.50 moderate, and 0.80 large.[5] Statistical heterogeneity was quantified in terms of the Q, τ^2, and I^2 statistics. The I^2 represents the proportion of the between-sample variance that is due to true study differences, as opposed to sampling error. Commonly, 25% is regarded low, 50% moderate, and 75% high,[6] though the I^2 also increases with original study sample sizes. In the meta-analysis of means and Cronbach's α, subgroup analyses were conducted for various clinical settings and patient groups. We developed the coding scheme for this purpose (eTable 3) iteratively during the tabulation phase, as based on a combination of clinical and theoretical interests, and what estimates proved to be available for tabulation. As can be seen in the overview provided in eTable 1, for all primary meta-analyses except those concerning Pearson's correlations, models were fitted using the restricted maximum likelihood method (REML). Several planned sensitivity analyses were conducted. Whenever relevant, we repeated analyses with English-language studies only. For the analysis of Pearson correlations, sensitivity analyses were also based on Fisher's r-to-Z transformation,[7] because even though the evidence is inconsistent, there have been arguments raised for less biased estimates when utilizing transformed correlations if the number of studies included in the meta-analysis is three or fewer.[2] Additional sensitivity analyses were also performed excluding studies deemed at high risk of bias due to the sampling strategy applied (applicable for the pooling of mean values) and excluding studies deemed at high risk of bias due to time dela (applicable for the pooling of r), see eTable 2 for further details on the assessment of risk of bias. For the reporting of AUROC, we applied common rules of thumb suggesting that an AUC corresponding to 0.51 to 0.69 indicates poor performance of a test, 0.70 to 0.79 implies a fair test, and 0.80 to 0.90 suggest good screening ability.[8]

© 2024 Hybelius J et al. *JAMA Network Open.*

eTable 1. Methods of Meta-Analysis, as Specified in the R Metafor Package

Outcome	Analysis	metafor:[a] arguments for escalc		metafor:[a] arguments for rma		Name of method	Reference
		measure	vtype	method	weights		
Cronbach's α	Primary	AHW	LS	REML	-	Approximation of Hakstian-Whalen	[1,9,10]
Mean	Primary	MN	LS	REML	-		[11]
Pearson's r	Primary	COR	AV	HS	ni	Approximation of Hunter-Schmidt	[2,11]
Pearson's r	Sensitivity	ZCOR	LS	REML	-	Fisher's *r*-to-*Z* transformation	[7]
Hedges' g	Primary	SMD	LS	REML	-		[3]

[a] For further information about the R metafor "escalc" and "rma" functions and their arguments, see https://www.metafor-project.org/

© 2024 Hybelius J et al. *JAMA Network Open.*

eTable 2. Coding Scheme for the Assessment of Risk of Bias, Loosely Based on Reilly et al (2015)

Criterion	High	Low	Unclear	Not applicable
Investigator bias	One or several of the authors listed as authors of the PHQ-15[13] or SSS-8[14] original publication.	None of the authors were involved in the original publications of the scales.		Studies included for norms and/or correlations only.
Sampling<br><br>Assessed for studies that aimed to recruit a general population sample, or reported estimates pertaining to a specific psychiatric disorder.	For general population samples:<br><br>- Missing data ≥ 20% and no compensatory modeling<br><br>For studies reporting estimates pertaining to a specific psychiatric disorders, at least one of the following:<br><br>- Not credible diagnostic assessment (e.g., cutoffs only)<br>- Patients eligible for treatment only (e.g., self-referred for participation in a RCT)<br>- Non-representative sample (e.g., too strict eligibility criteria given the setting)	For general population samples:<br><br>- Missing data ≤ 20% or compensatory modeling<br><br>For studies reporting estimates pertaining to a specific psychiatric disorders, all of the following:<br><br>- Credible diagnostic assessment (structured assessment by qualified clinician)<br>- Not only patients eligible for treatment (consecutive patients = low risk)<br>- Representative sample (given the to setting)	Not sufficiently reported.	Studies where none of the following (a/b/c) is true:<br><br>a. Study recruited a general population sample.<br><br>b. Study aimed to recruit participants based on a psychiatric disorder.<br><br>c. Study reports PHQ-15/SSS-8 estimates per psychiatric disorder.
Sample size	At least one of the following:<br><br>Factor analysis: n<300	At least one domain applicable, and all meeting the following:		Studies included for other domains only, such as norms, cut-offs,

© 2024 Hybelius J et al. *JAMA Network Open.*

Criterion	High	Low	Unclear	Not applicable
	Alpha: n<100<br>Clinical trials (sensitivity to change): n<126	Factor analysis: n≥300<br>Alpha: n≥100<br>Clinical trials (sensitivity to change): n≥126		correlations, MCID, test-retest reliability.
Time delay	Studies that report correlations with PHQ-15/SSS-8:<br><br>Comparator measures not administered the same day.	Studies that report correlations with PHQ-15/SSS-8:<br><br>Scales administered the same day.	Not sufficiently reported.	Studies that do not report correlations.

Abbreviations: PHQ-15, Patient Health Questionnaire 15; SSS-8, Somatic Symptom Scale 8; MUS, medically unexplained symptoms, FA, factor analysis; MCID, minimally clinical important difference.

© 2024 Hybelius J et al. *JAMA Network Open.*

eTable 3. Coding Scheme for Medical Conditions, Used for the Pooling of Means and Cronbach α

Category	Included conditions/diagnoses	Not included
Functional somatic symptoms and syndromes<br><br>*Umbrella term describing bodily complaints that currently cannot be fully explained by structural bodily pathology or organic disease.*[15]	Applicable for studies recruiting patients with functional somatic syndromes such as chronic pelvic pain, fibromyalgia, irritable bowel syndrome (IBS), non-cardiac chest pain, or medically unexplained symptoms (MUS).	Bodily complaints with a clear etiology, organic diseases, persistent physical symptoms where no assessment regarding etiology has been made, psychiatric disorders including somatic symptom disorder.
Somatoform disorders<br><br>*According to the ICD or DSM. Also including pathological health anxiety.*	Applicable for studies recruiting patients meeting criteria for a somatoform disorder such as somatization disorder, hypochondriasis, somatic symptom disorder, illness anxiety disorder, bodily distress disorder, or the presence of pathological health anxiety. Also includes the somatic symptom and related disorders of the DSM-5.	Functional somatic symptoms and syndromes.
Organic diseases<br><br>*Diseases largely explained by identifiable biological changes.*[16] *In this systematic review, often chronic diseases of long duration and generally slow progression.*	Applicable for studies recruiting patients based on specific diseases or conditions such as asthma, cancer, cardiovascular diseases, chronic obstructive pulmonary disease, Crohn's disease, diabetes, endometriosis, rare diseases, rheumatoid arthritis, traumatic brain injuries, ulcerative colitis.	Functional somatic symptoms and syndromes. Psychiatric disorders.
Persistent pain<br><br>*Persistent pain irrespective of etiology.*[17]	Applicable for studies recruiting patients based on the presence of pain deemed to be chronic, including persistent pain labelled by either (A) site of injury, or (B) type of injury, (C) chronic pain syndromes, (D) diseases commonly associated with chronic pain, and (E) unspecified chronic pain. Examples include chronic low back pain, chronic pelvic pain, complex regional pain syndrome, endometriosis, fibromyalgia, neuropathic pain, rheumatoid arthritis.	Acute pain, i.e., hours or days after an injury, or during the expected healing phase following injury.
Persistent physical symptoms<br><br>*Umbrella term describing distressing somatic symptoms irrespective of their etiology. The bodily complaints can be*	Applicable for studies that explicitly recruited participants based on the subjective experience of distress related to persistent physical symptoms, irrespective of the etiology of these bodily complaints.	Studies that recruited participants based on the presence of functional somatic symptoms or syndromes, or based on a medical or psychiatric diagnosis.

© 2024 Hybelius J et al. *JAMA Network Open.*

Category	Included conditions/diagnoses	Not included
attributable to symptoms related to clear medical pathophysiology or medically unexplained.[18]		
Depression<br><br>*According to the ICD or DSM*	Applicable for studies recruiting patients based on the presence of depression.	Studies recruiting mixed samples meeting criteria for either - or both - depression and/or anxiety disorders.
Anxiety disorders<br><br>*According to the ICD or DSM*	Applicable for studies recruiting patients based on the presence of an anxiety disorder, including mixed samples (i.e., any anxiety disorders included).	Studies recruiting mixed samples meeting criteria for either - or both - depression and/or anxiety disorders.
Eating disorders<br><br>*According to the ICD or DSM*	Applicable for studies recruiting patients based on the presence of an eating disorder, including mixed samples (i.e., any eating disorder included).	Studies recruiting mixed samples meeting criteria for either - or a combination of - depression, anxiety disorders, and/or eating disorders.
PTSD<br><br>*According to the ICD or DSM*	Applicable for studies recruiting patients based on the presence of post-traumatic stress disorder (PTSD).	Studies recruiting mixed samples meeting criteria for either - or both - depression and/or another disorder, including PTSD.

Abbreviations: ICD, International Classification of Diseases; DSM, Diagnostic and Statistical Manual of Mental Disorders.

© 2024 Hybelius J et al. *JAMA Network Open.*

eTable 4. Study Characteristics

First author	Year	Country	Sample	N	Age M	% Women	Language	fa[a]	txo	ico	cor	mns	cut	mci	trt	stc	Ref
Abasi	2022	Iran	Other Medical Care	238	36	75	Persian	no	no	no	yes	yes	no	no	no	no	19
Adewuya	2018	Nigeria	General Population	11246	37	58	Mixed	no	no	no	no	yes	no	no	no	no	20
Aguirre Cárdenas	2021	Chile	Other Medical Care	407	49	100	Spanish	no	no	yes	no	yes	no	no	no	no	21
Ahmed	2022	Pakistan	Other Medical Care	159	48	39	Unclear	no	no	yes	no	yes	no	no	no	no	22
Ak	2022	Turkey	Other Medical Care	60	-	98	Turkish	no	no	no	no	yes	no	no	no	no	23
AlHadi	2017	Saudi Arabia	Students	731	21	52	Arabic	no	no	yes	no	no	no	no	no	no	24
Almwled	2022	Saudi Arabia	Other Medical Care	207	34	54	Arabic	no	no	no	no	yes	no	no	no	no	25
Alosaimi	2019	Saudi Arabia	Mixed	209	56	56	Arabic	no	no	no	no	yes	no	no	no	no	26
Altamura	2022	Italy	Other Medical Care	41	71	17	Italian	no	no	no	no	yes	no	no	no	no	27
Althaus	2016	Germany	Other Medical Care	2419	37	54	German	no	no	no	no	yes	no	no	no	no	28
Andreasson	2020	Australia	Other Medical Care	158	-	84	English	no	no	no	no	yes	no	no	no	no	29
Arsenakis	2021	Mixed	Other/Convenience	749	-	68	English	no	no	no	yes	no	no	no	no	no	30
Barends	2020	Netherlands	Mixed	325	47	75	Unclear	no	no	no	no	yes	no	no	no	no	31
Baum	2022	Germany	Other Medical Care	227	50	64	German	no	no	no	no	yes	no	no	no	no	32
Becker	2023	Germany	Psychosomatic Clinic	205	39	57	German	no	no	no	no	yes	no	no	no	no	33
Beiner	2023	Germany	Other Medical Care	80	49	89	German	no	no	no	no	yes	no	no	no	no	34
Berens	2019	Germany	Other Medical Care	254	36	63	German	no	no	no	no	yes	no	no	no	no	35
Berens	2022	Germany	Other Medical Care	281	41	63	German	no	no	no	no	yes	no	no	no	no	36
Berens	2022	Sweden	Other Medical Care	114	34	69	Swedish	no	no	no	no	yes	no	no	no	no	36
Berens	2022	USA	Other Medical Care	122	55	78	English	no	no	no	no	yes	no	no	no	no	36
Black	2020	UK	Other/Convenience	1097	48	85	English	no	no	no	no	yes	no	no	no	no	37
Boecking	2021	Germany	Other Medical Care	352	53	54	German	no	no	yes	yes	yes	no	no	no	no	38
Borho	2021	Germany	Other/Convenience	116	37	31	Arabic	no	no	no	yes	no	no	no	no	no	39
Bornstein	2023	USA	Primary Care, General	100	39	74	English	no	no	yes	no	yes	no	no	no	no	40
Brand	2022	Germany	Other/Convenience	619	44	79	German	no	no	no	yes	no	no	no	no	no	41
Brettschneider	2013	Germany	Primary Care, General	294	49	75	German	no	no	no	no	yes	no	no	no	no	42

© 2024 Hybelius J et al. *JAMA Network Open.*

First author	Year	Country	Sample	N	Age M	% Women	Language	fa[a]	txo	ico	cor	mns	cut	mci	trt	stc	Ref
Brown	2012	UK	Other Medical Care	55	39	49	English	no	no	yes	no	yes	no	no	no	no	43
Brünahl	2021	Germany	Other Medical Care	60	49	33	German	no	no	no	no	yes	no	no	no	no	44
Cano-Garcia	2020	Spain	Primary Care, Mental H.	1255	44	77	Spanish	yes	no	yes	no	yes	no	no	yes	no	45
Cao	2020	China	Other Medical Care	697	43	61	Chinese	no	no	yes	yes	yes	yes	no	no	no	46
Chaabouni	2024	Netherlands	Primary Care, General	113	58	67	Dutch	no	no	no	no	yes	no	no	no	no	47
Chalder	2023	UK	Other Medical Care	324	43	83	English	no	no	no	no	yes	no	no	no	no	48
Chen	2020	China	Psychiatry	33	41	61	Unclear	no	no	no	no	yes	no	no	no	no	49
Cho	2015	South Korea	Other Medical Care	101	62	0	Korean	no	no	no	no	yes	no	no	no	no	50
Choi	2022	South Korea	Other/Convenience	1191	34	81	Korean	no	no	no	yes	no	no	no	no	no	51
Ciaramella	2023	Italy	Psychosomatic Clinic	123	33	71	Italian	no	no	no	no	yes	no	no	no	no	52
Clarke	2008	Australia	Primary Care, General	10507	49	69	English	no	no	no	yes	yes	no	no	no	no	53
Cohen	2021	Israel	Other/Convenience	117	40	94	Hebrew	no	no	yes	no	yes	no	no	no	no	54
Cooper	2017	Canada	Primary Care, General	96	48	73	Unclear	no	no	no	no	yes	no	no	no	no	55
Corpas	2021	Spain	Primary Care, Mental H.	102	39	88	Spanish	no	no	no	no	yes	no	no	no	no	56
Corpas	2022	Spain	Primary Care, Mental H.	105	40	69	Spanish	no	no	no	no	yes	no	no	no	no	57
Dadfar	2018	Iran	Psychiatry	130	31	73	Persian	no	no	no	yes	yes	no	no	no	no	58
Dadfar	2018	Iran	Psychiatry	116	35	82	Persian	no	no	yes	no	no	no	no	no	no	59
Dadfar	2020	Iran	Psychiatry	111	31	80	Persian	no	no	yes	no	yes	no	no	no	no	60
Dadfar	2020	Iran	Mixed	349	41	65	Persian	yes	no	yes	yes	no	no	no	no	no	61
Dagnall	2023	UK	Other/Convenience	1665	54	51	English	yes	no	no	yes	no	no	no	no	no	62
Daisuke	2022	Japan	Other Medical Care	16	51	100	Japanese	no	no	no	no	yes	no	no	no	no	63
Denovan	2019	UK	Other/Convenience	170	30	62	English	yes	no	no	yes	no	no	no	no	no	64
Depping	2021	Germany	Other Medical Care	80	46	66	German	no	no	no	no	yes	no	no	no	no	65
Dinkel	2021	Germany	Other Medical Care	327	59	35	German	no	no	yes	no	yes	no	no	no	no	66
Dischinger	2019	USA	Other/Convenience	194	46	84	English	no	no	yes	no	no	no	no	no	no	67
Dreher	2017	Germany	Psychiatry	219	45	84	Mixed	yes	no	yes	no	yes	no	no	no	no	68
Dsilva	2023	Canada	Mixed	79	45	92	English	no	no	no	no	yes	no	no	no	no	69
Eakman	2019	USA	Other/Convenience	162	31	17	English	no	no	no	yes	no	no	no	no	no	70

© 2024 Hybelius J et al. *JAMA Network Open.*

First author	Year	Country	Sample	N	Age M	% Women	Language	fa[a]	txo	ico	cor	mns	cut	mci	trt	stc	Ref
Easton	2017	Palest. terr.	Other/Convenience	234	38	70	Arabic	no	no	no	yes	no	no	no	no	no	71
Fahrni	2014	Switzerland	Other Medical Care	71	40	51	German	no	no	no	yes	yes	no	no	no	no	72
Fairbrass	2022	UK	Other Medical Care	125	49	58	English	no	no	no	no	yes	no	no	no	no	73
Falahatdoost	2020	Iran	General Population	229	27	61	Persian	no	no	no	no	yes	no	no	no	no	74
Fallon	2021	USA	Other Medical Care	193	40	56	English	no	no	no	no	yes	no	no	no	no	75
Fleischer	2022	Germany	Other Medical Care	128	45	67	German	no	no	no	no	yes	no	no	no	no	76
Flores-Arriaga	2023	Spain	General Population	2072	46	50	Spanish	no	no	no	no	yes	no	no	no	no	77
Forney	2023	USA	Other/Convenience	380	20	84	English	no	no	yes	no	yes	no	no	no	no	78
Fowler	2014	USA	Psychiatry	592	36	51	English	no	no	yes	no	yes	no	no	no	no	79
Fowler	2022	USA	Psychiatry	804	35	48	English	no	no	yes	no	yes	no	no	no	no	80
Fresán	2023	Mexico	Other Medical Care	135	42	90	Spanish	no	no	no	no	yes	no	no	no	no	81
Freyler	2019	Hungary	Psychiatry	66	44	77	Hungarian	no	no	yes	yes	yes	no	no	no	no	82
Fu	2023	China	Other Medical Care	1324	49	59	Chinese	no	no	no	yes	no	no	no	no	no	83
Fujii	2018	Japan	Other/Convenience	3100	45	48	Japanese	no	no	no	yes	yes	no	no	no	no	84
Ghapanch	2022	Iran	Psychiatry	122	35	74	Persian	yes	no	yes	yes	yes	no	no	yes	no	85
Gica	2020	Turkey	Other/Convenience	533	35	57	Turkish	no	no	yes	no	no	no	no	no	no	86
Gierk	2014	Germany	General Population	2510	49	53	German	yes	no	yes	yes	yes	yes	no	no	no	14
Gierk	2015	Germany	Psychosomatic Clinic	131	42	66	German	no	no	yes	yes	yes	no	no	no	no	87
Gierk	2017	Germany	Psychiatry	55	44	78	German	no	no	yes	no	yes	no	yes	no	no	88
Gitzen	2024	Germany	Other Medical Care	62	41	69	German	no	no	no	no	yes	no	no	no	no	89
Goodarzi	2020	Iran	Other/Convenience	281	24	65	Persian	yes	no	no	yes	no	no	no	no	no	90
Gould	2019	USA	Other/Convenience	40	69	60	English	no	no	yes	yes	yes	no	no	no	no	91
Gracie	2016	UK	Other Medical Care	356	49	58	English	no	no	no	no	yes	no	no	no	no	92
Gray	2020	UK	Other Medical Care	430	42	69	English	no	no	yes	yes	no	no	no	no	no	93
Grover	2019	India	Psychiatry	60	66	55	Hindi	yes	no	no	no	no	no	no	no	no	94
Grover	2023	India	Other Medical Care	79	30	89	Unclear	no	no	no	yes	yes	no	no	no	no	95
Habtamu	2022	Ethiopia	Primary Care, General	587	36	49	Amharic	yes	no	yes	yes	no	no	no	no	no	96
Hackenberg	2023	Germany	General Population	8539	61	49	German	no	no	no	yes	yes	no	no	no	no	97

© 2024 Hybelius J et al. *JAMA Network Open.*

First author	Year	Country	Sample	N	Age M	% Women	Language	fa[a]	txo	ico	cor	mns	cut	mci	trt	stc	Ref
Haggarty	2016	Canada	Primary Care, Mental H.	508	38	70	Unclear	no	no	no	no	yes	no	no	no	no	98
Hamdan	2020	Israel	Other/Convenience	180	42	78	Hebrew	no	no	no	yes	no	no	no	no	no	99
Han	2009	South Korea	Psychiatry	57	38	56	Korean	no	no	yes	yes	no	no	no	yes	no	100
Hasegawa	2021	Japan	Other Medical Care	21	79	67	Japanese	no	no	no	no	yes	no	no	no	no	101
Hashimoto	2023	Japan	Psychosomatic Clinic	120	48	64	Japanese	no	no	no	no	yes	no	no	no	no	102
Hausteiner	2009	Germany	Other Medical Care	48	43	81	German	no	no	no	no	yes	no	no	no	no	103
Hennemann	2022	Mixed	Students	156	25	83	German	no	no	yes	no	no	no	no	no	no	104
Hennemann	2023	USA	Other/Convenience	313	24	79	Mixed	no	no	no	no	yes	no	no	no	no	105
Herzog	2015	Germany	Psychosomatic Clinic	262	43	60	German	no	no	no	no	yes	yes	no	no	no	106
Hietaharju	2021	Finland	Other Medical Care	197	43	80	Finnish	no	no	yes	yes	yes	no	no	no	no	107
Hijne	2024	Portugal	Other/Convenience	686	45	93	Mixed	no	no	no	no	yes	no	no	no	no	108
Hilbert	2015	Germany	Other/Convenience	1158	54	46	German	no	no	no	yes	no	no	no	no	no	109
Hinz	2023	Germany	General Population	5355	57		German	no	no	no	yes	no	no	no	no	no	110
Horn	2023	France	Other Medical Care	220	53	45	French	no	no	yes	no	no	no	no	no	no	111
Huth	2023	Germany	Other Medical Care	63	47	81	German	no	no	yes	no	yes	no	no	no	no	112
Hwang	2021	South Korea	Mixed	203	39	42	Korean	no	no	yes	no	yes	no	no	no	no	113
Hybelius	2022	Sweden	Other/Convenience	33	46	67	Swedish	no	no	no	no	yes	no	no	no	yes	114
Hyphantis	2014	Greece	Other Medical Care	303	70	50	Greek	no	no	yes	no	no	no	no	no	no	115
Häuser	2009	Germany	General Population	2524	49	52	German	no	no	no	no	yes	no	no	no	no	116
Häuser	2015	Germany	Other Medical Care	156	50	89	German	no	no	no	no	yes	no	no	no	no	117
Häuser	2021	Germany	General Population	2531	49	51	German	no	no	no	no	yes	no	no	no	no	118
Inan-Budak	2023	Turkey	Other/Convenience	204	36	100	Turkish	no	no	no	yes	no	no	no	no	no	119
Interian	2006	USA	Primary Care, Mental H.	117	39	92	Spanish	no	no	yes	yes	yes	no	no	no	no	120
Interian	2006	USA	Primary Care, Mental H.	55	43	78	English	no	no	yes	yes	yes	no	no	no	no	120
Jasper	2012	Germany	Students	782	23	53	German	no	yes	no	no	no	no	no	no	no	121
Jasper	2012	Germany	Students	2577	22	49	German	no	yes	no	no	no	no	no	no	no	121
Jehangir	2020	USA	Other Medical Care	432	43	83	English	no	no	no	no	yes	no	no	no	no	122
Jeon	2017	South Korea	Psychiatry	891	44	58	Korean	no	no	no	yes	no	no	no	no	no	123

© 2024 Hybelius J et al. *JAMA Network Open.*

First author	Year	Country	Sample	N	Age M	% Women	Language	fa[a]	txo	ico	cor	mns	cut	mci	trt	stc	Ref
Johnson	2019	Australia	Other Medical Care	24	40	38	English	no	no	no	no	yes	no	no	no	no	[124]
Johnson	2021	USA	Other/Convenience	164	58	64	English	no	no	no	no	yes	no	no	no	no	[125]
Jongsma	2023	Canada	Psychiatry	13	44	73	English	no	no	no	yes	no	no	no	no	no	[126]
Jungmann	2024	Germany	Psychiatry	120	37	68	German	no	no	no	no	yes	no	no	no	no	[127]
Kalibatseva	2018	USA	Students	519	20	63	English	no	no	no	yes	no	no	no	no	no	[128]
Kalkbrenner	2023	USA	General Population	821	47	52	English	no	no	yes	yes	yes	no	no	no	no	[129]
Kamata	2023	Japan	General Population	30130	48	51	Japanese	no	no	no	no	yes	no	no	no	no	[130]
Kamimura	2014	USA	Primary Care, General	186	46	61	English	no	no	no	no	yes	no	no	no	no	[131]
Kamimura	2020	USA	Primary Care, General	506	46	69	English	no	no	no	no	yes	no	no	no	no	[132]
Kane	2018	UK	Other Medical Care	151	68	75	English	no	no	no	no	yes	no	no	no	no	[133]
Kara	2018	UK	Other Medical Care	61	54	52	English	no	no	no	no	yes	no	no	no	no	[134]
Kawasaki	2020	Japan	Other Medical Care	121	54	79	Japanese	no	no	no	no	yes	no	no	no	no	[135]
Kealy	2018	Canada	Other/Convenience	248	26	74	English	no	no	no	yes	no	no	no	no	no	[136]
Khaustova	2022	Ukraine	Other Medical Care	193	37	60	Ukrainian	no	no	no	no	yes	no	no	no	no	[137]
Kim	2023	South Korea	Other/Convenience	1060	46	100	Korean	no	no	no	yes	no	no	no	no	no	[138]
Kliem	2014	Germany	General Population	4903	48	52	German	yes	yes	no	no	no	no	no	no	no	[139]
Klotz	2020	Germany	Other Medical Care	187	49	57	German	no	no	no	no	yes	no	no	no	no	[140]
Knopp	2024	Austria	Psychiatry	135	49		German	no	no	yes	no	yes	no	no	no	no	[141]
Kobel	2020	Germany	Psychiatry	263	39	68	German	no	no	yes	no	yes	no	no	no	no	[142]
Kocalevent	2013	Germany	General Population	5031	49	54	German	no	no	yes	yes	yes	no	no	no	no	[143]
Koh	2014	South Korea	Other Medical Care	80	50	0	Korean	no	no	no	yes	yes	no	no	no	no	[144]
Körber	2011	Germany	Primary Care, General	308	47	71	German	no	no	no	yes	yes	yes	no	no	no	[145]
Kroenke	2002	USA	Mixed	6000	39	83	English	no	no	yes	no	no	yes	no	no	no	[13]
Kube	2023	Germany	Psychiatry	92	44	75	German	no	no	no	no	yes	no	no	no	no	[146]
Kuby	2019	Germany	Primary Care, General	1645	51	61	German	no	no	yes	no	no	no	no	no	no	[147]
Köteles	2011	Hungary	Mixed	308	35	62	Hungarian	no	no	yes	yes	yes	no	no	no	no	[148]
Laferton	2017	Germany	General Population	250	49	65	German	no	no	yes	no	yes	yes	no	no	no	[149]
Lahmann	2017	Germany	Mixed	81	47	78	German	no	no	yes	no	no	no	no	no	no	[150]

© 2024 Hybelius J et al. *JAMA Network Open.*

First author	Year	Country	Sample	N	Age M	% Women	Language	fa[a]	txo	ico	cor	mns	cut	mci	trt	stc	Ref
Langhorst	2023	Germany	Other/Convenience	41	55	95	German	no	no	no	no	yes	no	no	no	no	151
Lanzara	2020	Italy	Other Medical Care	134	50	55	Italian	no	no	yes	no	no	no	no	no	no	152
Le	2020	Canada	Other/Convenience	186	40	82	Unclear	no	no	no	yes	no	no	no	no	no	153
Lee	2011	China	General Population	3014		53	Chinese	yes	no	yes	yes	no	no	no	yes	no	154
Lee	2020	South Korea	Psychiatry	144	44	47	Korean	no	no	no	no	yes	no	no	no	no	155
Lee	2021	South Korea	Other/Convenience	118	33	71	Korean	no	no	no	yes	yes	no	no	no	no	156
Leithner	2009	Austria	Other Medical Care	300	31	100	German	no	no	no	yes	yes	no	no	no	no	157
Leonhart	2018	Mixed	Mixed	4302	46	60	Mixed	yes	no	no	no	no	no	no	no	no	158
Lewis	2017	UK	Primary Care, Mental H.	695	77	58	English	no	no	no	no	yes	no	no	no	no	159
Li	2019	China	Other Medical Care	3044	28	69	Chinese	no	no	yes	no	no	no	no	no	no	160
Li	2021	China	General Population	500	39	66	Chinese	no	no	no	no	yes	no	no	no	no	161
Li	2022	China	Other Medical Care	264	51	100	Chinese	no	no	yes	no	yes	no	no	no	no	162
Li	2023	China	Mixed	273	51		Chinese	no	no	no	no	yes	no	no	no	no	163
Li	2023	China	Other Medical Care	871	42	65	Chinese	no	no	no	no	no	yes	no	no	no	164
Li	2023	China	Other Medical Care	90	48	61	Chinese	no	no	no	no	yes	no	no	no	no	165
Li	2023	China	Psychiatry	177	38	66	Chinese	no	no	yes	no	yes	no	no	no	no	166
Liao	2016	Taiwan	Mixed	471	45	63	Chinese	yes	no	yes	yes	yes	yes	no	yes	no	167
Liao	2021	Taiwan	Mixed	405	44	66	Chinese	no	no	yes	yes	yes	no	no	no	no	168
Limburg	2021	Germany	Other Medical Care	159	54	62	German	no	no	no	no	yes	no	no	no	no	169
Lyoo	2014	South Korea	Other/Convenience	350	24	68	Korean	yes	no	yes	yes	no	no	no	yes	no	170
Löwe	2008	USA	Primary Care, General	2091	47	66	English	no	no	no	yes	yes	no	no	no	no	171
Löwe	2011	USA	Primary Care, General	965	47	69	English	no	no	yes	no	no	no	no	no	no	172
Ma	2019	China	Psychiatry	323	34	48	Chinese	no	no	yes	yes	yes	no	no	no	no	173
Ma	2022	China	Psychiatry	939	22	75	Chinese	no	no	yes	no	yes	no	no	no	no	174
Ma	2023	Taiwan	Psychiatry	30	38	70	Chinese	no	no	no	no	yes	no	no	no	no	175
Manning	2022	USA	Other/Convenience	166	21	88	English	no	no	no	yes	no	no	no	no	no	176
Manuel	2023	Germany	Other Medical Care	914	54	68	German	no	no	no	yes	yes	no	no	no	no	177
Marks	2016	UK	Other Medical Care	77	51	55	English	no	no	no	no	yes	no	no	no	no	178

© 2024 Hybelius J et al. *JAMA Network Open.*

First author	Year	Country	Sample	N	Age M	% Women	Language	fa[a]	txo	ico	cor	mns	cut	mci	trt	stc	Ref
Martela	2020	USA	Other/Convenience	444	38	44	English	no	no	no	yes	no	no	no	no	no	179
Martinez	2021	USA	Mixed	98	41	91	English	no	no	no	no	yes	no	no	no	no	180
Matsudaira	2017	Japan	Other/Convenience	52353	-	50	Japanese	no	no	yes	yes	no	no	no	no	no	181
Maulina	2022	Mixed	Other/Convenience	906	20	53	Mixed	no	no	no	yes	no	no	no	no	no	182
McAndrew	2017	USA	Other/Convenience	336	30	14	English	no	no	yes	no	no	no	no	no	no	183
McWhirter	2022	UK	Mixed	31	63	58	English	no	no	no	no	yes	no	no	no	no	184
Menzies	2019	Australia	Psychiatry	200	34	63	English	no	no	yes	no	no	no	no	no	no	185
Menzies	2021	Australia	Psychiatry	128	35	68	English	no	no	yes	no	yes	no	no	no	no	186
Mewes	2008	Germany	General Population	2510	48	55	German	no	no	no	no	yes	no	no	no	no	187
Mitra	2023	India	Other Medical Care	108	44	50	Hindi	no	no	no	yes	no	no	no	no	no	188
Mohaghegh	2021	Iran	Other Medical Care	128	37	60	Persian	no	no	yes	no	no	no	no	no	no	189
Moritz	2019	Germany	Mixed	163	28	75	German	no	no	no	no	yes	no	no	no	no	190
Mourad	2018	Sweden	Other Medical Care	552	64	51	Swedish	no	no	yes	no	yes	no	no	no	no	191
Muller	2019	Germany	Other Medical Care	106	32	9	German	no	no	no	no	yes	no	no	no	no	192
Mund	2023	Germany	Other Medical Care	167	45	65	German	no	no	no	no	yes	no	no	no	no	193
Mutepfa	2021	Botswana	Other/Convenience	378	72	75	Mixed	no	no	no	yes	no	no	no	no	no	194
Müller	2022	Germany	Psychosomatic Clinic	861	34	64	German	no	no	yes	yes	no	no	no	no	no	195
Nacak	2021	Germany	Mixed	130	46	72	German	no	no	no	yes	no	no	no	no	no	196
Nasstasia	2019	Australia	Other/Convenience	68	21	78	English	no	no	yes	yes	yes	no	no	no	no	197
Nehme	2023	Lebanon	Other/Convenience	403	33	66	Arabic	no	no	no	yes	no	no	no	no	no	198
Newby	2017	Australia	Other/Convenience	118	31	86	English	no	no	no	no	yes	no	no	no	no	199
Newby	2018	Australia	Other/Convenience	86	30	87	English	no	no	yes	no	yes	no	no	no	no	200
Nickel	2022	USA	Other/Convenience	50	36	70	English	no	no	no	no	yes	no	no	no	no	201
Nikendei	2019	Germany	Other Medical Care	45	58	62	German	no	no	yes	no	yes	no	no	no	no	202
Nishiyama	2022	Japan	Other Medical Care	1975	53	63	Japanese	no	no	no	no	yes	no	no	no	no	203
Nordin	2013	Sweden	General Population	3406	51	56	Swedish	yes	no	yes	yes	yes	no	no	no	no	204
North	2019	USA	Other Medical Care	50	47	52	English	no	no	no	no	yes	yes	no	no	no	205
Orme	2021	USA	Psychiatry	2702	35	48	English	no	no	no	no	yes	no	no	no	no	206

© 2024 Hybelius J et al. *JAMA Network Open.*

First author	Year	Country	Sample	N	Age M	% Women	Language	fa[a]	txo	ico	cor	mns	cut	mci	trt	stc	Ref
Ostrc	2022	Slovenia	Other Medical Care	177	36	77	Slovenian	no	no	no	no	yes	no	no	no	no	207
Paredes-Echeverri	2022	USA	Other Medical Care	78	43	68	English	no	no	no	no	yes	no	no	no	no	208
Park	2023	South Korea	Unclear	167	26	68	Korean	no	no	no	yes	no	no	no	no	no	209
Parkes	2019	UK	Other Medical Care	30	49	67	English	no	no	no	no	yes	no	no	no	no	210
Patel	2020	UK	Primary Care, General	157	47	81	English	no	no	no	no	yes	no	no	no	no	211
Patel	2022	India	Other/Convenience	111	34	100	Hindi	no	no	no	yes	no	no	no	no	no	212
Perros	2023	Mixed	Other/Convenience	3485	-	95	Mixed	no	no	no	no	yes	no	no	no	no	213
Persoons	2003	Belgium	Other Medical Care	268	48	66	Dutch	no	no	no	no	yes	no	no	no	no	214
Petrelis	2022	Greece	Other Medical Care	138	43	57	Greek	yes	no	yes	yes	yes	no	no	yes	no	215
Petrik	2021	USA	Other Medical Care	110	38	59	English	no	no	yes	no	yes	no	no	no	no	216
Philipps	2019	Germany	Psychosomatic Clinic	66	40	83	German	no	no	yes	no	yes	no	no	no	no	217
Pick	2020	UK	Other Medical Care	21	-	86	English	no	no	no	no	yes	no	no	no	no	218
Pick	2023	UK	Other/Convenience	14	39	71	English	no	no	no	no	yes	no	no	no	no	219
Pollo	2022	Brazil	Other Medical Care	300	52	53	Portuguese	yes	no	yes	no	no	no	no	no	no	220
Rady	2021	Egypt	Other Medical Care	196	-	90	Arabic	no	no	no	no	yes	no	no	no	no	221
Reddy	2019	India	Psychiatry	50	49	-	Unclear	no	no	no	no	yes	no	no	no	no	222
Reuber	2007	UK	Other Medical Care	91	-	-	English	no	no	no	no	yes	no	no	no	no	223
Reutimann	2023	Germany	Psychiatry	495	39	68	German	no	no	yes	no	no	no	no	no	no	224
Rief	2012	Germany	General Population	2485	49	52	German	no	no	yes	no	yes	no	no	no	no	225
Roca	2013	Spain	Primary Care, Mental H.	10257	49	68	Spanish	no	no	no	no	yes	no	no	no	no	226
Rohricht	2019	Germany	Psychosomatic Clinic	22	50	73	German	no	no	no	no	yes	no	no	no	no	227
Rometsch	2023	Germany	Psychosomatic Clinic	95	46	78	German	no	no	no	yes	no	no	no	no	no	228
Roohafza	2023	Iran	Other Medical Care	360	56	56	Persian	no	no	no	no	yes	no	no	no	no	229
Ros Montalbán	2010	Spain	Psychiatry	3362	46	66	Spanish	no	no	yes	yes	yes	no	no	no	no	230
Rosales	2020	USA	Other Medical Care	143	39	83	English	no	no	no	no	yes	no	no	no	no	231
Sammarra	2022	Italy	Other Medical Care	51	41	57	Italian	no	no	no	yes	no	no	no	no	no	232
Sayuk	2022	USA	Other Medical Care	29	47	79	English	no	no	no	no	yes	no	no	no	no	233
Scarella	2016	USA	Other/Convenience	186	40	56	English	no	no	no	yes	no	no	no	no	no	234

© 2024 Hybelius J et al. *JAMA Network Open.*

First author	Year	Country	Sample	N	Age M	% Women	Language	fa[a]	txo	ico	cor	mns	cut	mci	trt	stc	Ref
Schaefert	2012	China	Other Medical Care	281	42	64	Chinese	no	no	yes	no	yes	no	no	no	no	235
Schaeuffele	2022	Germany	Other/Convenience	12	37	68	German	no	no	no	no	yes	no	no	no	no	236
Schlechter	2023	Germany	Other/Convenience	369	28	46	Arabic	yes	no	no	yes	no	no	no	no	no	237
Schmalbach	2020	Germany	General Population	2386	48	53	German	no	no	no	yes	yes	no	no	no	no	238
Schneider	2011	Germany	Primary Care, General	1005	49	59	German	no	no	no	no	yes	no	no	no	no	239
Schneider	2017	Germany	Other/Convenience	71	47	75	German	no	no	no	no	yes	no	no	no	no	240
Shahini	2021	Iran	Mixed	135	34	64	Persian	no	no	no	yes	no	no	no	no	no	241
Shin	2012	South Korea	Psychiatry	194	49	67	Korean	no	no	no	yes	yes	no	no	no	no	242
Sikharulidze	2022	Georgia	Other Medical Care	83	45	59	Georgian	no	no	yes	yes	yes	no	no	no	no	243
Singh	2020	USA	Other Medical Care	48	49	75	English	no	no	no	no	yes	no	no	no	no	244
Sitnikova	2019	Netherlands	Primary Care, Mental H.	198	52	75	Dutch	no	no	no	no	no	no	no	no	no	245
Sogutlu	2011	USA	Mixed	230	34	62	English	no	no	no	no	yes	no	no	no	no	246
Sood	2023	India	Psychiatry	93	31	41	Hindi	no	no	no	no	yes	no	no	no	no	247
Soucy	2017	Canada	Other/Convenience	116	41	70	English	no	no	no	no	yes	no	no	no	no	248
Speck	2022	Germany	Other/Convenience	410	28	78	German	no	no	no	yes	no	no	no	no	no	249
Starcevic	2020	South Korea	Psychiatry	100	21	6	Korean	no	no	no	no	yes	no	no	no	no	250
Stauder	2021	Hungary	Other/Convenience	5020	51	59	Hungarian	yes	no	yes	yes	no	no	no	no	no	251
Stegenga	2012	Netherlands	Primary Care, General	1318	51	63	Dutch	no	no	no	no	yes	no	no	no	no	252
Stein	2023	Mixed	Other/Convenience	365	25	75	Arabic	no	no	yes	no	yes	no	no	no	no	253
Sun	2020	China	Psychiatry	73	-	59	Chinese	no	no	no	no	yes	no	no	no	no	254
Sweetman	2022	UK	Other/Convenience	1980	36	73	English	no	no	no	yes	no	no	no	no	no	255
Taenzer	2023	Austria	Other/Convenience	41	-	71	German	no	no	no	yes	no	no	no	no	no	256
Taimeh	2023	UK	Other Medical Care	129	39	82	English	yes	no	no	no	no	no	no	no	no	257
Tang	2017	China	Other Medical Care	253	50	100	Chinese	no	no	no	no	yes	no	no	no	no	258
Ten Brink	2021	UK	Other/Convenience	742	46	89	English	no	no	no	no	yes	no	no	no	no	259
Terluin	2022	Netherlands	Mixed	234	52	76	Dutch	yes	no	no	no	no	no	no	no	no	260
Thomas	2023	USA	Other/Convenience	399	41	77	English	no	no	no	no	yes	no	no	no	no	261
Thortveit	2019	Norway	Other/Convenience	2971	49	55	Norwegian	no	no	yes	no	no	no	no	no	no	262

© 2024 Hybelius J et al. *JAMA Network Open.*

First author	Year	Country	Sample	N	Age M	% Women	Language	fa[a]	txo	ico	cor	mns	cut	mci	trt	stc	Ref
Tian	2021	China	Other Medical Care	853	44	62	Chinese	no	no	yes	no	yes	no	no	no	no	263
Tietjen	2007	USA	Other Medical Care	1032	42	100	English	no	no	no	yes	no	no	no	no	no	264
Toussaint	2016	Germany	Psychosomatic Clinic	618	39	66	German	no	no	no	yes	yes	no	no	no	no	265
Toussaint	2017	USA	Primary Care, Mental H.	294	57	13	English	no	no	yes	yes	yes	no	yes	no	no	266
Toussaint	2017	Germany	General Population	2294	49	55	German	no	no	no	yes	yes	no	no	no	no	267
Toussaint	2019	Germany	Psychosomatic Clinic	372	39	69	German	no	no	yes	yes	yes	yes	no	no	no	268
Tucker	2021	USA	General Population	1877	56	58	English	no	no	yes	no	yes	no	no	no	no	269
Turp	2016	Germany	General Population	2515	49	53	German	no	no	no	no	yes	no	no	no	no	270
Uhlenbusch	2019	Germany	Other/Convenience	300	44	82	German	no	no	yes	no	no	no	no	no	no	271
Voigt	2012	Germany	Psychosomatic Clinic	456	45	61	German	no	no	no	no	yes	no	no	no	no	272
Walentynowicz	2018	Belgium	Students	1052	19	83	Dutch	yes	no	yes	yes	no	no	no	no	no	273
Wang	2023	Taiwan	Other Medical Care	98	51	66	Chinese	no	no	no	no	yes	no	no	no	no	274
Watanabe	2021	Japan	Psychosomatic Clinic	48	63	79	Japanese	no	no	no	no	yes	no	no	no	no	275
Weigel	2019	Germany	Other Medical Care	153	28	94	German	no	no	yes	yes	yes	no	no	no	no	276
Weisskirch	2021	USA	Students	459	21	80	English	no	no	no	yes	no	no	no	no	no	277
Weziak-Bialowolska	2016	Poland	General Population	5443	-	-	Polish	no	no	no	no	yes	no	no	no	no	278
Wilder-Smith	2023	Switzerland	Other Medical Care	43	31	86	Unclear	no	no	no	no	yes	no	no	no	no	279
Williams	2019	USA	Psychosomatic Clinic	56	40	73	English	no	no	no	no	yes	no	no	no	no	280
Williams	2022	USA	Other/Convenience	1003	37	64	English	no	no	no	no	yes	no	no	no	no	281
Wiltink	2017	Germany	Psychosomatic Clinic	1254	39	61	German	no	no	yes	no	no	no	no	no	no	282
Winkler	2020	Germany	Other/Convenience	930	28	72	German	no	no	no	yes	no	no	no	no	no	283
Witthöft	2013	Germany	General Population	414	47	54	German	yes	no	no	no	no	no	no	no	no	284
Witthöft	2016	Mixed	Students	4657	24	69	German	yes	no	no	no	no	no	no	no	no	285
Witthöft	2020	Mixed	Mixed	63	38	63	Mixed	no	no	no	no	yes	no	no	no	no	286
Wittmann	2021	Germany	Other Medical Care	270	37	72	German	no	no	no	yes	no	no	no	no	no	287
Wolfe	2014	USA	Other/Convenience	6110	65	75	English	no	no	no	no	yes	yes	no	no	no	288
Wolfe	2023	USA	Other/Convenience	33972	60	82	English	no	no	no	yes	yes	yes	no	no	no	289

© 2024 Hybelius J et al. *JAMA Network Open.*

First author	Year	Country	Sample	N	Age M	% Women	Language	fa[a]	txo	ico	cor	mns	cut	mci	trt	stc	Ref
Wong	2015	China	General Population	202	39	53	Chinese	no	no	yes	no	yes	no	no	no	no	290
Wunsch	2023	Mixed	Other/Convenience	1178	48	79	Mixed	no	no	no	no	yes	no	no	no	no	291
Yang	2020	South Korea	Other/Convenience	190	43	75	Korean	yes	no	yes	yes	no	no	no	yes	no	292
Yang	2023	China	Other Medical Care	1497	44	55	Chinese	no	no	no	yes	yes	no	no	no	no	293
Yap	2020	Singapore	Other/Convenience	400	19	52	English	no	no	no	yes	yes	no	no	no	no	294
Yap	2023	South Korea	Other Medical Care	772	38	70	Korean	no	no	no	yes	yes	no	no	no	no	295
Yap	2023	Indonesia	Students	507	22	86	English	no	no	no	yes	yes	no	no	no	no	296
Yazici Güleç	2012	Turkey	Mixed	440	28	53	Turkish	yes	no	yes	yes	no	no	no	yes	no	297
Yeon	2023	South Korea	Psychiatry	47	38	83	Korean	no	no	yes	no	yes	no	no	no	no	298
Zeng	2023	China	Other Medical Care	731	-	50	Chinese	no	no	no	yes	no	yes	no	no	no	299
Zhang	2014	China	Other Medical Care	404	44	66	Chinese	no	no	no	yes	no	no	no	no	no	300
Zhang	2016	China	Other Medical Care	1329	54	42	Chinese	yes	no	yes	yes	yes	no	no	no	no	301
Zhang	2022	China	Other Medical Care	746	33	37	Chinese	no	no	no	no	yes	no	no	no	no	302
Zhang	2023	Germany	Primary Care, General	359	36	57	German	no	no	no	no	yes	no	no	no	no	303
Zhou	2012	China	Other Medical Care	2391	49	62	Chinese	no	no	no	no	yes	no	no	no	no	304
Zhou	2020	Mixed	Students	829	22	66	Mixed	yes	no	yes	no	no	no	no	no	no	305
Zolotareva	2023	Russia	General Population	1153	41	51	Russian	yes	no	yes	no	no	no	no	no	no	306
Zwerenz	2017	Germany	Psychiatry	69	39	71	German	no	no	no	no	yes	no	no	no	no	307
de Gucht	2022	Netherlands	General Population	10291	42	63	Dutch	no	no	yes	no	no	no	no	no	no	308
de Vroege	2012	Netherlands	Other/Convenience	107	48	50	Dutch	no	no	no	no	yes	yes	no	no	no	309
van Niekerk	2022	Australia	Other/Convenience	318	31	100	English	no	no	yes	no	yes	no	no	no	no	310
van Niekerk	2022	Australia	Other/Convenience	227	31	100	English	no	no	yes	yes	yes	no	no	no	no	311
van Niekerk	2023	Australia	Other/Convenience	513	25	100	English	no	no	no	yes	no	no	no	no	no	312
van Ravesteijn	2013	Netherlands	Primary Care, Mental H.	117	47	75	Dutch	no	no	no	no	yes	no	no	no	no	313
van Selms	2022	Netherlands	Other Medical Care	1467	43	79	Dutch	no	no	no	no	yes	no	no	no	no	314
van Wouwe	2020	USA	Other Medical Care	30	47	80	English	no	no	no	no	yes	no	no	no	no	315
van der Feltz-Cornelis	2020	Netherlands	Psychosomatic Clinic	28	43	80	Dutch	no	no	no	no	yes	no	no	no	no	316

© 2024 Hybelius J et al. *JAMA Network Open.*

First author	Year	Country	Sample	N	Age M	% Women	Language	fa[a]	txo	ico	cor	mns	cut	mci	trt	stc	Ref
van der Feltz-Cornelis	2023	Netherlands	Psychosomatic Clinic	223	43	60	Dutch	no	no	yes	yes	yes	yes	no	no	no	317

[a] The nine rightmost columns before the reference column detail which analyses of measurement properties that were tabulated from each study: fa, factor analysis; txo, taxometric analysis; ico, evaluation of internal consistency; cor, correlations relevant for construct validity; mns, means from general population or patient sample; cut, evaluation of cut-offs; mci, evaluation of minimal important change; trt, evaluation of test-retest reliability; stc, evaluation of sensitivity to change.

© 2024 Hybelius J et al. *JAMA Network Open.*

eTable 5. Factor Analyses of the Patient Health Questionnaire–15

First author	Year	Sample	Language	N	FA type	Models evaluated[a]	Model preferred[a]
Cano-Garcia	2020	Primary Care, Mental H.	Spanish	1255	ESEM	1F, 4F, bf	bf
Dadfar	2020	Psychiatry	Iranian	155	PCA	N/A	4F (components)
Dadfar	2020	Students	Iranian	94	PCA	N/A	4F (components)
Dadfar	2020	Other/Convenience	Iranian	100	PCA	N/A	4F (components)
Dreher	2017	Psychiatry	German	109	PCA	N/A	4F (components)
Dreher	2017	Psychiatry	Vietnamese	110	PCA	N/A	3F (components)
Grover	2019	Psychiatry	Hindi	60	PCA	N/A	3F (components)
Habtamu	2022	Primary Care, General	Amharic	587	EFA	N/A	2F
Habtamu	2022	Primary Care, General	Amharic	587	EFA	N/A	3F
Kliem	2014	General Population	German	4903	EFA	N/A	3F
Körber[b]	2011	Primary Care, General	German	308	CFA	1F, 4F, hi, bf	bf
Lee	2011	General Population	Chinese	3014	PCA	N/A	4F (components)
Leonhart	2018	Psychosomatic Clinic	German	2517	CFA	bf	bf
Leonhart	2018	Mixed	Mixed	4302	CFA	bf	bf
Leonhart	2018	General Population	Chinese	1329	CFA	bf	bf
Leonhart	2018	Mixed	Dutch	456	CFA	bf	bf
Liao	2016	Mixed	Chinese	471	PCA	N/A	4F (components)
Liao	2016	Mixed	Chinese	471	CFA	3F	3F
Lyoo	2014	Other/Convenience	Korean	350	PCA	N/A	4F (components)
Nehme[b]	2023	Other/Convenience	Arabic	403	CFA	3F	3F
Nehme[b]	2023	Other/Convenience	Arabic	403	EFA	N/A	3F
Nordin	2013	General Population	Swedish	3406	PCA	N/A	1F
Schlechter	2023	Other/Convenience	Arabic	187	CFA	1F, 3F, 4F, bf, hi	4F
Schlechter	2023	Other/Convenience	Arabic	182	CFA	1F, 3F, 4F, bf, hi	4F
Stauder	2021	Other/Convenience	Hungarian	5020	ESEM	1F, bf	bf
Taimeh	2023	Other Medical Care	English	129	CFA	1F, 3F, 4F, bf	4F
Terluin	2022	Mixed	Dutch	234	CFA	1F, bf	bf

© 2024 Hybelius J et al. *JAMA Network Open.*

First author	Year	Sample	Language	N	FA type	Models evaluated[a]	Model preferred[a]
Walentynowicz	2018	Students	Dutch	1052	CFA	1F, 4F, hi, bf	bf
Witthöft	2016	Students	German	3053	CFA	1F, hi, bf	bf
Witthöft	2016	Students	German	1604	CFA	1F, hi, bf	bf
Witthöft	2013	General Population	German	414	EFA, CFA	1F, 4F, hi, bf	bf
Zhang	2016	Other Medical Care	Chinese	1329	EFA, CFA	1F, 3F, hi	3F
Zhou	2020	Students	German	416	CFA	1F, hi	hi
Zhou	2020	Students	Chinese	413	CFA	1F, hi	hi
Zolotareva	2023	General Population	Russian	1153	EFA, CFA	1F, 3F, bf	bf

Abbreviations: bf, a model with symptom-specific factors and a separate general somatic symptom burden factor; CFA, confirmatory factor analysis; EFA, exploratory factor analysis; ESEM, exploratory structural equation modeling; FA, factor analysis; hi, a model with symptom-specific factors and a second-order general somatic symptom burden factor; PCA, principal component analysis.
[a] See the original articles for detailed specifications which differed somewhat, including within studies.
[b] Results presented in secondary publication.[121,322]

© 2024 Hybelius J et al. *JAMA Network Open.*

eTable 6. Model Fit in Factor Analyses of the Patient Health Questionnaire–15

First author[a]	Model preferred[b]	Outcome 1F[c]				Outcome 4F[c]				Outcome bf[c]				Outcome hi[c]			
		CFI	TLI	RMSEA	SRMR	CFI	TLI	RMSEA	SRMR	CFI	TLI	RMSEA	SRMR	CFI	TLI	RMSEA	SRMR
Cano-Garcia	bf	0.85	0.82	0.11		0.99	0.98	0.04		1.00	1.00	0.01					
Körber[d]	bf	0.80	0.76	0.121		0.93	0.91	0.075		0.97	0.95	0.053		0.93	0.91	0.073	
Leonhart	bf									0.98	0.97	0.04					
Leonhart	bf									0.98	0.97	0.06					
Leonhart	bf									0.98	0.96	0.05					
Leonhart	bf									1.00	1.00	0.02					
Schlechter	4F	0.96	0.96	0.085	0.100	1.00	0.99	0.033	0.085					0.98	0.97	0.080	0.078
Schlechter	4F	0.97	0.96	0.082	0.101	0.99	0.99	0.036	0.083					0.98	0.97	0.076	0.084
Stauder	bf	0.95	0.94	0.060						0.99	0.98	0.037					
Taimeh	4F					0.96	0.95	0.042	0.059								
Terluin	bf	0.89	0.93	0.102	0.096					0.97	0.98	0.052	0.070				
Walentynowicz	bf	0.83		0.079		0.96		0.038		0.99		0.019		0.97		0.036	
Witthöft	bf	0.85	0.82	0.090						0.99	0.99	0.023		0.95	0.94	0.052	
Witthöft	bf	0.82	0.78	0.092						0.99	0.99	0.020		0.93	0.91	0.059	
Witthöft	bf	0.79	0.75	0.113		0.95	0.93	0.059		0.98	0.97	0.040		0.95	0.93	0.057	
Zhou	hi	0.72		0.086	0.065									0.91		0.049	0.049
Zhou	hi	0.83		0.079	0.057									0.94		0.050	0.042
Zolotareva	bf	0.85	0.81	0.096						0.97	0.96	0.044					

Abbreviations: bf, a model with symptom-specific factors and a separate general somatic symptom burden factor; CFI, comparative fit index; hi, a model with symptom-specific factors and a second-order general somatic symptom burden factor; RMSEA, root mean square error of approximation; SRMR, standardized root mean square residual; TLI, Tucker-Lewis index.
[a] Studies not reporting fit indices not included here. Empty cells = estimate not reported in the publication.
[b] See the original articles for detailed specifications which differed somewhat, including within studies.
[c] Fit indices refer to the best fitting models.
[d] Results presented in secondary publication.[121]

© 2024 Hybelius J et al. *JAMA Network Open.*

eTable 7. Factor Analyses of the Somatic Symptom Scale–8

First author	Year	Sample	Language	N	FA type	Models evaluated[a]	Model preferred	Outcome 1F[b]	Outcome hierarchical[b]
Dagnall	2023	General Population	English	1665	CFA	1F	1F	CFI=0.92, RMSEA=0.10, SRMR=0.04	N/A
Denovan	2019	Other/Convenience	English	170	EFA	1F	1F	CFI=0.91, RMSEA=0.13, SRMR=0.07	N/A
Ghapanch	2022	Mixed	Persian	122	CFA	1F, Gierk hierarchical	unclear	CFI=0.99, RMSEA=0.001	CFI=0.99, RMSEA=0.001
Gierk	2014	General Population	German	2510	CFA	1F, Gierk hierarchical	Gierk hierarchical	CFI=0.94, TLI=0.91, RMSEA=0.11	CFI=0.97, TLI=0.95, RMSEA=0.08
Goodarzi	2020	Other/Convenience	Persian	281	CFA	Gierk hierarchical	Gierk hierarchical	N/A	CFI=0.96, RMSEA=0.069
Li[c]	2022	Other Medical Care	Chinese	699	CFA	1F, 3F, Gierk hierarchical	3F	CFI=0.91, TLI=0.87, RMSEA=0.13	CFI=0.92, TLI=0.88, RMSEA=0.12
Petrelis	2022	Other Medical Care	Greek	138	CFA	Gierk hierarchical	Gierk hierarchical	N/A	CFI=0.98, TLI=0.96, RMSEA=0.06
Pollo	2022	Other Medical Care	Portuguese	300	CFA	1F	1F	CFI=1.00, TLI=1.00, RMSEA=0.015	N/A

Abbreviations: CFA, confirmatory factor analysis; CFI, comparative fit index; EFA, exploratory factor analysis; ESEM, exploratory structural equation modeling; FA, factor analysis; PCA, principal component analysis; RMSEA, root mean square error of approximation; TLI, Tucker-Lewis index.
[a] See the original articles for detailed specifications which differed somewhat, including within studies.
[b] Fit indices refer to the best fitting models.
[c] Results presented in secondary publication.[323]

© 2024 Hybelius J et al. *JAMA Network Open.*

eTable 8. Average Variance Extracted (AVE)[a] Pertaining to the General Somatic Symptom Burden Factor

	AVE[b]	Note
**PHQ-15**		
Cano-Garcia et al. (2020)	0.29	Bifactorial model
Leonhart et al. (2018)	0.30	Bifactorial model, Germany sample
Leonhart et al. (2018)	0.34	Bifactorial model, Netherlands sample
Leonhart et al. (2018)	0.31	Bifactorial model, China sample
Nordin et al. (2013)	0.29	Unifactorial model
Stauder et al. (2021)	0.45	Bifactorial model
Terluin et al. (2022)	0.39	Bifactorial model
Walentynowicz et al. (2018)	0.19	Bifactorial model
Witthöft et al. (2016)	0.22	Bifactorial model
Witthöft et al. (2013)	0.31	Bifactorial model
Zolotareva (2023)	0.29	Bifactorial model
**SSS-8**		
Gierk et al. (2014)[c]	0.51	Hierarchical model
Goodarzi et al. (2020)	0.30	Hierarchical model
Pollo et al. (2022)	0.34	Unifactorial model

Abbreviations: PHQ-15, the Patient Health Questionnaire 15; SSS-8, the Somatic Symptom Scale 8.

[a] The proportion of variance explained.

[b] Note that several studies did not report factor loadings (see eTable 6-7), and therefore did not allow for the calculation of AVE.

[c] Original (primary) publication.

© 2024 Hybelius J et al. *JAMA Network Open.*

eTable 9. Meta-Analysis of Cronbach α per Language, Setting, and Condition[a]

	Internal consistency of the PHQ-15						Internal consistency of the SSS-8					
	k	Pooled α[b]	95% CI	τ^2	I^2	*Q* (*df*)	k	Pooled α	95% CI	τ^2	I^2	*Q* (*df*)
Pooled total	89	0.81	0.80 to 0.82	0.01	96%	3610.9 (88)[c]	20	0.80	0.77 to 0.83	0.01	96%	406.9 (19)[c]
…Chinese (any) only	14	0.83	0.80 to 0.86	0.01	98%	1621.3 (13)[c]	1	-	-	-	-	-
…Dutch only	3	0.77	0.72 to 0.82	<0.01	94%	27.2 (2)[c]	0	-	-	-	-	-
…English only	16	0.80	0.79 to 0.81	<0.01	75%	41.3 (15)[c]	6	0.81	0.73 to 0.87	0.02	95%	107.9 (5)[c]
…German only	18	0.80	0.77 to 0.82	0.01	94%	302.0 (17)[c]	6	0.78	0.72 to 0.82	0.01	88%	41.7 (5)[c]
…Hungarian only	4	0.79	0.68 to 0.87	0.02	95%	34.6 (3)[c]	0	-	-	-	-	-
…Korean only	4	0.85	0.82 to 0.87	<0.01	47%	5.5 (3)	1	-	-	-	-	-
…Persian only	5	0.83	0.80 to 0.85	<0.01	48%	7.6 (4)	2	-	-	-	-	-
…Spanish only	4	0.79	0.77 to 0.80	<0.01	45%	4.8 (3)	0	-	-	-	-	-
…Swedish only	3	0.83	0.81 to 0.85	<0.01	86%	12.8 (2)[c]	0	-	-	-	-	-
Setting												
General population	10	0.80	0.79 to 0.82	<0.01	96%	164.4 (9)[c]	2	-	-	-	-	-
Primary care, general	5	0.80	0.78 to 0.82	<0.01	86%	23.2 (4)[c]	1	-	-	-	-	-
Primary Care, Mental H.	3	0.80	0.78 to 0.81	<0.01	0%	0.2 (2)	1	-	-	-	-	-
Psychiatry	16	0.83	0.82 to 0.85	<0.01	85%	138.2 (15)[c]	5	0.81	0.74 to 0.86	0.01	83%	32.0 (4)[c]
Psychosomatic specialist	6	0.81	0.76 to 0.85	0.01	94%	135.9 (5)[c]	2	-	-	-	-	-
Other medical care	24	0.82	0.79 to 0.84	0.01	96%	1814.5 (23)[c]	6	0.80	0.78 to 0.82	<0.01	50%	10.2 (5)
Non-psychiatric conditions												
Functional symptoms/syndromes[d]	4	0.82	0.78 to 0.86	<0.01	89%	13.9 (3)	1	-	-	-	-	-
Persistent pain, any	5	0.81	0.77 to 0.84	<0.01	78%	21.3 (4)	1	-	-	-	-	-
Persistent physical symptoms	2	-	-	-	-	-	0	-	-	-	-	-
Organic disease, any	11	0.79	0.75 to 0.83	0.01	88%	108.7 (10)[c]	2	-	-	-	-	-
Psychiatric disorders												
Anxiety disorder, any	0	-	-	-	-	-	0	-	-	-	-	-
Depression	5	0.86	0.85 to 0.87	<0.01	0%	2.5 (4)	1	-	-	-	-	-
Eating disorder, any	2	-	-	-	-	-	0	-	-	-	-	-
PTSD	1	-	-	-	-	-	0	-	-	-	-	-
Somatoform disorder, any	3	0.78	0.74 to 0.82	<0.01	0%	0.2 (2)	0	-	-	-	-	-
Pathological health anxiety	1	-	-	-	-	-	0	-	-	-	-	-
Somatic symptom disorder	0	-	-	-	-	-	0	-	-	-	-	-

© 2024 Hybelius J et al. *JAMA Network Open.*

Abbreviations: PHQ-15, Patient Health Questionnaire 15; PTSD, post-traumatic stress disorder; SSS-8, Somatic Symptom Scale 8.
[a] Meta-analysis was conducted using the Hakstian & Whalen method, for strata with at least 3 studies ("k"). Empty cells = estimate not reported in the publication.
[b] Values for alpha were transformed for the meta-analysis, and the resulting pooled estimates and τ^2 were then back-transformed. Whenever categories overlapped, or subcategories were reported in original studies, the same original study could contribute to multiple pooled estimates.
[c] $P < 0.05$
[d] Specific functional somatic syndromes could not be tabulated, though 1 study reported a PHQ-15 alpha for fibromyalgia, and 1 study reported a PHQ-15 alpha for non-cardiac chest pain.

© 2024 Hybelius J et al. *JAMA Network Open.*

eTable 10. Item-Total Correlations for the Patient Health Questionnaire–15

Study	Item-total correlations		
	ITC range	Corrected estimates?	n items with ITC <0.40
AlHadi (2017)	r = 0.03 – 0.58	Yes	4
Cano-García (2020)	r = 0.19 – 0.54	Unclear	4
Gierk (2015)	r = 0.19 – 0.58	Yes	8
Toussaint (2019)	r = 0.1 – 0.6	Unclear	8-9
Yazici Güleç (2012)	r = 0.21 – 0.56	Yes	6
Zhang (2016)	r = 0.15 – 0.56	Yes	4

Abbreviations: ITC, item-total correlations.

© 2024 Hybelius J et al. *JAMA Network Open.*

eTable 11. Item-Total Correlations for the Somatic Symptom Scale–8[a]

Study	Item-total correlations		
	ITC range	Corrected estimates?	n items with ITC <0.40
Ghapanch (2022)	r = 0.36 – 0.57	Unclear	1
Gierk (2014)	r >0.40 for all items	Yes	-
Gierk (2015)	r = 0.33 – 0.58	Yes	1
Goodarzi (2020)	r = 0.32 – 0.52	Yes	1
Petrelis (2022)	r = 0.46 – 0.64	Yes	-
Toussaint (2017)	r = 0.51 – 0.65	Yes	-
Toussaint (2019)	r = 0.2 – 0.4	Unclear	4 – 7

Abbreviations: ITC, item-total correlations.
[a] Empty cells = estimate not reported in the publication.

© 2024 Hybelius J et al. *JAMA Network Open.*

eTable 12. Sensitivity Analyses Focusing on Pearson Correlations Relevant for Construct Validity

	Pooled correlations with the PHQ-15[a]						Pooled correlations with the SSS-8[a]					
	K[b]	Pooled *r*	95% CI	Heterogeneity statistics			k[a]	Pooled *r*	95% CI	Heterogeneity statistics		
				τ^2	I^2	*Q* (*df*)				τ^2	I^2	*Q* (*df*)
Primary analysis (reference)												
Somatic symptom burden[c]	9	0.71	0.64 to 0.78	0.01	84%	59.7 (8)[f]	7	0.82	0.72 to 0.92	0.01	93%	136.2 (6)[f]
Health anxiety[d]	11	0.52	0.40 to 0.65	0.01	94%	293.5 (10)[f]	3	0.59	0.54 to 0.64	<0.01	14%	3.7 (2)
Symptom preoccupation[e]	5	0.45	0.39 to 0.51	<0.01	83%	30.3 (4)[f]	6	0.66	0.53 to 0.79	0.01	95%	171.1 (5)[f]
General anxiety	34	0.54	0.49 to 0.59	0.01	87%	279.7 (33)[f]	13	0.55	0.50 to 0.60	<0.01	62%	107.4 (12)[f]
Depression	53	0.62	0.56 to 0.67	0.01	95%	1020.0 (52)[f]	18	0.52	0.42 to 0.61	<0.01	93%	516.0 (17)[f]
Fisher's r-to-Z transformation												
Somatic symptom burden[c]	9	0.74	0.67 to 0.80	0.05	94%	100.0 (8)[f]	7	0.79	0.72 to 0.85	0.05	96%	213.6 (6)[f]
Health anxiety[d]	11	0.44	0.33 to 0.54	0.05	98%	223.2 (10)[f]	3	0.58	0.51 to 0.64	<0.01	40%	3.3 (2)
Symptom preoccupation[e]	5	0.45	0.38 to 0.51	0.01	77%	17.4 (4)[f]	6	0.55	0.43 to 0.66	0.04	93%	94.7 (5)[f]
General anxiety	34	0.55	0.52 to 0.59	0.02	91%	287.1 (33)[f]	13	0.56	0.49 to 0.62	0.03	96%	117.2 (12)[f]
Depression	53	0.58	0.54 to 0.62	0.04	95%	890.2 (52)[f]	18	0.56	0.47 to 0.64	0.07	99%	505.0 (17)[f]
English version only												
Somatic symptom burden[c]	0	-	-	-	-	-	1	0.79	0.75 to 0.83	-	-	-
Health anxiety[d]	3	0.57	0.53 to 0.60	<0.01	39%	11.4 (2)[f]	0	-	-	-	-	-
Symptom preoccupation[e]	0	-	-	-	-	-	0	-	-	-	-	-
General anxiety	7	0.60	0.55 to 0.65	<0.01	75%	33.3 (6)[f]	4	0.57	0.40 to 0.74	0.02	95%	88.3 (3)[f]
Depression	13	0.62	0.53 to 0.70	0.01	94%	241.9 (12)[f]	6	0.61	0.53 to 0.70	<0.01	85%	63.6 (5)[f]
General population only												
Somatic symptom burden[c]	0	-	-	-	-	-	1	0.88	0.87 to 0.89	-	-	-
Health anxiety[d]	1	0.43	0.40 to 0.46	-	-	-	0	-	-	-	-	-
Symptom preoccupation[e]	0	-	-	-	-	-	1	0.73	0.71 to 0.75	-	-	-
General anxiety	1	0.52	0.50 to 0.54	-	-	-	2	0.59	0.48 to 0.70	<0.01	93%	37.2 (1)[f]
Depression	1	0.75	0.74 to 0.76	-	-	-	3	0.57	0.47 to 0.66	0.01	96%	76.1 (2)[f]
Primary care, general, only												
Somatic symptom burden[c]	1	0.63	0.56 to 0.70	-	-	-	0	-	-	-	-	-
Health anxiety[d]	1	0.57	0.56 to 0.58	-	-	-	0	-	-	-	-	-
Symptom preoccupation[e]	0	-	-	-	-	-	0	-	-	-	-	-

© 2024 Hybelius J et al. *JAMA Network Open.*

	Pooled correlations with the PHQ-15[a]						Pooled correlations with the SSS-8[a]					
	K[b]	Pooled *r*	95% CI	Heterogeneity statistics			k[a]	Pooled *r*	95% CI	Heterogeneity statistics		
				τ^2	I^2	*Q* (*df*)				τ^2	I^2	*Q* (*df*)
General anxiety	1	0.64	0.61 to 0.67	-	-	-	0	-	-	-	-	-
Depression	1	0.72	0.70 to 0.74	-	-	-	0	-	-	-	-	-
Psychiatry only												
Somatic symptom burden[c]	0	-	-	-	-	-	0	-	-	-	-	-
Health anxiety[d]	2	0.50	0.41 to 0.59	0.01	18%	2.8 (1)	1	0.58	0.46 to 0.70	-	-	-
Symptom preoccupation[e]	1	0.50	0.42 to 0.58	-	-	-	1	0.40	0.25 to 0.55	-	-	-
General anxiety	0	-	-	-	-	-	0	-	-	-	-	-
Depression	5	0.64	0.61 to 0.67	<0.01	0%	1.1 (4)	0	-	-	-	-	-
Other secondary/tertiary care only												
Somatic symptom burden[c]	3	0.75	0.72 to 0.79	<0.01	20%	4.0 (2)	2	0.74	0.71 to 0.77	<0.01	0%	0.0 (1)
Health anxiety[d]	1	0.55	0.50 to 0.60	-	-	-	1	0.61	0.56 to 0.66	-	-	-
Symptom preoccupation[e]	1	0.52	0.47 to 0.57	-	-	-	3	0.57	0.47 to 0.68	<0.01	62%	11.0 (2)[f]
General anxiety	9	0.50	0.44 to 0.55	<0.01	72%	34.4 (8)[f]	2	0.58	0.52 to 0.64	<0.01	24%	3.1 (1)
Depression	13	0.58	0.54 to 0.62	<0.01	76%	56.9 (12)[f]	2	0.67	0.63 to 0.71	<0.01	0%	0.0 (1)
Other/convenience samples only												
Somatic symptom burden[c]	1	0.86	0.82 to 0.90	-	-	-	1	0.86	0.82 to 0.90	-	-	-
Health anxiety[d]	2	0.54	0.43 to 0.64	<0.01	67%	8.4 (1)[f]	0	-	-	-	-	-
Symptom preoccupation[e]	2	0.37	0.31 to 0.42	<0.01	0%	0.8 (1)	1	0.52	0.43 to 0.61	-	-	-
General anxiety	14	0.55	0.47 to 0.62	<0.01	89%	135.7 (13)[f]	6	0.55	0.52 to 0.58	<0.01	23%	36.4 (5)[f]
Depression	22	0.57	0.49 to 0.65	0.02	94%	372.5 (21)[f]	10	0.51	0.42 to 0.60	<0.01	92%	374.6 (9)[f]

Abbreviations: PHQ-15, Patient Health Questionnaire 15; SSS-8, Somatic Symptom Scale 8.
[a] For the Z-transformed sensitivity analyses, τ2 was also back-transformed. The other sensitivity analyses were based on the primary method of pooling Pearson correlations, namely the Hunter-Schmidt method, and were conducted regardless of the number of studies (k). Empty cells = estimate not reported in the publication.
[b] Number of studies included in random effects meta-analysis.
[c] "Somatic symptom burden" refers to other measures that focus on the subjective experience of physical symptoms.
[d] "Health anxiety" refers to the fear of, or preoccupation with, having or developing a serious disease.
[e] "Symptom preoccupation" refers to the tendency to respond strongly to, and engage in behaviors contingent on, somatic symptoms.
[f] $P < 0.05$

© 2024 Hybelius J et al. *JAMA Network Open.*

eTable 13. Sensitivity Analyses Focusing on Pearson Correlations vs Other Constructs: Low Risk of Bias

	k[a]	Pooled r (95% CI)	Heterogeneity statistics		
			τ^2	I^2	*Q* (*df*)
**PHQ-15**					
Somatic symptom burden[b]	6	0.70 (0.64-0.76)	<0.01	77%	31.5 (5)[e]
Health anxiety[c]	8	0.55 (0.39-0.70)	0.01	91%	238.9 (7)[e]
Symptom preoccupation[d]	4	0.40 (0.34-0.45)	<0.01	43%	7.1 (3)
General anxiety	25	0.54 (0.48-0.60)	0.01	89%	241.2 (24)[e]
Depression	35	0.59 (0.55-0.64)	0.01	90%	351.8 (34)[e]
**SSS-8**					
Somatic symptom burden[b]	4	0.86 (0.80-0.92)	<0.01	88%	67.8 (3)[e]
Health anxiety[c]	2	0.53 (0.44-0.62)	<0.00	0%	1.0 (1)
Symptom preoccupation[d]	5	0.68 (0.51-0.84)	0.01	91%	115.4 (4)[e]
General anxiety	10	0.55 (0.51-0.59)	<0.01	49%	63.3 (9)[e]
Depression	13	0.51 (0.45-0.58)	<0.01	88%	226.6 (12)[e]

Abbreviations: PHQ-15, Patient Health Questionnaire 15; SSS-8, Somatic Symptom Scale 8.
[a] Number of studies included in random effects meta-analysis.
[b] "Somatic symptom burden" refers to other measures that focus on the subjective experience of physical symptoms.
[c] "Health anxiety" refers to the fear of, or preoccupation with, having or developing a serious disease.
[d] "Symptom preoccupation" refers to the tendency to respond strongly to, and engage in behaviors contingent on, somatic symptoms.
[e] $P < 0.05$

© 2024 Hybelius J et al. *JAMA Network Open.*

eTable 14. Sensitivity Analyses of Mean Scores Based on the English Version and Low Risk of Bias Due to Sampling Only[a]

	Average sum score on the PHQ-15					Average sum score on the SSS-8				
	**k**	**Pooled M (95% CI)**	**τ^2**	**I^2**	***Q* (*df*)**	**k**	**Pooled M (95% CI)**	**τ^2**	**I^2**	***Q* (*df*)**
General population	1	-	-	-	-	1	-	-	-	-
…weighted for 50% women	-	-	-	-	-	-	-	-	-	-
Primary care, general	4	9.8 (7.1-12.4)	7.3	99%	185.0 (3)[b]	1	-	-	-	-
Primary Care, Mental H.	3	12.2 (9.2-15.1)	6.4	98%	147.8 (2)[b]	1	-	-	-	-
Psychiatry	3	9.0 (8.7-9.3)	0.0	66%	5.6 (2)	1	-	-	-	-
Psychosomatic specialist	1	-	-	-	-	0	-	-	-	-
Other medical care	22	12.0 (10.9-13.1)	6.5	98%	1000.5 (21)[b]	0	-	-	-	-
Psychiatric disorders, low risk of bias										
Anxiety disorder, any	1	13.1 (10.5-15.7)	-	-	-	0	-	-	-	-
Depression	3	11.8 (10.0-13.6)	2.3	97%	58.7 (2)[b]	2	11.9 (11.1-12.6)	0.0	0%	0.6 (1)
Somatoform disorder, any	13	12.9 (11.7-14.2)	5.1	97%	353.1 (12)[b]	4	13.3 (11.6-14.9)	2.1	85%	29.2 (3)[b]
Pathological health anxiety	2	14.0 (13.0-15.1)	0.0	0%	0.6 (1)	0	-	-	-	-
Somatic symptom disorder	6	13.5 (11.7-15.4)	5.3	98%	209.3 (5)[b]	3	13.2 (10.9-15.5)	3.2	91%	28.9 (2)[b]

Abbreviations: PHQ-15, Patient Health Questionnaire 15; SSS-8, Somatic Symptom Scale 8.

[a] Meta-analysis was conducted for strata with at least 3 studies (“k”). Whenever categories overlapped, or subcategories were reported in original studies, the same original study could contribute to multiple pooled estimates. Empty cells = estimate not reported in the publication.

[b] $P < 0.05$

© 2024 Hybelius J et al. *JAMA Network Open.*

eTable 15. Clinical Cutoffs on the PHQ-15 According to Setting[a]

Study	Setting	N	Country	Condition	AUC	Cut-off[b]			Risk of bias[c]
						Symmetrically weighted Se/Sp	High sensitivity	High specificity	
Laferton (2017)	General population	250	Germany	DSM-5 SSD	0.79	6 (Se = 0.66, Sp = 0.80)	4 (Se = 0.87, Sp = 0.53)	7 (Se = 0.52, Sp = 0.85)	2/4
Liao (2016)	Psychiatry + healthy	471	Taiwan	DSM-IV-TR somatoform disorder	0.68	7 (Se = 0.67, Sp = 0.60)	-	-	1/4
Liao (2016)	Psychiatry + healthy	471	Taiwan	DSM-5 SSD and related disorder	0.73	5 (Se = 0.85, Sp = 0.49)	-	-	1/4
Cao (2020)[d]	Hospitals + Psychosom	697	China	DSM-5 SSD	0.72	-	-	-	0/4
Cao (2020)[d]	General hospitals	224	China	DSM-5 SSD	-	8 (Se = 0.79, Sp = 0.54)	-	-	0/4
Zeng (2023)	General hospitals	731	China	DSM-5 SSD	0.63	-	-	-	0/4
de Vroege (2012)	Sick-listed employees	107	Netherlands	Somatoform condition	0.63	10 (Se = 0.52, Sp = 0.70)	8 (Se = 0.61, Sp = 0.55)	10 (Se = 0.52, Sp = 0.70)	2/4
Körber.(2011)	Primary care	308	Germany	Somatoform or pain disorder	0.76	10 (Se = 0.80, Sp = 0.59)	9 (Se = 0.85, Sp = 0.53)	12 (Se = 0.62, Sp = 0.76)	2/4
Cao (2020)[d]	Psychosom	239	China	DSM-5 SSD	-	11 (Se = 0.67, Sp = 0.62)	-	-	0/4
Herzog (2015)	Psychosom	262	Germany	Somatoform disorder	0.67	-	-	-	0/4
van der Feltz-Cornelis (2023)	Psychosom	218	Netherlands	DSM-5 SSD and related disorder	0.65	13 (Se = 0.70, Sp = 0.52)	13 (Se = 0.70, Sp = 0.52)	-	0/4
Toussaint (2020)	Psychosom	372	Germany	DSM-5 SSD	0.70	14 (Se =0.60, Sp =0.69)	12 (Se = 0.71, Sp = 0.56)	15 (Se = 0.54, Sp = 0.80)	2/4

Abbreviations: DSM-IV-TR, Diagnostic and Statistical Manual of Mental Disorders, fourth edition, text revision; DSM-5, Diagnostic and Statistical Manual of Mental Disorders, fifth edition; SSD, somatic symptom disorder; AUC, General area under the curve; Se, Sensitivity; Sp, Specificity; Psychosom, specialized psychosomatic secondary care clinics.

[a] Empty cells = estimate not reported in the publication.

[b] In the PHQ-15 original publication, 5 was suggested to represent low, 10 medium, and 15 high somatic symptom burden.[318]

[c] Number of domains deemed at high risk of bias according to assessment with a tailored version of QUADAS-2.[319] The four domains encompassed by the tool concerned (i) patient selection, (ii) the index test, (iii) the reference standard, and (iv) flow and timing in recruitment, administration of tests, and data analysis.

[d] Estimate from a secondary publication.[320]

© 2024 Hybelius J et al. *JAMA Network Open.*

eTable 16. Clinical Cutoffs on the SSS-8 According to Setting

Study	Setting	N	Country	Condition	AUC	Cut-off[a]			Risk of bias[b]
						Symmetrically weighted Se/Sp	High sensitivity	High specificity	
Cao (2020)[c]	Hospitals + Psychosom	697	China	DSM-5 SSD	0.73	9 (Se = 0.674, Sp = 0.68)	7 (Se = 0.77, Sp = 0.54)	11 (Se = 0.52, Sp = 0.78)	0/4
Toussaint (2020)	Psychosom	372	Germany	DSM-5 SSD	0.71	14 (Se =0.60, Sp = 0.72)	11 (Se = 0.76, Sp = 0.50)	15 (Se = 0.52, Sp = 0.73)	2/4

Abbreviations: DSM-IV-TR, Diagnostic and Statistical Manual of Mental Disorders, fourth edition, text revision; DSM-5, Diagnostic and Statistical Manual of Mental Disorders, fifth edition; SSD, somatic symptom disorder; AUC, General area under the curve; Se, Sensitivity; Sp, Specificity; Psychosom, specialized psychosomatic secondary care clinics

[a] In the SSS-8 original publication, 0-3 was suggested to represent no or minimal, 4-7 low, 8-11 medium, 12-16 high, and 16-32 very high somatic symptom burden.[321]

[b] Number of domains deemed at high risk of bias according to assessment with a tailored version of QUADAS-2.[319] The four domains encompassed by the tool concerned (i) patient selection, (ii) the index test, (iii) the reference standard, and (iv) flow and timing in recruitment, administration of tests, and data analysis.

[c] Estimate from a secondary publication.[320]

© 2024 Hybelius J et al. *JAMA Network Open.*

eTable 17. Test-Retest Reliability[a]

Study	Sample	N	Time frame	Reliability		
				Pearson r	Spearman rho	ICC
**PHQ-15**						
Liao (2016)	Mixed psychiatry + healthy controls	unclear	2 weeks	0.93		
Han (2009)	General psychiatry	57	2 weeks	0.65		
Lee (2011)	General population	200	1 month		0.71	
Yazici Güleç (2012)	Students	60	1 month		0.54	
Lyoo (2014)	Students	19	max 10 days			0.87
**SSS-8**						
Petrelis (2022)	Chronic low back pain, specialized care	20	1 week	1.00[b]		
Ghapanch (2022)	General population	63	2 weeks			0.89
Yang (2020)	Psychiatry outpatients with somatic complaints + healthy controls	31	3-4 weeks	0.78		

Abbreviations: PHQ-15, Patient Health Questionnaire 15; ICC, intraclass correlation coefficient; SSS-8, Somatic Symptom Scale 8.
[a] Empty cells = estimate not reported in the publication.
[b] r = 0.996

© 2024 Hybelius J et al. *JAMA Network Open.*

eFigure 2. Effect Sizes for Cognitive-Behavioral Therapy Compared With Rudimentary Controls on Somatic Symptom Burden, as Measured Using the PHQ-15 in Somatoform Conditions and Functional Somatic Symptoms and Syndromes

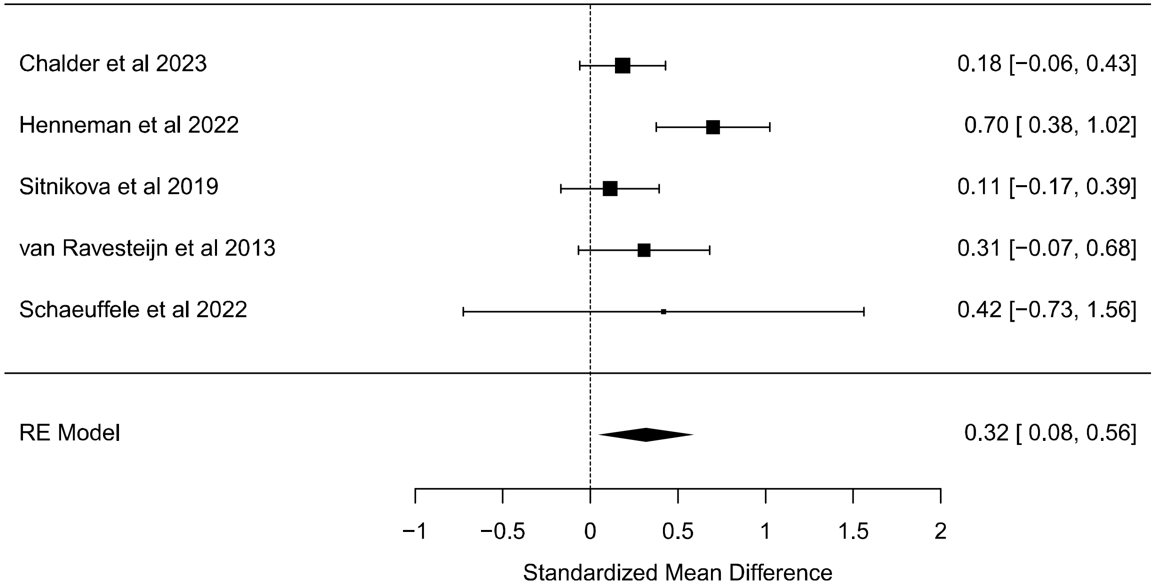

Effect of cognitive-behavioral therapy on somatic symptom burden as measured by the Patient Health Questionnaire 15 (PHQ-15) post-treatment. All studies recruited participants with somatoform conditions or functional somatic symptoms and syndromes. In 3 of 5 studies,[48,245,313] the control condition was standard medical care. In 2 of 5 studies,[104,236] the comparator was a waitlist.

© 2024 Hybelius J et al. *JAMA Network Open.*

eTable 18. Indicators of Possible Publication Bias

Domain	Visual inspection of funnel plot	Egger's test	Duval & Tweedie (R0 estimator)	Possible implication[a]
PHQ-15				
Cronbach's α (overall)	Not clearly skewed	z = -1.90, P = 0.058	0 studies missing	-
Mean (5 largest meta-analyses)				
Other medical care	Possibly skewed	z = 1.48, P = 0.139	5 studies missing on left side	M decreased from 10.4 to 10.0
Functional symptoms/syndromes	Not clearly skewed	z = 0.22, P = 0.827	0 studies missing	-
Organic disease, any	Possibly skewed	z = 1.35, P = 0.176	2 studies missing on left side	M decreased from 9.3 to 8.9
Persistent pain, any	Not clearly skewed	z = 0.16, P = 0.869	0 studies missing	-
Psychiatry	Possibly skewed	z = 1.73, P = 0.084	1 study missing on left side	M decreased from 10.7 to 10.5
Pearson's r (main)				
Somatic symptom burden	Possibly skewed	z = 1.01, P = 0.310	0 studies missing	-
General anxiety	Not clearly skewed	z = 0.15, P = 0.882	1 study missing on left side	r decreased from 0.5418 to 0.5417
Depression	Clearly skewed	z = -2.29, P = 0.022	22 studies missing on right side	r increased from 0.62 to 0.67
Hedges' g	Not clearly skewed	z = 0.38, P = 0.702	0 studies missing	-
SSS-8				
Cronbach's α (overall)	Not clearly skewed	z = -1.91, P = 0.056	0 studies missing	-
Mean (5 largest meta-analyses)				
Other medical care	Not clearly skewed	z = 0.78, P = 0.434	0 studies missing	-
General population	Not clearly skewed	z = 0.19, P = 0.853	2 studies missing on left side	M decreased from 6.9 to 4.9
Functional symptoms/syndromes	Possibly skewed	z = 1.01, P = 0.313	0 studies missing	-
Organic disease, any	Not clearly skewed	z = 0.87, P = 0.385	0 studies missing	-
Persistent pain, any[b]	Possibly skewed	z = 0.93, P = 0.354	0 studies missing	-
Psychiatry[b]	Not clearly skewed	z = 0.49, P = 0.627	0 studies missing	-
Somatoform disorder, any[b]	Possibly skewed	z = -1.12, P = 0.264	0 studies missing	-
Pearson's r (main)				
Somatic symptom burden	Possibly skewed	z = -1.46, P = 0.145	0 studies missing	-
General anxiety	Not clearly skewed	z = 0.10, P = 0.918	0 studies missing	-
Depression	Possibly skewed	z = 0.48, P = 0.634	0 studies missing	-

Abbreviations: PHQ-15, Patient Health Questionnaire 15; SSS-8, Somatic Symptom Scale 8.
[a] Empty cells = not applicable due to 0 studies missing.
[b] Tied for fifth largest meta-analysis pertaining to SSS-8 means.

© 2024 Hybelius J et al. *JAMA Network Open.*

eTable 19. Summary of Targets, Hypotheses, Outcomes, and Conclusions

Domain		Patient Health Questionnaire 15 (PHQ-15)			Somatic Symptom Scale 8 (SSS-8)[a]		
Name	Focus of domain	Preregistered target or hypothesis	Outcome	Conclusion	Preregistered target or hypothesis	Outcome	Conclusion
Factor structure (dimensionality)	What configuration of latent variables best explains the variance between respondents?	Approximate Witthöft et al. bifactorial model	Bifactorial model usually best fit (13/16 studies), with some items dropped.<br><br>AVE for the general factor = 0.19-0.45	Bifactorial, corroborated<br><br>General factor of moderate strength	Approximate Gierk et al. hierarchical model<br><br>AVE for the general factor = 0.30-0.51	Gierk model usually best fit (4/5 studies)	Gierk model, corroborated<br><br>General factor of moderate strength
Taxometric analysis	Is somatic symptom burden best regarded as a dimensional of categorical phenomenon?	No preregistered target, but assumed to be dimensional.	Dimensional model usually best fit (14/15 CCFIs)	Dimensional	No preregistered target, but assumed to be dimensional.	-	-
Internal consistency	Are the items of the instrument correlated and exhaustive?	Cronbach αs ≥ 0.70<br>Most ITCs ≥ 0.40-0.50	Overall pooled α = 0.81, with minimal heterogeneity. Several very low ITCs.	Overall adequate internal consistency, but with redundant items	Cronbach αs ≥ 0.70<br>Most ITCs ≥ 0.40-0.50	Overall pooled α = 0.80, with minimal heterogeneity. Few low ICTs.	Adequate internal consistency
Construct validity	From a theoretical standpoint, are the correlations versus other constructs reasonable?	Somatic symptom burden: r in the higher range of 0.40-0.85<br><br>Health anxiety and other forms of symptom preoccupation: r in the lower range of 0.40-0.85<br><br>General anxiety and depression: r ≈ 0.30-0.60	Somatic symptom burden: r = 0.71<br><br>Health anxiety and similar: r = 0.45-0.52<br><br>General anxiety and depression: r = 0.54-0.62	Passable construct validity.	Somatic symptom burden: r in the higher range of 0.40-0.85<br><br>Health anxiety and other forms of symptom preoccupation: r in the lower range of 0.40-0.85<br><br>General anxiety and depression: r ≈ 0.30-0.60	Somatic symptom burden: r = 0.82<br><br>Health anxiety and similar: r = 0.59-0.66<br><br>General anxiety and depression: r = 0.52-0.55	Adequate construct validity

© 2024 Hybelius J et al. *JAMA Network Open.*

Domain		Patient Health Questionnaire 15 (PHQ-15)			Somatic Symptom Scale 8 (SSS-8)[a]		
**Name**	**Focus of domain**	**Preregistered target or hypothesis**	**Outcome**	**Conclusion**	**Preregistered target or hypothesis**	**Outcome**	**Conclusion**
Mean scores	What are typical mean scores in general population and various clinical samples?	Expectation similar to primary publication. For example, general population M ≈ 3.8.	Pooled general population M = 4.1[a]<br><br>For more means, see Table 2	Reasonable and expected mean scores	Expectation similar to primary publication. For example, general population M ≈ 3.2.	Pooled general population M = 7.8[b,c]<br><br>For more means, see Table 2	Mostly reasonable, but pooled mean for the general population higher than expected
Cut-offs	What are optimal cut-offs to identify somatoform disorders?	Based on primary publication, expected to lie around 10-15 for typical screening purposes	For symmetrically weighted sensitivity and specificity: around 6 in the general population, 8-14 in various care settings.	Optimal cut-offs largely in line with expectations, and differ depending on situation and setting.	Based on primary publication, expected to lie around 12-16 for typical screening purposes	For symmetrically weighted sensitivity and specificity: 9 in a mixed sample, 14 in specialist care.	Optimal cut-offs largely in line with expectations, and differ depending on situation and setting.
Minimal clinically important difference	What is the minimal clinically important score difference?	Exploratory only.	Estimated in one study as 2.3, with one item excluded	ca 3	Exploratory only.	Based on two studies: 3	ca 3
Test-retest reliability	How consistent results are seen when responses are given repeatedly over time?	Over ca 2 weeks: rs ≥ ca 0.70 and ICCs ≥ 0.50, ideally ≥ 0.75	10-14 days: r=0.65, r=0.93, ICC=0.87	Inconsistent test-retest reliability.	Over ca 2 weeks: rs ≥ ca 0.70 and ICCs ≥ 0.50, ideally ≥ 0.75	7-14 days: r=0.996, ICC=0.89	Probably adequate test-retest reliability.
Sensitivity to change (responsiveness)	Is the instrument sensitive to change of the kind which it is intended to measure?	Moderate (g ≥ ca 0.35) pooled RCT effect of CBT versus rudimentary controls for somatic symptom distress.<br><br>Correlations of r ≈ 0.30-0.70 in change versus change in other measures of somatic	Pooled effect of CBT: g=0.32 (95% CI: 0.08 to 0.56).<br><br>Correlation with change in symptom	Probably sensitive to change.	Moderate (g ≥ ca 0.35) pooled RCT effect of CBT versus rudimentary controls for somatic symptom distress.<br><br>Correlations of r ≈ 0.30-0.70 in change versus change in other measures of somatic symptom	During treatment for psychiatric disorders, correlation with change in anxiety: r = 0.68, depression: r=0.62, and	Lack of data relevant for assessing preregistered criteria. Unclear.

© 2024 Hybelius J et al. *JAMA Network Open.*

Domain		Patient Health Questionnaire 15 (PHQ-15)			Somatic Symptom Scale 8 (SSS-8)[a]		
Name	Focus of domain	Preregistered target or hypothesis	Outcome	Conclusion	Preregistered target or hypothesis	Outcome	Conclusion
		symptom burden or symptom preoccupation.	preoccupation : r=0.68		burden or symptom preoccupation.	functional impairment: r = 0.51.	

[a] Empty cells = no studies included in this systematic review and meta-analysis reported this outcome.
[b] This mean was weighted to represent a sample with 50% women.
[c] Indicators of publication bias for the pooled general population mean on the SSS-8 were contradictory. See eTable 18 for details on possible publication bias.

© 2024 Hybelius J et al. *JAMA Network Open.*

eReferences.

1. Hakstian AR, Whalen TE. A k-sample significance test for independent alpha coefficients. Psychometrika. 1976 Jun 1;41(2):219–31.

2. Field AP. Is the Meta-Analysis of Correlation Coefficients Accurate When Population Correlations Vary? Psychol Methods. 2005;10(4):444–67.

3. Hedges LV. Distribution Theory for Glass's Estimator of Effect size and Related Estimators. J Educ Stat. 1981 Jun 1;6(2):107–28.

4. Hedges LV. Estimation of effect size from a series of independent experiments. Psychol Bull. 1982;92(2):490–9.

5. Cohen J. 2.2.3 "Small," "medium", and "large" d values. In: Statistical power analysis for the behavioral sciences. 2nd ed. Lawrence Erlbaum Associates; p. 24–7.

6. Higgins JPT, Thompson SG, Deeks JJ, Altman DG. Measuring inconsistency in meta-analyses. BMJ. 2003 Sep 4;327(7414):557–60.

7. Fisher RA. On the "probable error" of a coefficient of correlation deduced from a small sample. Metron. 1921;(1):1–32.

8. Carter JV, Pan J, Rai SN, Galandiuk S. ROC-ing along: Evaluation and interpretation of receiver operating characteristic curves. Surgery. 2016 Jun 1;159(6):1638–45.

9. Rodriguez MC, Maeda Y. Meta-analysis of coefficient alpha. Psychol Methods. 2006;11(3):306–22.

10. Sánchez-Meca J, López-López JA, López-Pina JA. Some recommended statistical analytic practices when reliability generalization studies are conducted. Br J Math Stat Psychol. 2013;66(3):402–25.

11. Viechtbauer W. Conducting Meta-Analyses in R with the metafor Package. J Stat Softw. 2010 Aug 5;36:1–48.

12. Reilly TJ, MacGillivray SA, Reid IC, Cameron IM. Psychometric properties of the 16-item Quick Inventory of Depressive Symptomatology: a systematic review and meta-analysis. J Psychiatr Res. 2015 Jan;60:132–40.

13. Kroenke K, Spitzer RL, Williams JB. The PHQ-15: validity of a new measure for evaluating the severity of somatic symptoms. Psychosom Med. 2002 Mar;64(2):258–66.

14. Gierk B, Kohlmann S, Kroenke K, Spangenberg L, Zenger M, Brähler E, et al. The somatic symptom scale-8 (SSS-8): a brief measure of somatic symptom burden. JAMA Intern Med. 2014 Mar;174(3):399–407.

15. Henningsen P, Fink P, Hausteiner-Wiehle C, Rief W. Terminology, classification and concepts. In: Creed F, Henningsen P, Fink P, editors. Medically unexplained symptoms, somatisation and bodily distress: Developing better clinical services. Cambridge University Press; 2011. p. 43–68.

16. Bolen B. How an Organic Disease Differs From a Functional Disorder [Internet]. Verywell Health. 2023 [cited 2024 Jan 16]. Available from: https://www.verywellhealth.com/organic-disease-1944921

17. Apkarian AV, Baliki MN, Geha PY. Towards a theory of chronic pain. Prog Neurobiol. 2009 Feb 1;87(2):81–97.

18. Löwe B, Andresen V, Van Den Bergh O, Huber TB, Von Dem Knesebeck O, Lohse AW, et al. Persistent SOMAtic symptoms ACROSS diseases — from risk factors to modification: scientific framework and overarching protocol of the interdisciplinary SOMACROSS research unit (RU 5211). BMJ Open. 2022 Jan;12(1):e057596.

© 2024 Hybelius J et al. *JAMA Network Open.*

19. Abasi I, Ghapanchi A, Toussaint A, Bitarafan M, Zarabi H, Derakhshan FS, et al. Psychometric properties of the Persian version of the somatic symptom disorder-B Criteria Scale (SSD-12) in community and clinical samples. Gen Hosp PSYCHIATRY. 2022 Sep;78:1–8.

20. Adewuya AO, Atilola O, Ola BA, Coker OA, Zachariah MP, Olugbile O, et al. Current prevalence, comorbidity and associated factors for symptoms of depression and generalised anxiety in the Lagos State Mental Health Survey (LSMHS), Nigeria. Compr Psychiatry. 20171128th ed. 2018 Feb;81:60–5.

21. Aguirre Cárdenas C, Oñederra MC, Esparza Benavente C, Durán J, González Tugas M, Gómez-Pérez L. Psychometric Properties of the Fibromyalgia Survey Questionnaire in Chilean Women With Fibromyalgia. J Clin Rheumatol. 2021 Sep 1;27(6S):S284–93.

22. Ahmed A, Aslam N. The role of tinnitus distress in the development of somatization symptoms among patients: implications for health care in Pakistan. Int J Hum Rights Healthc. 2022;16(4):341–51.

23. Ak AK, Celebisoy N, Ozdemir HN, Gokcay F. Vestibular migraine and persistent postural perceptual dizziness: Handicap, emotional comorbidities, quality of life and personality traits. Clin Neurol Neurosurg. 20220805th ed. 2022 Oct;221:107409.

24. AlHadi AN, AlAteeq DA, Al-Sharif E, Bawazeer HM, Alanazi H, AlShomrani AT, et al. An arabic translation, reliability, and validation of Patient Health Questionnaire in a Saudi sample. Ann Gen Psychiatry. 20170906th ed. 2017;16:32.

25. Almwled AS, Almuhaydili AO, Altamimi SM, Alzahrani MA, Alnahdi RK, Almotairi SB, et al. Prevalence and biopsychosocial factors associated with treatment adherence among people with epilepsy in a tertiary care hospital in Riyadh, Saudi Arabia. Neurosci Riyadh. 2022 Apr;27(2):94–103.

26. Alosaimi FD, Labani R, Almasoud N, Alhelali N, Althawadi L, AlJahani DM. Associations of foot ulceration with quality of life and psychosocial determinants among patients with diabetes; a case-control study. J Foot Ankle Res. 20191210th ed. 2019;12:57.

27. Altamura M, D'Andrea G, Angelini E, Tortorelli FMP, Balzotti A, Porcelli P, et al. Psychosomatic syndromes are associated with IL-6 pro-inflammatory cytokine in heart failure patients. PLoS One. 20220310th ed. 2022;17(3):e0265282.

28. Althaus A, Broicher W, Wittkamp P, Andresen V, Lohse AW, Löwe B. Determinants and frequency of irritable bowel syndrome in a German sample. Z Gastroenterol. 20160404th ed. 2016 Mar;54(3):217–25.

29. Andreasson A, McNaughton D, Beath A, Lodin K, Wicksell RK, Lekander M, et al. Properties of the Sickness Questionnaire in an Australian sample with chronic medically unexplained symptoms. Brain Behav Immun Health. 20200310th ed. 2020 Mar;3:100059.

30. Arsenakis S, Chatton A, Penzenstadler L, Billieux J, Berle D, Starcevic V, et al. Unveiling the relationships between cyberchondria and psychopathological symptoms. J Psychiatr Res. 20210902nd ed. 2021 Nov;143:254–61.

31. Barends H, Claassen-van Dessel N, van der Wouden JC, Twisk JWR, Terluin B, van der Horst HE, et al. Impact of symptom focusing and somatosensory amplification on persistent physical symptoms: A three-year follow-up study. J Psychosom Res. 20200508th ed. 2020 Aug;135:110131.

32. Baum P, Do L, Deterding L, Lier J, Kunis I, Saur D, et al. Cardiac function in relation to functional status and fatigue in patients with post-COVID syndrome. Sci Rep. 20221115th ed. 2022 Nov 15;12(1):19575.

33. Becker J, Kreis A, Schorch T, Mayer A, Tsiouris A, Beutel ME, et al. Adherence and effectiveness of an emotion-based psychodynamic online self-help during and after inpatient and day-care psychotherapy: Results of a naturalistic study. Front Psychiatry. 20230120th ed. 2023;14:1027118.

34. Beiner E, Brenner Miguel S, Friederich HC, Tesarz J, Per PC. Elevated high sensitive C-reactive protein in fibromyalgia. Front Psychiatry. 20231130th ed. 2023;14:1237518.

35. Berens S, Schaefert R, Baumeister D, Gauss A, Eich W, Tesarz J. Does symptom activity explain psychological differences in patients with irritable bowel syndrome and inflammatory bowel disease? Results from a multi-center cross-sectional study. J Psychosom Res. 20190913th ed. 2019 Nov;126:109836.

36. Berens S, Dong Y, Fritz N, Walstab J, D'Amato M, Zheng T, et al. Serotonin type 3 receptor subunit gene polymorphisms associated with psychosomatic symptoms in irritable bowel syndrome: A multicenter retrospective study. World J Gastroenterol. 2022 Jun 7;28(21):2334–49.

37. Black CJ, Yiannakou Y, Houghton LA, Ford AC. Epidemiological, Clinical, and Psychological Characteristics of Individuals with Self-reported Irritable Bowel Syndrome Based on the Rome IV vs Rome III Criteria. Clin Gastroenterol Hepatol. 20190531st ed. 2020 Feb;18(2):392-398 e2.

38. Boecking B, Biehl R, Brueggemann P, Mazurek B. Health-Related Quality of Life, Depressive Symptoms, Anxiety, and Somatization Symptoms in Male and Female Patients with Chronic Tinnitus. J Clin Med [Internet]. 20210625th ed. 2021 Jun 25;10(13). Available from: https://www.ncbi.nlm.nih.gov/pubmed/34202097

39. Borho A, Morawa E, Schmitt GM, Erim Y. Somatic distress among Syrian refugees with residence permission in Germany: analysis of a cross-sectional register-based study. BMC Public Health. 20210512th ed. 2021 May 12;21(1):896.

40. Bornstein RF, Porcerelli JH, Jones JR. Dependency, Detachment, and Health-Related Behavior in Adult Primary Care Patients. J Clin Psychol Med Settings. 20230212th ed. 2023 Dec;30(4):699–707.

41. Brand S, Petzke TM, Witthöft M. The Differential Relationship Between Self-Reported Interoceptive Accuracy and Attention With Psychopathology. Z Für Klin Psychol Psychother. 2022;51(3–4):165–75.

42. Brettschneider C, Konig HH, Herzog W, Kaufmann C, Schaefert R, Konnopka A. Validity and responsiveness of the EQ-5D in assessing and valuing health status in patients with somatoform disorders. Health Qual Life Outcomes. 20130110th ed. 2013 Jan 10;11:3.

43. Brown RJ, Skehan D, Chapman A, Perry EP, McKenzie KJ, Lloyd DM, et al. Physical symptom reporting is associated with a tendency to experience somatosensory distortion. Psychosom Med. 20120628th ed. 2012 Jul;74(6):648–55.

44. Brunahl CA, Klotz SGR, Dybowski C, Albrecht R, Hoink J, Fisch M, et al. Physiotherapy and combined cognitive-behavioural therapy for patients with chronic pelvic pain syndrome: results of a non-randomised controlled feasibility trial. BMJ Open. 20211214th ed. 2021 Dec 14;11(12):e053421.

45. Cano-Garcia FJ, Munoz-Navarro R, Sese Abad A, Moretti LS, Medrano LA, Ruiz-Rodriguez P, et al. Latent structure and factor invariance of somatic symptoms in the patient health questionnaire (PHQ-15). J Affect Disord. 20190930th ed. 2020 Jan 15;261:21–9.

46. Cao J, Wei J, Fritzsche K, Toussaint AC, Li T, Jiang Y, et al. Prevalence of DSM-5 somatic symptom disorder in Chinese outpatients from general hospital care. Gen Hosp Psychiatry. 20191129th ed. 2020 Jan;62:63–71.

47. Chaabouni A, Houwen J, Grewer G, Liebau M, Akkermans R, van Boven K, et al. The burden of persistent symptom diagnoses in primary care patients: a cross-sectional study. Scand J Prim Health Care. 20240207th ed. 2024 Mar;42(1):112–22.

48. Chalder T, Patel M, Hotopf M, Moss-Morris R, Ashworth M, Watts K, et al. Efficacy of therapist-delivered transdiagnostic CBT for patients with persistent physical symptoms in secondary care: a randomised controlled trial. Psychol Med. 2021;1–11.

49. Chen Y, Wu Y, Mu J, Qiu B, Wang K, Tian Y. Abnormal fear circuits activities correlated to physical symptoms in somatic anxiety patients. J Affect Disord. 20200521st ed. 2020 Sep 1;274:54–8.

50. Cho KJ, Lee NS, Lee YS, Jeong WJ, Suh HJ, Kim JC, et al. The Changes of Psychometric Profiles after Medical Treatment of Lower Urinary Tract Symptoms Suggestive of Benign Prostatic Hyperplasia. Clin Psychopharmacol Neurosci. 2015 Dec 31;13(3):269–74.

51. Choi HJ, Yang CM, Lee SY, Lee HJ, Jang SH. Mental Health and Quality of Life for Healthcare Workers in a University Hospital Under COVID-19. Psychiatry Investig. 20211220th ed. 2022 Feb;19(2):85–91.

52. Ciaramella A, Pozzolini V, Scatena E, Carli G. Can interoceptive sensitivity provide information on the difference in the perceptual mechanisms of recurrent and chronic pain? Part I. A retrospective clinical study related to multidimensional pain assessment. Scand J Pain. 20220923rd ed. 2023 Apr 25;23(2):308–17.

53. Clarke DM, Piterman L, Byrne CJ, Austin DW. Somatic symptoms, hypochondriasis and psychological distress: a study of somatisation in Australian general practice. Med J Aust. 2008 Nov 17;189(10):560–4.

54. Avishai Cohen H, Zerach G. Associations Between Posttraumatic Stress Symptoms, Anxiety Sensitivity, Socially Prescribed Perfectionism, and Severity of Somatic Symptoms Among Individuals with Fibromyalgia. Pain Med. 2021 Feb 23;22(2):363–71.

55. Cooper A, Abbass A, Town J. Implementing a Psychotherapy Service for Medically Unexplained Symptoms in a Primary Care Setting. J Clin Med [Internet]. 20171129th ed. 2017 Nov 29;6(12). Available from: https://www.ncbi.nlm.nih.gov/pubmed/29186054

56. Corpas J, Moriana JA, Vencesla JF, Galvez-Lara M. Brief psychological treatments for emotional disorders in Primary and Specialized Care: A randomized controlled trial. Int J Clin Health Psychol. 20201130th ed. 2021 Jan;21(1):100203.

57. Corpas J, Moriana JA, Vencesla JF, Galvez-Lara M. Effectiveness of brief group transdiagnostic therapy for emotional disorders in primary care: A randomized controlled trial identifying predictors of outcome. Psychother Res. 20210716th ed. 2022 Apr;32(4):456–69.

58. Dadfar M, Kalibatseva Z, Lester D. Reliability and validity of the Farsi version of the Patient Health Questionnaire-9 (PHQ-9) with Iranian psychiatric outpatients. Trends Psychiatry Psychother. 2018;40(2):144–51.

59. Dadfar M, Momeni Safarabad N, Asgharnejad Farid AA, Nemati Shirzy M, Ghazie Pour Abarghouie F. Reliability, validity, and factorial structure of the World Health Organization-5 Well-Being Index (WHO-5) in Iranian psychiatric outpatients. Trends Psychiatry Psychother. 2018;40(2):79–84.

60. Dadfar M, Abdel-Khalek AM, Lester D. The Revised Arabic Scale of Obsession-Compulsion (ASOC): Validation with Iranian psychiatric outpatients. Ment Health Relig Cult. 2020;1–15.

61. Dadfar M, Asgharnejadfarid AA, Hosseini AF, Nasr Esfahani M, Lester D, Kalibatseva Z. Measuring somatic symptoms with the PHQ-15: a comparative study of three Iranian samples. Ment Health Relig Cult. 2020;1–13.

62. Dagnall N, Denovan A, Drinkwater KG. Longitudinal assessment of the temporal stability and predictive validity of the Revised Paranormal Belief Scale. Front Psychol. 20230127th ed. 2022;13:1094701.

63. Moriguchi D, Ishigaki S, Lin X, Kuyama K, Koishi Y, Takaoka R, et al. Clinical identification of the stimulus intensity to measure temporal summation of second pain. Sci Rep. 20220728th ed. 2022 Jul 28;12(1):12915.

64. Denovan A, Dagnall N, Lofthouse G. Neuroticism and Somatic Complaints: Concomitant Effects of Rumination and Worry. Behav Cogn Psychother. 20181107th ed. 2019 Jul;47(4):431–45.

65. Depping MK, Uhlenbusch N, Harter M, Schramm C, Löwe B. Efficacy of a Brief, Peer-Delivered Self-management Intervention for Patients With Rare Chronic Diseases: A Randomized Clinical Trial. JAMA Psychiatry. 2021 Jun 1;78(6):607–15.

© 2024 Hybelius J et al. *JAMA Network Open.*

66. Dinkel A, Marten-Mittag B, Kremsreiter K. Association Between Daily Worry, Pathological Worry, and Fear of Progression in Patients With Cancer. Front Psychol. 20210811th ed. 2021;12:648623.

67. Dischinger MI, Lange L, Vehling S. Loss of resources and demoralization in the chronically ill. Gen Hosp Psychiatry. 20190810th ed. 2019 Nov;61:10–5.

68. Dreher A, Hahn E, Diefenbacher A, Nguyen MH, Boge K, Burian H, et al. Cultural differences in symptom representation for depression and somatization measured by the PHQ between Vietnamese and German psychiatric outpatients. J Psychosom Res. 20170921st ed. 2017 Nov;102:71–7.

69. D'Silva A, Marshall DA, Vallance JK, Nasser Y, Rajagopalan V, Szostakiwskyj JH, et al. Meditation and Yoga for Irritable Bowel Syndrome: A Randomized Clinical Trial. Am J Gastroenterol. 20221011th ed. 2023 Feb 1;118(2):329–37.

70. Eakman AM, Kinney AR, Reinhardt R. Participation, Meaningful Activity, and Social Support Among U.S. Student Service Members/Veterans. OTJR Thorofare N J. 20190311th ed. 2019 Oct;39(4):222–31.

71. Easton SD, Safadi NS, Wang Y, Hasson RG 3rd. The Kessler psychological distress scale: translation and validation of an Arabic version. Health Qual Life Outcomes. 20171027th ed. 2017 Oct 27;15(1):215.

72. Fahrni JO, Cho EY, Engelberger RP, Baumgartner I, von Kanel R. Quality of life in patients with congenital vascular malformations. J Vasc Surg Venous Lymphat Disord. 20131105th ed. 2014 Jan;2(1):46–51.

73. Fairbrass KM, Hamlin PJ, Gracie DJ, Ford AC. Natural history and impact of irritable bowel syndrome-type symptoms in inflammatory bowel disease during 6 years of longitudinal follow-up. Aliment Pharmacol Ther. 20220822nd ed. 2022 Oct;56(8):1264–73.

74. Falahatdoost M, Dolatshahi B, Pourshahbaz A, Dehghani M, Yalguzaghaji MN, Mohammadi Z. Modeling the relationship between attachment styles and somatic symptoms with the mediating role of emotional processing. J Educ Health Promot. 20200630th ed. 2020;9:157.

75. Fallon BA, Basaraba C, Pavlicova M, Ahern DK, Barsky AJ. Differential Treatment Response Between Hypochondriasis With and Without Prominent Somatic Symptoms. Front Psychiatry. 20211108th ed. 2021;12:691703.

76. Fleischer M, Szepanowski F, Tovar M, Herchert K, Dinse H, Schweda A, et al. Post-COVID-19 Syndrome is Rarely Associated with Damage of the Nervous System: Findings from a Prospective Observational Cohort Study in 171 Patients. Neurol Ther. 20220826th ed. 2022 Dec;11(4):1637–57.

77. Flores-Arriaga J, Aso MC, Izagirre A, Sperber AD, Palsson OS, Bangdiwala SI, et al. Prevalence and description of disorders of gut-brain interaction in Spain according to the results of the Rome Foundation Global Epidemiology Study. Neurogastroenterol Motil. 20230403rd ed. 2023 Jun;35(6):e14582.

78. Forney KJ, Burton Murray H, Brown TA, Guadagnoli L, Pucci G, Taft T. Validation of a measure of hypervigilance and anxiety about gastrointestinal symptoms for individuals with elevated eating pathology. Psychol Assess. 20231207th ed. 2024 Jan;36(1):41–52.

79. Fowler JC, Charak R, Elhai JD, Allen JG, Frueh BC, Oldham JM. Construct validity and factor structure of the difficulties in Emotion Regulation Scale among adults with severe mental illness. J Psychiatr Res. 20140810th ed. 2014 Nov;58:175–80.

80. Fowler JC, Lamkin J, Allen JG, Madan A, Oldham JM, Petersen NJ, et al. Personality trait domains predict psychiatric symptom and functional outcomes. Psychother Chic. 20211223rd ed. 2022 Mar;59(1):38–47.

81. Fresán A, González-Castro TB, Pool-García S, Tovilla-Zárate CA, Sanchez de la Cruz JP, López-Narváez ML, et al. Chronic Pain and Depression are Increased in Outpatient Adults with Somatic Symptoms from Secondary Health Care Services. Pain Manag Nurs. 20230306th ed. 2023 Aug;24(4):436–41.

© 2024 Hybelius J et al. *JAMA Network Open.*

82. Freyler A, Simor P, Szemerszky R, Szabolcs Z, Köteles F. Modern health worries in patients with affective disorders. A pilot study. Ideggyogy Sz. 2019 Sep 30;72(9–10):337–41.

83. Fu Y, Lin Q, Xiang Q, Wen X, Liu L. Comparison of SSS-CN and PHQ-15 in the evaluation of patients with suspected psychological disorders in cardiovascular medicine. Front Psychol. 20230302nd ed. 2023;14:1027253.

84. Fujii T, Oka H, Katsuhira J, Tonosu J, Kasahara S, Tanaka S, et al. Association between somatic symptom burden and health-related quality of life in people with chronic low back pain. PLoS One. 20180220th ed. 2018;13(2):e0193208.

85. Ghapanch A, Abasi I, Bitarafan M, Zarabi H, Derakhshan FS, Derakhshan MK, et al. The Psychometric Evaluation of Somatic Symptom Scale-8 in Patients With Major Depressive Disorder. Pract Clin Psychol. 2022;10(1):69–78.

86. Gica S, Kavakli M, Durduran Y, Ak M. The Effect of COVID-19 Pandemic on Psychosomatic Complaints and Investigation of The Mediating Role of Intolerance to Uncertainty, Biological Rhythm Changes and Perceived COVID-19 Threat in this Relationship: A Web-Based Community Survey. Psychiatry Clin Psychopharmacol. 2020;30(2).

87. Gierk B, Kohlmann S, Toussaint A, Wahl I, Brunahl CA, Murray AM, et al. Assessing somatic symptom burden: a psychometric comparison of the patient health questionnaire-15 (PHQ-15) and the somatic symptom scale-8 (SSS-8). J Psychosom Res. 20141114th ed. 2015 Apr;78(4):352–5.

88. Gierk B, Kohlmann S, Hagemann-Goebel M, Löwe B, Nestoriuc Y. Monitoring somatic symptoms in patients with mental disorders: Sensitivity to change and minimal clinically important difference of the Somatic Symptom Scale - 8 (SSS-8). Gen Hosp Psychiatry. 20170718th ed. 2017 Sep;48:51–5.

89. Gitzen H, Schmidt J, Martin A. Subjective and physiological reactivity to emotional stressors in somatic symptom disorder. Int J Psychophysiol. 20231202nd ed. 2024 Jan;195:112273.

90. Goodarzi M, Ahmadi SM, Asle Zaker Lighvan M, Rahmati F, Molavi R, Mohammadi M. Investigating the Psychometric Properties of the 8-Item Somatic Symptom Scale in Non-clinical Sample of Iranian People. Pract Clin Psychol. 2020;57–64.

91. Gould CE, Kok BC, Ma VK, Wetherell JL, Sudheimer K, Beaudreau SA. Video-Delivered Relaxation Intervention Reduces Late-Life Anxiety: A Pilot Randomized Controlled Trial. Am J Geriatr Psychiatry. 20181217th ed. 2019 May;27(5):514–25.

92. Gracie DJ, Williams CJ, Sood R, Mumtaz S, Bholah MH, Hamlin PJ, et al. Poor Correlation Between Clinical Disease Activity and Mucosal Inflammation, and the Role of Psychological Comorbidity, in Inflammatory Bowel Disease. Am J Gastroenterol. 20160322nd ed. 2016 Apr;111(4):541–51.

93. Gray C, Calderbank A, Adewusi J, Hughes R, Reuber M. Symptoms of posttraumatic stress disorder in patients with functional neurological symptom disorder. J Psychosom Res. 20191217th ed. 2020 Feb;129:109907.

94. Grover S, Sahoo S, Chakrabarti S, Avasthi A. Anxiety and somatic symptoms among elderly patients with depression. Asian J Psychiatr. 20180718th ed. 2019 Mar;41:66–72.

95. Grover S, Sahoo S, Naskar C, Sharma A. Psychiatric comorbidities in patients suffering from systemic lupus erythematosus admitted to a tertiary care hospital in Northern India. Lupus. 20230519th ed. 2023 Jul;32(8):1008–18.

96. Habtamu K, Birhane R, Medhin G, Hanlon C, Fekadu A. Psychometric properties of screening questionnaires to detect depression in primary healthcare setting in rural Ethiopia. BMC Prim Care. 20220602nd ed. 2022 Jun 2;23(1):138.

© 2024 Hybelius J et al. *JAMA Network Open.*

97. Hackenberg B, Doge J, O'Brien K, Bohnert A, Lackner KJ, Beutel ME, et al. Tinnitus and Its Relation to Depression, Anxiety, and Stress-A Population-Based Cohort Study. J Clin Med [Internet]. 20230201st ed. 2023 Feb 1;12(3). Available from: https://www.ncbi.nlm.nih.gov/pubmed/36769823

98. Haggarty JM, O'Connor BP, Mozzon JB, Bailey SK. Shared mental healthcare and somatization: changes in patient symptoms and disability. Prim Health Care Res Dev. 20150827th ed. 2016 May;17(3):277–86.

99. Hamdan S, Berkman N, Lavi N, Levy S, Brent D. The Effect of Sudden Death Bereavement on the Risk for Suicide. Crisis. 20191220th ed. 2020 May;41(3):214–24.

100. Han C, Pae CU, Patkar AA, Masand PS, Woong Kim K, Joe SH, et al. Psychometric Properties of the Patient Health Questionnaire–15 (PHQ–15) for Measuring the Somatic Symptoms of Psychiatric Outpatients. Psychosomatics. 2009;50(6):580–5.

101. Hasegawa T, Nishi K, Nakashima A, Moriuchi T, Iso N, Koseki H, et al. Effects of attentional bias modification on chronic low back pain in older outpatients: A randomized crossover trial (pilot study). Med Baltim. 2021 Nov 12;100(45):e27738.

102. Hashimoto K, Takeuchi T, Murasaki M, Hiiragi M, Koyama A, Nakamura Y, et al. Psychosomatic symptoms related to exacerbation of fatigue in patients with medically unexplained symptoms. J Gen Fam Med. 20221011th ed. 2023 Jan;24(1):24–9.

103. Hausteiner C, Bornschein S, Bubel E, Groben S, Lahmann C, Grosber M, et al. Psychobehavioral predictors of somatoform disorders in patients with suspected allergies. Psychosom Med. 20090806th ed. 2009 Nov;71(9):1004–11.

104. Hennemann S, Böhme K, Kleinstäuber M, Baumeister H, Küchler AM, Ebert DD, et al. Internet-based CBT for somatic symptom distress (iSOMA) in emerging adults: A randomized controlled trial. J Consult Clin Psychol. 20220217th ed. 2022 Apr;90(4):353–65.

105. Hennemann S, Killikelly C, Hyland P, Maercker A, Witthöft M. Somatic symptom distress and ICD-11 prolonged grief in a large intercultural sample. Eur J Psychotraumatol. 20230928th ed. 2023;14(2):2254584.

106. Herzog A, Voigt K, Meyer B, Wollburg E, Weinmann N, Langs G, et al. Psychological and interactional characteristics of patients with somatoform disorders: Validation of the Somatic Symptoms Experiences Questionnaire (SSEQ) in a clinical psychosomatic population. J Psychosom Res. 20150312th ed. 2015 Jun;78(6):553–62.

107. Hietaharju M, Kivimaki I, Heikkila H, Napankangas R, Teerijoki-Oksa T, Tanner J, et al. Comparison of Axis II psychosocial assessment methods of RDC/TMD and DC/TMD as part of DC/TMD-FIN phase II validation studies in tertiary care Finnish TMD pain patients. J Oral Rehabil. 20210927th ed. 2021 Dec;48(12):1295–306.

108. Hijne K, Gerritsen L, Pinto AM, da Silva JAP, van Eck van der Sluijs JF, Geenen R. A taxonomy of threat and soothing influences in rheumatic and musculoskeletal diseases and central sensitivity syndromes. Int J Clin Health Psychol. 20231123rd ed. 2024 Jan;24(1):100420.

109. Hilbert A, Brähler E, Schmidt R, Löwe B, Häuser W, Zenger M. Self-Compassion as a Resource in the Self-Stigma Process of Overweight and Obese Individuals. Obes Facts. 20151001st ed. 2015;8(5):293–301.

110. Hinz A, Esser P, Friedrich M, Glaesmer H, Mehnert-Theuerkauf A, Schroeter ML, et al. Changes in anxiety in the general population over a six-year period. PLoS One. 20230912th ed. 2023;18(9):e0291206.

111. Horn M, Wathelet M, Amad A, Martignene N, Lathiere T, Khelfaoui K, et al. Persistent physical symptoms after COVID-19 infection and the risk of Somatic Symptom Disorder. J Psychosom Res. 20230125th ed. 2023 Mar;166:111172.

112. Huth D, Brascher AK, Tholl S, Fiess J, Birke G, Herrmann C, et al. Cognitive-behavioral therapy for patients with post-COVID-19 condition (CBT-PCC): a feasibility trial. Psychol Med. 20231016th ed. 2024 Apr;54(6):1122–32.

113. Hwang KS, Choi HJ, Yang CM, Hong J, Lee HJ, Park MC, et al. The Korean Version of Fear of COVID-19 Scale: Psychometric Validation in the Korean Population. Psychiatry Investig. 20210414th ed. 2021 Apr;18(4):332–9.

114. Hybelius J, Gustavsson A, af Winklerfelt Hammarberg S, Toth-Pal E, Johansson R, Ljótsson B, et al. A unified Internet-delivered exposure treatment for undifferentiated somatic symptom disorder: single-group prospective feasibility trial. Pilot Feasibility Stud. 20220719th ed. 2022 Jul 19;8(1):149.

115. Hyphantis T, Kroenke K, Papatheodorou E, Paika V, Theocharopoulos N, Ninou A, et al. Validity of the Greek version of the PHQ 15-item Somatic Symptom Severity Scale in patients with chronic medical conditions and correlations with emergency department use and illness perceptions. Compr Psychiatry. 20140815th ed. 2014 Nov;55(8):1950–9.

116. Häuser W, Schmutzer G, Brähler E, Glaesmer H. A cluster within the continuum of biopsychosocial distress can be labeled “fibromyalgia syndrome”--evidence from a representative German population survey. J Rheumatol. 20091116th ed. 2009 Dec;36(12):2806–12.

117. Häuser W, Bialas P, Welsch K, Wolfe F. Construct validity and clinical utility of current research criteria of DSM-5 somatic symptom disorder diagnosis in patients with fibromyalgia syndrome. J Psychosom Res. 20150330th ed. 2015 Jun;78(6):546–52.

118. Häuser W, Brähler E, Ablin J, Wolfe F. Modified 2016 American College of Rheumatology Fibromyalgia Criteria, the Analgesic, Anesthetic, and Addiction Clinical Trial Translations Innovations Opportunities and Networks-American Pain Society Pain Taxonomy, and the Prevalence of Fibromyalgia. Arthritis Care Res Hoboken. 2021 May;73(5):617–25.

119. Inan-Budak M, Zonp Z, Kose AM, Saint-Arnault DM. Psychometric evaluation of the Social Support Questionnaire for Transactions in Turkish gender-based violence sample. Arch Psychiatr Nurs. 20230622nd ed. 2023 Aug;45:184–91.

120. Interian A, Allen LA, Gara MA, Escobar JI, Díaz-Martínez AM. Somatic complaints in primary care: further examining the validity of the Patient Health Questionnaire (PHQ-15). Psychosomatics. 2006 Sep;47(5):392–8.

121. Jasper F, Hiller W, Rist F, Bailer J, Witthöft M. Somatic symptom reporting has a dimensional latent structure: results from taxometric analyses. J Abnorm Psychol. 20120528th ed. 2012 Aug;121(3):725–38.

122. Jehangir A, Parkman HP. Reflux Symptoms in Gastroparesis: Correlation With Gastroparesis Symptoms, Gastric Emptying, and Esophageal Function Testing. J Clin Gastroenterol. 2020 May;54(5):428–38.

123. Jeon SW, Han C, Ko YH, Yoon SY, Pae CU, Choi J, et al. Measurement-based Treatment of Residual Symptoms Using Clinically Useful Depression Outcome Scale: Korean Validation Study. Clin Psychopharmacol Neurosci. 2017 Feb 28;15(1):28–34.

124. Johnson KA, Gordon CJ, Grunstein RR. Somatic symptoms are associated with Insomnia disorder but not Obstructive Sleep Apnoea or Hypersomnolence in traumatic brain injury. NeuroRehabilitation. 2019;45(3):409–18.

125. Johnson AJ, Laffitte Nodarse C, Peraza JA, Valdes-Hernandez PA, Montesino-Goicolea S, Huo Z, et al. Psychological profiles in adults with knee OA-related pain: a replication study. Ther Adv Musculoskelet Dis. 20211208th ed. 2021;13:1759720X211059614.

126. Jongsma K, Darboh BS, Davis S, MacKillop E. A cognitive behavioural group treatment for somatic symptom disorder: a pilot study. BMC Psychiatry. 20231130th ed. 2023 Nov 30;23(1):896.

© 2024 Hybelius J et al. *JAMA Network Open.*

127. Jungmann SM, Gropalis M, Schenkel SK, Witthöft M. Is cyberchondria specific to hypochondriasis? J Anxiety Disord. 20231205th ed. 2024 Mar;102:102798.

128. Kalibatseva Z, Leong FTL. Cultural Factors, Depressive and Somatic Symptoms Among Chinese American and European American College Students. J Cross-Cult Psychol. 2018;49(10):1556–72.

129. Kalkbrenner MT, Gainza Perez MA, Hubbard JS. Measurement Invariance of Scores on the Somatic Symptom Scale-8: National Sample of Non-Clinical Adults in the United States. Meas Eval Couns Dev. 20230912th ed. 2024;57(3):263–75.

130. Kamata K, Honda H, Tokuda Y, Takamatsu A, Taniguchi K, Shibuya K, et al. Post-COVID health-related quality of life and somatic symptoms: A national survey in Japan. Am J Med Sci. 20230428th ed. 2023 Aug;366(2):114–23.

131. Kamimura A, Christensen N, Prevedel JA, Tabler J, Hamilton BJ, Ashby J, et al. Quality of life among free clinic patients associated with somatic symptoms, depression, and perceived neighborhood environment. J Community Health. 2014 Jun;39(3):524–30.

132. Kamimura A, Panahi S, Rathi N, Weaver S, Pye M, Sin K, et al. Risks of opioid abuse among uninsured primary care patients utilizing a free clinic. J Ethn Subst Abuse. 20180724th ed. 2020 Jan;19(1):58–69.

133. Kane JS, Irvine AJ, Derwa Y, Rotimi O, Ford AC. High prevalence of irritable bowel syndrome-type symptoms in microscopic colitis: implications for treatment. Ther Adv Gastroenterol. 20180621st ed. 2018;11:1756284818783600.

134. Kara N, Yao AC, Newton J, Deary V, O'Hara J, Wilson JA. General illness and psychological factors in patients with chronic nasal symptoms. Clin Otolaryngol. 20171213th ed. 2018 Apr;43(2):609–16.

135. Kawasaki K, Sugawara S, Watanabe K, Hong C, Tu TTH, Watanabe T, et al. Differences in the Clinical Characteristics of Persistent Idiopathic Facial Pain (Atypical Odontalgia) Patients with or Without Neurovascular Compression of the Trigeminal Nerve. Pain Med. 2020 Apr 1;21(4):814–21.

136. Kealy D, Rice SM, Ogrodniczuk JS, Cox DW. Investigating the Link Between Pathological Narcissism and Somatization. J Nerv Ment Dis. 2018 Dec;206(12):964–7.

137. Khaustova OO, Markova MV, Driuchenko MO, Burdeinyi AO. Proactive psychological and psychiatric support of patients with chronic non-communicable diseases in a randomised trial: a Ukrainian experience. Gen Psychiatr. 20221125th ed. 2022;35(5):e100881.

138. Kim NY, Yoo SK, Jin JC, Yoon YJ, Han DH, Kim SM. Latent Profile Analysis for Classification of Psychosomatic Symptoms in Perimenopausal Women. J Acad Consult Liaison Psychiatry. 20220827th ed. 2023 Mar;64(2):136–46.

139. Kliem S, Beller J, Kroger C, Birowicz T, Zenger M, Brähler E. Dimensional latent structure of somatic symptom reporting in two representative population studies: results from taxometric analyses. Psychol Assess. 20140203rd ed. 2014 Jun;26(2):484–92.

140. Klotz SGR, Ketels G, Löwe B, Brunahl CA. Myofascial Findings and Psychopathological Factors in Patients with Chronic Pelvic Pain Syndrome. Pain Med. 2020 Feb 1;21(2):e34–44.

141. Knopp M, Burghardt J, Oppenauer C, Meyer B, Moritz S, Sprung M. Affective and cognitive Theory of Mind in patients with alcohol use disorder: Associations with symptoms of depression, anxiety, and somatization. J Subst Use Addict Treat. 20231121st ed. 2024 Feb;157:209227.

142. Kobel F, Morawa E, Erim Y. Effectiveness of Inpatient Psychotherapy for Patients With and Without Migratory Background: Do They Benefit Equally? Front Psychiatry. 20200611th ed. 2020;11:542.

143. Kocalevent RD, Hinz A, Brähler E. Standardization of a screening instrument (PHQ-15) for somatization syndromes in the general population. BMC Psychiatry. 20130320th ed. 2013 Mar 20;13:91.

© 2024 Hybelius J et al. *JAMA Network Open.*

144. Koh JS, Ko HJ, Wang SM, Cho KJ, Kim JC, Lee SJ, et al. Depression and somatic symptoms may influence on chronic prostatitis/chronic pelvic pain syndrome: a preliminary study. Psychiatry Investig. 20141020th ed. 2014 Oct;11(4):495–8.

145. Korber S, Frieser D, Steinbrecher N, Hiller W. Classification characteristics of the Patient Health Questionnaire-15 for screening somatoform disorders in a primary care setting. J Psychosom Res. 20110321st ed. 2011 Sep;71(3):142–7.

146. Kube T, Riecke J, Heider J, Ballou SK, Glombiewski JA, Rief W, et al. How the integration of normal medical test results can be improved in patients with somatoform disorders-An experimental study. Health Psychol. 20221222nd ed. 2023 Feb;42(2):103–12.

147. Kuby AK, Löwe B, Fabisch AB, Piontek K, Harter M, Konig HH, et al. Predictors of Seeking Psychotherapy in Primary Care Patients with High Somatic Symptom Burden. Behav Med. 20181211th ed. 2019 Jul;45(3):231–9.

148. Köteles F, Szemerszky R, Freyler A, Bardos G. Somatosensory amplification as a possible source of subjective symptoms behind modern health worries. Scand J Psychol. 20101028th ed. 2011 Apr;52(2):174–8.

149. Laferton JAC, Stenzel NM, Rief W, Klaus K, Brähler E, Mewes R. Screening for DSM-5 Somatic Symptom Disorder: Diagnostic Accuracy of Self-Report Measures Within a Population Sample. Psychosom Med. 2017 Nov;79(9):974–81.

150. Lahmann C, Gebhardt M, Sattel H, Dinkel A, Pieh C, Probst T. A Randomized Controlled Trial on Functional Relaxation as an Adjunct to Psychoeducation for Stress. Front Psychol. 20170927th ed. 2017;8:1553.

151. Langhorst J, Koch AK, Kehm C, Oznur O, Engler H, Häuser W. Mild Water-Filtered Infrared-A Whole-Body Hyperthermia Reduces Pain in Patients with Fibromyalgia Syndrome-A Randomized Sham-Controlled Trial. J Clin Med [Internet]. 20230418th ed. 2023 Apr 18;12(8). Available from: https://www.ncbi.nlm.nih.gov/pubmed/37109279

152. Lanzara R, Conti C, Camelio M, Cannizzaro P, Lalli V, Bellomo RG, et al. Alexithymia and Somatization in Chronic Pain Patients: A Sequential Mediation Model. Front Psychol. 20201027th ed. 2020;11:545881.

153. Le TL, Geist R, Hunter J, Maunder RG. Relationship between insecure attachment and physical symptom severity is mediated by sensory sensitivity. Brain Behav. 20200626th ed. 2020 Aug;10(8):e01717.

154. Lee S, Ma YL, Tsang A. Psychometric properties of the Chinese 15-item patient health questionnaire in the general population of Hong Kong. J Psychosom Res. 20110315th ed. 2011 Aug;71(2):69–73.

155. Lee SY, Ryu HS, Choi SC, Jang SH. Psychological Factors Influence the Overlap Syndrome in Functional Gastrointestinal Disorder and Quality of Life among Psychiatric Patients in South Korea. Psychiatry Investig. 20200311th ed. 2020 Mar;17(3):262–7.

156. Lee JH, Lee D, Hyun S, Hong JS, Kim CH, Kim W, et al. Online Mental Health Assessments of COVID-19 Patients in South Korea. Front Psychiatry. 20210706th ed. 2021;12:685445.

157. Leithner K, Assem-Hilger E, Fischer-Kern M, Loeffler-Stastka H, Sam C, Ponocny-Seliger E. Psychiatric morbidity in gynecological and otorhinolaryngological outpatients: a comparative study. Gen Hosp Psychiatry. 20090220th ed. 2009 May;31(3):233–9.

158. Leonhart R, de Vroege L, Zhang L, Liu Y, Dong Z, Schaefert R, et al. Comparison of the Factor Structure of the Patient Health Questionnaire for Somatic Symptoms (PHQ-15) in Germany, the Netherlands, and China. A Transcultural Structural Equation Modeling (SEM) Study. Front Psychiatry. 20180626th ed. 2018;9:240.

159. Lewis H, Adamson J, Atherton K, Bailey D, Birtwistle J, Bosanquet K, et al. CollAborative care and active surveillance for Screen-Positive EldeRs with subthreshold depression (CASPER): a multicentred

randomised controlled trial of clinical effectiveness and cost-effectiveness. Health Technol Assess. 2017 Feb;21(8):1–196.

160. Li Y, Fang M, Niu L, Fan Y, Liu Y, Long Y, et al. Associations among gastroesophageal reflux disease, mental disorders, sleep and chronic temporomandibular disorder: a case-control study. CMAJ. 2019 Aug 19;191(33):E909–15.

161. Li S, Fong DYT, Wong JYH, McPherson B, Lau EYY, Huang L, et al. Noise sensitivity associated with nonrestorative sleep in Chinese adults: a cross-sectional study. BMC Public Health. 20210401st ed. 2021 Apr 1;21(1):643.

162. Li J, Fritzsche K, Glinka M, Pang Y, Song L, Wang Y, et al. Prevalence of DSM-5 somatic symptom disorder in Chinese patients with breast cancer. Psychooncology. 20220404th ed. 2022 Aug;31(8):1302–12.

163. Li Z, Zhang Y, Pang Y, He Y, Song L, Wang Y, et al. The mediating effect of somatic symptom disorder between psychological factors and quality of life among Chinese breast cancer patients. Front Psychiatry. 20230512th ed. 2023;14:1076036.

164. Li L, Zhang Y, Feng S, Cao Y, Li H, Li X, et al. Reliability and validity of the brief psychosomatic symptom scale (BPSS) in patients from general hospitals. Gen Hosp Psychiatry. 20230329th ed. 2023 Jul;83:1–7.

165. Li J, Li R, Li D, Zhang J, Luo X, Zhang Y. Serum BDNF levels and state anxiety are associated with somatic symptoms in patients with panic disorder. Front Psychiatry. 20230718th ed. 2023;14:1168771.

166. Li Y, Jia S, Cao B, Chen L, Shi Z, Zhang H. Network analysis of somatic symptoms in Chinese patients with depressive disorder. Front Public Health. 20230313th ed. 2023;11:1079873.

167. Liao SC, Huang WL, Ma HM, Lee MT, Chen TT, Chen IM, et al. The relation between the patient health questionnaire-15 and DSM somatic diagnoses. BMC Psychiatry. 20161018th ed. 2016 Oct 18;16(1):351.

168. Liao SC, Huang WL. Psychometric Properties of the Chinese Version of the Cognitions About Body and Health Questionnaire. Neuropsychiatr Treat. 20210421st ed. 2021;17:1135–44.

169. Limburg K, Radziej K, Sattel H, Henningsen P, Dieterich M, Probst T, et al. A Randomized Controlled Trial Evaluating Integrative Psychotherapeutic Group Treatment Compared to Self-Help Groups in Functional Vertigo/Dizziness. J Clin Med [Internet]. 20210520th ed. 2021 May 20;10(10). Available from: https://www.ncbi.nlm.nih.gov/pubmed/34065517

170. Lyoo YC, Ju S, Kim E, Kim JE, Lee JH. The patient health questionnaire-15 and its abbreviated version as screening tools for depression in Korean college and graduate students. Compr Psychiatry. 20131123rd ed. 2014 Apr;55(3):743–8.

171. Löwe B, Spitzer RL, Williams JB, Mussell M, Schellberg D, Kroenke K. Depression, anxiety and somatization in primary care: syndrome overlap and functional impairment. Gen Hosp Psychiatry. 2008 May;30(3):191–9.

172. Löwe B, Kroenke K, Spitzer RL, Williams JB, Mussell M, Rose M, et al. Trauma exposure and posttraumatic stress disorder in primary care patients: cross-sectional criterion standard study. J Clin Psychiatry. 20100601st ed. 2011 Mar;72(3):304–12.

173. Ma YJ, Wang DF, Yuan M, Long J, Chen SB, Wu QX, et al. The mediating effect of health anxiety in the relationship between functional somatic symptoms and illness behavior in Chinese inpatients with depression. BMC Psychiatry. 20190827th ed. 2019 Aug 27;19(1):260.

174. Ma S, Yang J, Cheng H, Wang W, Chen G, Bai H, et al. The central symptoms of depression, anxiety, and somatization: a network analysis. Life. 2022;15(1):933–41.

© 2024 Hybelius J et al. *JAMA Network Open.*

175. Ma CH, Chang HY, Lee HC, Yu YF, Tien HS, Lin YH, et al. The psychological and physiological effects of integrated cognitive-behavioral and biofeedback therapy on panic disorder: A randomized controlled trial. J Formos Med Assoc. 20230714th ed. 2023 Dec;122(12):1305–12.

176. Manning K, Mayorga NA, Nizio P, Heggeness LF, Kauffman BY, Garey L, et al. Exploring Fatigue Sensitivity in terms of Mental Health and Fatigue Severity among a Racially and Ethnically Diverse Sample with Severe Fatigue. Fatigue. 20220608th ed. 2022;10(3):136–45.

177. Manuel J, Rudolph L, Beissner F, Neubert TA, Dusch M, Karst M. Traumatic Events, Posttraumatic Stress Disorder, and Central Sensitization in Chronic Pain Patients of a German University Outpatient Pain Clinic. Psychosom Med. 20230221st ed. 2023 May 1;85(4):351–7.

178. Marks EM, Chambers JB, Russell V, Hunter MS. A novel biopsychosocial, cognitive behavioural, stepped care intervention for patients with non-cardiac chest pain. Health Psychol Behav Med. 2016;4(1):15–28.

179. Martela F, Ryan RM. Distinguishing between basic psychological needs and basic wellness enhancers: the case of beneficence as a candidate psychological need. Motiv Emot. 2019;44(1):116–33.

180. Martinez KL, Mauss C, Andrews J, Saboda K, Huynh JM, Sanoja AJ, et al. Subtle differences in autonomic symptoms in people diagnosed with hypermobile Ehlers-Danlos syndrome and hypermobility spectrum disorders. Am J Med Genet A. 20210407th ed. 2021 Jul;185(7):2012–25.

181. Matsudaira K, Oka H, Kawaguchi M, Murakami M, Fukudo S, Hashizume M, et al. Development of a Japanese version of the Somatic Symptom Scale-8: Psychometric validity and internal consistency. Gen Hosp Psychiatry. 20161208th ed. 2017 Mar;45:7–11.

182. Maulina VVR, Yogo M, Ohira H. Somatic Symptoms: Association Among Affective State, Subjective Body Perception, and Spiritual Belief in Japan and Indonesia. Front Psychol. 20220411th ed. 2022;13:851888.

183. McAndrew LM, Phillips LA, Helmer DA, Maestro K, Engel CC, Greenberg LM, et al. High healthcare utilization near the onset of medically unexplained symptoms. J Psychosom Res. 20170503rd ed. 2017 Jul;98:98–105.

184. McWhirter L, Ritchie C, Stone J, Carson A. Identifying functional cognitive disorder: a proposed diagnostic risk model. CNS Spectr. 20210917th ed. 2022 Dec;27(6):754–63.

185. Menzies RE, Sharpe L, Dar-Nimrod I. The relationship between death anxiety and severity of mental illnesses. Br J Clin Psychol. 20190718th ed. 2019 Nov;58(4):452–67.

186. Menzies RE, Sharpe L, Dar-Nimrod I. The effect of mortality salience on bodily scanning behaviors in anxiety-related disorders. J Abnorm Psychol. 20201210th ed. 2021 Feb;130(2):141–51.

187. Mewes R, Rief W, Brähler E, Martin A, Glaesmer H. Lower decision threshold for doctor visits as a predictor of health care use in somatoform disorders and in the general population. Gen Hosp Psychiatry. 2008 Jul;30(4):349–55.

188. Mitra R, Pujam SNK, Jayachandra A, Sharma P. Dyadic Congruence, Dyadic Coping, and Psychopathology: Implications in Dyads for Patients with Acute Coronary Syndrome. J Mar Med Soc. 2023;25(Suppl 1):S47–54.

189. Mohaghegh F, Moghaddasi M, Eslami M, Dadfar M, Lester D. Disability and its association with psychological factors in multiple sclerosis patients. Mult Scler Relat Disord. 20210105th ed. 2021 Apr;49:102733.

190. Moritz S, Klein JP, Berger T, Larøi F, Meyer B. The Voice of Depression: Prevalence and Stability Across Time of Perception-Laden Intrusive Thoughts in Depression. Cogn Ther Res. 2019;43(6):986–94.

191. Mourad G, Jaarsma T, Stromberg A, Svensson E, Johansson P. The associations between psychological distress and healthcare use in patients with non-cardiac chest pain: does a history of cardiac disease matter? BMC Psychiatry. 20180605th ed. 2018 Jun 5;18(1):172.

192. Müller KW, Beutel ME, Wölfling K. Decreased Occupational Functioning and Increased Physical Health Complaints in Treatment Seekers with Internet-Related Disorders: Compared to Patients with Gambling Disorder. Eur Addict Res. 20190715th ed. 2019;25(5):229–37.

193. Mund M, Uhlenbusch N, Rillig F, Weiler-Normann C, Herget T, Kubisch C, et al. Psychological distress of adult patients consulting a center for rare and undiagnosed diseases: a cross-sectional study. Orphanet J Rare Dis. 20230414th ed. 2023 Apr 14;18(1):82.

194. Mutepfa MM, Motsamai TB, Wright TC, Tapera R, Kenosi LI. Anxiety and somatization: prevalence and correlates of mental health in older people (60+ years) in Botswana. Aging Ment Health. 20200924th ed. 2021 Dec;25(12):2320–9.

195. Müller S, Wendt LP, Spitzer C, Masuhr O, Back SN, Zimmermann J. A Critical Evaluation of the Reflective Functioning Questionnaire (RFQ). J Assess. 20211001st ed. 2022 Sep;104(5):613–27.

196. Nacak Y, Morawa E, Erim Y. High Rejection Sensitivity in Patients With Somatoform Pain Disorder. Front Psychiatry. 20210329th ed. 2021;12:602981.

197. Nasstasia Y, Baker AL, Lewin TJ, Halpin SA, Hides L, Kelly BJ, et al. Differential treatment effects of an integrated motivational interviewing and exercise intervention on depressive symptom profiles and associated factors: A randomised controlled cross-over trial among youth with major depression. J Affect Disord. 20190819th ed. 2019 Dec 1;259:413–23.

198. Nehme A, Barakat M, Malaeb D, Obeid S, Hallit S, Haddad G. Association between COVID-19 symptoms, COVID-19 vaccine, and somatization among a sample of the Lebanese adults. Pharm Pr Granada. 20221221st ed. 2023 Jan;21(1):2763.

199. Newby JM, Hobbs MJ, Mahoney AEJ, Wong SK, Andrews G. DSM-5 illness anxiety disorder and somatic symptom disorder: Comorbidity, correlates, and overlap with DSM-IV hypochondriasis. J Psychosom Res. 20170723rd ed. 2017 Oct;101:31–7.

200. Newby JM, Smith J, Uppal S, Mason E, Mahoney AEJ, Andrews G. Internet-based cognitive behavioral therapy versus psychoeducation control for illness anxiety disorder and somatic symptom disorder: A randomized controlled trial. J Consult Clin Psychol. 20171127th ed. 2018 Jan;86(1):89–98.

201. Nickel JC, Gonzalez YM, Wu Y, Liu Y, Liu H, Iwasaki LR. Chronic Pain-Related Jaw Muscle Motor Load and Sensory Processing. J Dent Res. 20220616th ed. 2022 Sep;101(10):1165–71.

202. Nikendei C, Greinacher A, Berkunova A, Junghanss T, Stojkovic M. Psychological burden and resilience factors in patients with Alveolar Echinococcosis - A cross-sectional study. PLoS Negl Trop Dis. 20190107th ed. 2019 Jan;13(1):e0007082.

203. Nishiyama J, Abe T, Imaizumi S, Yamane A, Fukunaga M. Characteristics of outpatients with functional somatic syndromes at a university hospital's general medicine clinic. J Gen Fam Med. 20220405th ed. 2022 Jul;23(4):268–74.

204. Nordin S, Palmquist E, Nordin M. Psychometric evaluation and normative data for a Swedish version of the Patient Health Questionnaire 15-Item Somatic Symptom Severity Scale. Scand J Psychol. 20130107th ed. 2013 Apr;54(2):112–7.

205. North CS, Hong BA, Lai HH, Alpers DH. Assessing somatization in urologic chronic pelvic pain syndrome. BMC Urol. 20191210th ed. 2019 Dec 10;19(1):130.

206. Orme W, Kapoor S, Frueh BC, Allen JG, Fowler JC, Madan A. Attachment Style Mediates the Relationship between Trauma and Somatic Distress among Individuals with Serious Mental Illness. Psychiatry. 2021 Summer;84(2):150–64.

© 2024 Hybelius J et al. *JAMA Network Open.*

207. Ostrc T, Frankovic S, Pirtosek Z, Rener-Sitar K. Headache Because of Problems with Teeth, Mouth, Jaws, or Dentures in Chronic Temporomandibular Disorder Patients: A Case-Control Study. Int J Env Res Public Health [Internet]. 20220305th ed. 2022 Mar 5;19(5). Available from: https://www.ncbi.nlm.nih.gov/pubmed/35270743

208. Paredes-Echeverri S, Guthrie AJ, Perez DL. Toward a possible trauma subtype of functional neurological disorder: Impact on symptom severity and physical health. Front Psychiatry. 20221115th ed. 2022;13:1040911.

209. Park S, Choi SC, Yang CM, Jang SH, Lee SY. The Korean Version of Pandemic Stress Questionnaire: Validation in Korean Population. Clin Psychopharmacol Neurosci. 2023 May 30;21(2):377–85.

210. Parkes C, Bezzina O, Chapman A, Luteran A, Freeston MH, Robinson LJ. Jumping to conclusions in persistent pain using a somatosensory modification of the beads task. J Psychosom Res. 20190828th ed. 2019 Nov;126:109819.

211. Patel M, James K, Moss-Morris R, Ashworth M, Husain M, Hotopf M, et al. BMC family practice integrated GP care for patients with persistent physical symptoms: feasibility cluster randomised trial. BMC Fam Pr. 20201007th ed. 2020 Oct 7;21(1):207.

212. Patel AR, Newman E, Richardson J. A pilot study adapting and validating the Harvard Trauma Questionnaire (HTQ) and PTSD checklist-5 (PCL-5) with Indian women from slums reporting gender-based violence. BMC Womens Health. 20220128th ed. 2022 Jan 28;22(1):22.

213. Perros P, Nagy EV, Papini E, Van Der Feltz-Cornelis CM, Weetman AP, Hay HA, et al. Hypothyroidism and Somatization: Results from E-Mode Patient Self-Assessment of Thyroid Therapy, a Cross-Sectional, International Online Patient Survey. Thyroid. 20230503rd ed. 2023 Aug;33(8):927–39.

214. Persoons P, Luyckx K, Desloovere C, Vandenberghe J, Fischler B. Anxiety and mood disorders in otorhinolaryngology outpatients presenting with dizziness: validation of the self-administered PRIME-MD Patient Health Questionnaire and epidemiology. Gen Hosp Psychiatry. 2003 Sep;25(5):316–23.

215. Petrelis M, Domeyer PR. Translation and validation of the Greek version of the Somatic Symptom Scale-8 (SSS-8) in patients with chronic low back pain. Disabil Rehabil. 20210316th ed. 2022 Aug;44(16):4467–73.

216. Petrik M, Palmer B, Khoruts A, Vaughn B. Psychological Features in the Inflammatory Bowel Disease-Irritable Bowel Syndrome Overlap: Developing a Preliminary Understanding of Cognitive and Behavioral Factors. Crohns Colitis 360. 20210826th ed. 2021 Jul;3(3):otab061.

217. Philipps A, Silbermann A, Morawa E, Stemmler M, Erim Y. Effectiveness of a Multimodal, Day Clinic Group-Based Treatment Program for Trauma-Related Disorders: Differential Therapy Outcome for Complex PTSD vs. Non-Complex Trauma-Related Disorders. Front Psychiatry. 20191107th ed. 2019;10:800.

218. Pick S, Hodsoll J, Stanton B, Eskander A, Stavropoulos I, Samra K, et al. Trial Of Neurostimulation In Conversion Symptoms (TONICS): a feasibility randomised controlled trial of transcranial magnetic stimulation for functional limb weakness. BMJ Open. 20201006th ed. 2020 Oct 6;10(10):e037198.

219. Pick S, Millman LM, Ward E, Short E, Stanton B, Reinders AS, et al. Unravelling the influence of affective stimulation on functional neurological symptoms: a pilot experiment examining potential mechanisms. J Neurol Neurosurg Psychiatry. 20240412th ed. 2024 Apr 12;95(5):461–70.

220. Pollo CF, Meneguin S, Miot HA, de Oliveira C. Translation, cultural adaptation and validation of the Somatic Symptom Scale-8 (SSS-8) for the Brazilian Portuguese language. BMC Prim Care. 20220905th ed. 2022 Sep 5;23(1):222.

221. Rady A, Alamrawy R, Ramadan I, Abd El Raouf M. Does alexithymia, independent of depressive and anxiety disorders, correlate with the severity of somatic manifestations among patients with medically unexplained physical symptoms? J Exp Psychopathol. 2021;12(4).

© 2024 Hybelius J et al. *JAMA Network Open.*

222. Reddy B, Chaturvedi SK, Desai G, Manjunatha N, Guruprasad S. Health-related life events in patients with somatic symptom disorders: A case control study. Int J Soc Psychiatry. 20190410th ed. 2019 Jun;65(4):265–70.

223. Reuber M, Burness C, Howlett S, Brazier J, Grünewald R. Tailored psychotherapy for patients with functional neurological symptoms: a pilot study. J Psychosom Res. 20070801st ed. 2007 Dec;63(6):625–32.

224. Reutimann S, Hubscher N, Steiner J, Voderholzer U, Augsburger M. Assessing validity of the Klenico diagnostic software system in a large psychotherapeutic inpatient sample. Front Digit Health. 20230824th ed. 2023;5:1176130.

225. Rief W, Glaesmer H, Baehr V, Broadbent E, Brähler E, Petrie KJ. The relationship of modern health worries to depression, symptom reporting and quality of life in a general population survey. J Psychosom Res. 20120120th ed. 2012 Apr;72(4):318–20.

226. Roca M, Gili M, Garcia-Campayo J, Armengol S, Bauza N, Garcia-Toro M. Stressful life events severity in patients with first and recurrent depressive episodes. Soc Psychiatry Psychiatr Epidemiol. 20130421st ed. 2013 Dec;48(12):1963–9.

227. Rohricht F, Sattel H, Kuhn C, Lahmann C. Group body psychotherapy for the treatment of somatoform disorder - a partly randomised-controlled feasibility pilot study. BMC Psychiatry. 20190423rd ed. 2019 Apr 23;19(1):120.

228. Rometsch C, Teufel M, Skoda EM, Schweda A, Cosci F, Zipfel S, et al. Depression and anxiety mediate the relationship between illness representations and perceived distress in patients with chronic pain. Sci Rep. 20230919th ed. 2023 Sep 19;13(1):15527.

229. Roohafza H, Saneian P, Feizi A, Khani A, Yavari N, Bagherieh S, et al. What Predict Self-rated Health: A Cross-sectional Study Among Men and Women with Non-cardiac Chest Pain. J Clin Psychol Med Settings. 20221027th ed. 2023 Sep;30(3):618–27.

230. Ros Montalbán S, Comas Vives A, Garcia-Garcia M. Validation of the Spanish version of the PHQ-15 questionnaire for the evaluation of physical symptoms in patients with depression and/or anxiety disorders: DEPRE-SOMA study. Actas Esp Psiquiatr. 2010;38(6):345–57.

231. Rosales R, Dworetzky B, Baslet G. Cognitive-emotion processing in psychogenic nonepileptic seizures. Epilepsy Behav. 20191112th ed. 2020 Jan;102:106639.

232. Sammarra I, Martino I, Caligiuri ME, Giugno A, Fortunato F, Labate A, et al. The impact of one-year COVID-19 containment measures in patients with mesial temporal lobe epilepsy: A longitudinal survey-based study. Epilepsy Behav. 20220131st ed. 2022 Mar;128:108600.

233. Sayuk GS, North CS, Pollio DE, Gott BM, Alpers DH. Episodic Memories Among Irritable Bowel Syndrome (IBS) Patients: An Important Aspect of the IBS Symptom Experience. Front Pain Res Lausanne. 20220616th ed. 2022;3:892313.

234. Scarella TM, Laferton JA, Ahern DK, Fallon BA, Barsky A. The Relationship of Hypochondriasis to Anxiety, Depressive, and Somatoform Disorders. Psychosomatics. 20151023rd ed. 2016 Mar;57(2):200–7.

235. Schaefert R, Honer C, Salm F, Wirsching M, Leonhart R, Yang J, et al. Psychological and behavioral variables associated with the somatic symptom severity of general hospital outpatients in China. Gen Hosp Psychiatry. 20121206th ed. 2013 May;35(3):297–303.

236. Schaeuffele C, Homeyer S, Perea L, Scharf L, Schulz A, Knaevelsrud C, et al. The unified protocol as an internet-based intervention for emotional disorders: Randomized controlled trial. PLoS One. 20220711th ed. 2022;17(7):e0270178.

237. Schlechter P, Hellmann JH, Morina N. Assessing Somatic Symptoms With the Patient Health Questionnaire (PHQ-15) in Syrian Refugees. Assessment. 20220421st ed. 2023 Jun;30(4):1211–25.

238. Schmalbach B, Roenneberg C, Hausteiner-Wiehle C, Henningsen P, Brähler E, Zenger M, et al. Validation of the German version of the Bodily Distress Syndrome 25 checklist in a representative German population sample. J Psychosom Res. 20200303rd ed. 2020 May;132:109991.

239. Schneider A, Horlein E, Wartner E, Schumann I, Henningsen P, Linde K. Unlimited access to health care--impact of psychosomatic co-morbidity on utilisation in German general practices. BMC Fam Pr. 20110618th ed. 2011 Jun 18;12:51.

240. Schneider A, Rosenberger S, Bobardt J, Bungartz-Catak J, Atmann O, Haller B, et al. Self-help guidebook improved quality of life for patients with irritable bowel syndrome. PLoS One. 20170725th ed. 2017;12(7):e0181764.

241. Shahini N, Ghasemzadeh M, Javan M, Salimi Z. Evaluation of the COVID-19 pandemic effect on the development of somatic symptoms in patients with mood disorders: a case-control study. New Microbes New Infect. 20210713th ed. 2021 Sep;43:100917.

242. Shin C, Han C, Linden M, Chae JH, Ko YH, Kim YK, et al. Standardization of the korean version of the posttraumatic embitterment disorder self-rating scale. Psychiatry Investig. 20121112th ed. 2012 Dec;9(4):368–72.

243. Sikharulidze G, Ratiani L, Sordia M, Sikharulidze E, Khutsishvili T, Lejava K, et al. Comorbidity and Association of Posttraumatic Stress, Depression, Anxiety, and Somatic Complaints in COVID-19 Georgian Patients at the Beginning of Pandemic. Curr Psychiatry Res Rev. 2022;18(3):236–47.

244. Singh P, Lee HN, Rangan V, Ballou S, Lembo J, Katon J, et al. Similarities in Clinical and Psychosocial Characteristics of Functional Diarrhea and Irritable Bowel Syndrome With Diarrhea. Clin Gastroenterol Hepatol. 20190820th ed. 2020 Feb;18(2):399-405 e1.

245. Sitnikova K, Leone SS, van Marwijk HWJ, Twisk J, van der Horst HE, van der Wouden JC. Effectiveness of a cognitive behavioural intervention for patients with undifferentiated somatoform disorder: Results from the CIPRUS cluster randomized controlled trial in primary care. J Psychosom Res. 20190624th ed. 2019 Dec;127:109745.

246. Sogutlu A, Levenson JL, McClish DK, Rosef SD, Smith WR. Somatic symptom burden in adults with sickle cell disease predicts pain, depression, anxiety, health care utilization, and quality of life: the PiSCES project. Psychosomatics. 2011 May;52(3):272–9.

247. Sood M, Ganesh R, Mahapatra A, Verma R, Chadda RK. Somatic symptoms in schizophrenia: Association with socio-demographic and clinical characteristics, disability and quality of life. Indian J Psychiatry. 20230712th ed. 2023 Jul;65(7):749–54.

248. Soucy JN, Hadjistavropoulos HD. Treatment acceptability and preferences for managing severe health anxiety: Perceptions of internet-delivered cognitive behaviour therapy among primary care patients. J Behav Ther Exp Psychiatry. 20170220th ed. 2017 Dec;57:14–24.

249. Speck MJ, Witthöft M. Symptoms of Idiopathic Environmental Intolerance associated with chemicals (IEI-C) are positively associated with perceptual anomalies. J Psychosom Res. 20220403rd ed. 2022 Jun;157:110808.

250. Starcevic V, Choi TY, Kim TH, Yoo SK, Bae S, Choi BS, et al. Internet gaming disorder and gaming disorder in the context of seeking and not seeking treatment for video-gaming. J Psychiatr Res. 20200607th ed. 2020 Oct;129:31–9.

251. Stauder A, Witthöft M, Köteles F. Validation of the Hungarian PHQ-15. A latent variable approach. Ideggyogy Sz. 2021 May 30;74(5–6):183–90.

252. Stegenga BT, Kamphuis MH, King M, Nazareth I, Geerlings MI. The natural course and outcome of major depressive disorder in primary care: the PREDICT-NL study. Soc Psychiatry Psychiatr Epidemiol. 20101106th ed. 2012 Jan;47(1):87–95.

253. Stein J, Vohringer M, Wagner B, Stammel N, Nesterko Y, Bottche M, et al. Exposure Versus Cognitive Restructuring Techniques in Brief Internet-Based Cognitive Behavioral Treatment for Arabic-Speaking People With Posttraumatic Stress Disorder: Randomized Clinical Trial. JMIR Ment Health. 20231213th ed. 2023 Dec 13;10:e48689.

254. Sun DL, Liu QH, Li MJ, Yang Y, Zhang R, Li M, et al. Patient health questionnaire-15 (PHQ-15) to distinguish bipolar II disorder from major depressive disorder. Psychiatry Res. 20200517th ed. 2020 Aug;290:113026.

255. Sweetman J, van der Feltz-Cornelis CM, Elfeddali I, de Beurs E. Validation of the work stress screener (WOSS-13) and resilience at work scale (ReWoS-24). J Psychosom Res. 20220709th ed. 2022 Sep;160:110989.

256. Taenzer M, Loffler-Ragg J, Schroll A, Monfort-Lanzas P, Engl S, Weiss G, et al. Urine Metabolite Analysis to Identify Pathomechanisms of Long COVID: A Pilot Study. Int J Tryptophan Res. 20231222nd ed. 2023;16:11786469231220781.

257. Taimeh D, Riordain RN, Fedele S, Leeson R. Validity and internal consistency of four scales in patients with TMD: PHQ8, GAD7, PHQ15 and JFLS20. Oral Dis. 20231005th ed. 2024 May;30(4):2473–84.

258. Tang L, Fritzsche K, Leonhart R, Pang Y, Li J, Song L, et al. Emotional distress and dysfunctional illness perception are associated with low mental and physical quality of life in Chinese breast cancer patients. Health Qual Life Outcomes. 20171201st ed. 2017 Dec 1;15(1):231.

259. Ten Brink AF, Bultitude JH. Predictors of Self-Reported Neglect-like Symptoms and Involuntary Movements in Complex Regional Pain Syndrome Compared to Other Chronic Limb Pain Conditions. Pain Med. 2021 Oct 8;22(10):2337–49.

260. Terluin B, Barends H, van der Horst HE, Dekker J, van der Wouden JC. Head-to-head comparison of somatic symptom scales: The Patient Health Questionnaire (PHQ-15) and the somatization scale of the Four-Dimensional Symptom Questionnaire (4DSQ-S). J Psychosom Res. 20220906th ed. 2022 Nov;162:111031.

261. Thomas S, Wang Y, Cundiff-O'Sullivan R, Massalee R, Colloca L. How negative and positive constructs and comorbid conditions contribute to disability in chronic orofacial pain. Eur J Pain. 20221018th ed. 2023 Jan;27(1):99–110.

262. Thortveit ET, Lorentzen AR, Ljostad U, Mygland A. Somatic symptoms and fatigue in a Norwegian population with high exposure to ticks. Ticks Tick Borne Dis. 20181009th ed. 2019 Jan;10(1):156–61.

263. Tian P, Ma Y, Hu J, Zhou C, Liu X, Chen Q, et al. Clinical and psychobehavioral features of outpatients with somatic symptom disorder in otorhinolaryngology clinics. J Psychosom Res. 20210617th ed. 2021 Sep;148:110550.

264. Tietjen GE, Brandes JL, Digre KB, Baggaley S, Martin V, Recober A, et al. High prevalence of somatic symptoms and depression in women with disabling chronic headache. Neurology. 2007 Jan 9;68(2):134–40.

265. Toussaint A, Murray AM, Voigt K, Herzog A, Gierk B, Kroenke K, et al. Development and Validation of the Somatic Symptom Disorder-B Criteria Scale (SSD-12). Psychosom Med. 2016 Jan;78(1):5–12.

266. Toussaint A, Kroenke K, Baye F, Lourens S. Comparing the Patient Health Questionnaire - 15 and the Somatic Symptom Scale - 8 as measures of somatic symptom burden. J Psychosom Res. 20170802nd ed. 2017 Oct;101:44–50.

267. Toussaint A, Löwe B, Brähler E, Jordan P. The Somatic Symptom Disorder - B Criteria Scale (SSD-12): Factorial structure, validity and population-based norms. J Psychosom Res. 20170330th ed. 2017 Jun;97:9–17.

268. Toussaint A, Husing P, Kohlmann S, Löwe B. Detecting DSM-5 somatic symptom disorder: criterion validity of the Patient Health Questionnaire-15 (PHQ-15) and the Somatic Symptom Scale-8 (SSS-8) in combination with the Somatic Symptom Disorder - B Criteria Scale (SSD-12). Psychol Med. 20190207th ed. 2020 Jan;50(2):324–33.

269. Tucker JS, Huang W, Green HD Jr, Pollard MS. Patterns of Substance Use and Associations with Mental, Physical, and Social Functioning: A Latent Class Analysis of a National Sample of U.S. Adults Ages 30-80. Subst Use Misuse. 20201109th ed. 2021;56(1):131–9.

270. Turp JC, Schmutzer G, Brähler E, Häuser W. Prevalence of self-reported jaw pain in Germany: two cross-sectional surveys of the general German population. Clin Oral Investig. 20151127th ed. 2016 Nov;20(8):1895–901.

271. Uhlenbusch N, Löwe B, Harter M, Schramm C, Weiler-Normann C, Depping MK. Depression and anxiety in patients with different rare chronic diseases: A cross-sectional study. PLoS One. 20190220th ed. 2019;14(2):e0211343.

272. Voigt K, Wollburg E, Weinmann N, Herzog A, Meyer B, Langs G, et al. Predictive validity and clinical utility of DSM-5 Somatic Symptom Disorder--comparison with DSM-IV somatoform disorders and additional criteria for consideration. J Psychosom Res. 20120920th ed. 2012 Nov;73(5):345–50.

273. Walentynowicz M, Witthöft M, Raes F, Van Diest I, Van den Bergh O. Sensory and affective components of symptom perception. J Exp Psychopathol. 2018;9(2).

274. Wang HM, Huang PY, Yang SC, Wu MK, Tai WC, Chen CH, et al. Correlation between Psychosomatic Assessment, Heart Rate Variability, and Refractory GERD: A Prospective Study in Patients with Acid Reflux Esophagitis. Life Basel [Internet]. 20230903rd ed. 2023 Sep 3;13(9). Available from: https://www.ncbi.nlm.nih.gov/pubmed/37763266

275. Watanabe K, Watanabe M, Takao C, Hong C, Liu Z, Suga T, et al. Clinical Characteristics of Predominantly Unilateral Oral Cenesthopathy With and Without Neurovascular Contact. Front Neurol. 20210920th ed. 2021;12:744561.

276. Weigel A, Löwe B, Kohlmann S. Severity of somatic symptoms in outpatients with anorexia and bulimia nervosa. Eur Eat Disord Rev. 20181015th ed. 2019 Mar;27(2):195–204.

277. Weisskirch RS, Guan SSA, Lazarevic V. The Role of Attachment in Language Brokering and Psychological Well-being among College Students. J Fam Issues. 2020;42(1):156–75.

278. Weziak-Bialowolska D, Bialowolski P. Cultural events - does attendance improve health? Evidence from a Polish longitudinal study. BMC Public Health. 20160805th ed. 2016 Aug 5;16:730.

279. Wilder-Smith CH, Materna A, Olesen SS. Blueberries Improve Abdominal Symptoms, Well-Being and Functioning in Patients with Functional Gastrointestinal Disorders. Nutrients [Internet]. 20230520th ed. 2023 May 20;15(10). Available from: https://www.ncbi.nlm.nih.gov/pubmed/37242279

280. Williams B, Ospina JP, Jalilianhasanpour R, Fricchione GL, Perez DL. Fearful Attachment Linked to Childhood Abuse, Alexithymia, and Depression in Motor Functional Neurological Disorders. J Neuropsychiatry Clin Neurosci. 20181031st ed. 2019 Winter;31(1):65–9.

281. Williams ZJ, Cascio CJ, Woynaroski TG. Psychometric validation of a brief self-report measure of misophonia symptoms and functional impairment: The duke-vanderbilt misophonia screening questionnaire. Front Psychol. 20220722nd ed. 2022;13:897901.

282. Wiltink J, Kliem S, Michal M, Subic-Wrana C, Reiner I, Beutel ME, et al. Mini - social phobia inventory (mini-SPIN): psychometric properties and population based norms of the German version. BMC Psychiatry. 20171125th ed. 2017 Nov 25;17(1):377.

283. Winkler A, Jeromin F, Doering BK, Barke A. Problematic smartphone use has detrimental effects on mental health and somatic symptoms in a heterogeneous sample of German adults. Comput Hum Behav. 2020;113.

284. Witthöft M, Hiller W, Loch N, Jasper F. The latent structure of medically unexplained symptoms and its relation to functional somatic syndromes. Int J Behav Med. 2013 Jun;20(2):172–83.

285. Witthöft M, Fischer S, Jasper F, Rist F, Nater UM. Clarifying the latent structure and correlates of somatic symptom distress: A bifactor model approach. Psychol Assess. 20150601st ed. 2016 Jan;28(1):109–15.

286. Witthöft M, Bräscher AK, Jungmann SM, Köteles F. Somatic Symptom Perception and Interoception. Z Für Psychol. 2020;228(2):100–9.

287. Wittmann L, Dimitrijevic A, Ehlers A, Foa EB, Kessler H, Schellong J, et al. Psychometric properties and validity of the German version of the Post-Traumatic Diagnostic Scale for DSM-5 (PDS-5). Eur J Psychotraumatol. 20210924th ed. 2021;12(1):1965339.

288. Wolfe F, Walitt BT, Katz RS, Häuser W. Symptoms, the nature of fibromyalgia, and diagnostic and statistical manual 5 (DSM-5) defined mental illness in patients with rheumatoid arthritis and fibromyalgia. PLoS One. 20140214th ed. 2014;9(2):e88740.

289. Wolfe F, Michaud K, Klooster PMT, Rasker JJ. Looking at fibromyalgia differently - An observational study of the meaning and consequences of fibromyalgia as a dimensional disorder. Semin Arthritis Rheum. 20221201st ed. 2023 Feb;58:152145.

290. Wong JY, Fong DY, Chan KK. Anxiety and insomnia as modifiable risk factors for somatic symptoms in Chinese: a general population-based study. Qual Life Res. 20150410th ed. 2015 Oct;24(10):2493–8.

291. Wunsch E, Krause L, Gevers TJ, Schramm C, Janik MK, Krawczyk M, et al. Confidence in treatment is contributing to quality of life in autoimmune liver diseases. The results of ERN RARE-LIVER online survey. Liver Int. 20221003rd ed. 2023 Feb;43(2):381–92.

292. Yang CM, Hwang KS, Lee SY, Seo JS, Jang SH. Reliability and Validity of the Korean Version of Somatic Symptom Scale-8. Psychiatry Investig. 20200818th ed. 2020 Aug;17(8):814–21.

293. Yang X, Luo J, Wang P, He Y, Wang C, Yang L, et al. Characteristics and economic burden of patients with somatoform disorders in Chinese general hospitals: a multicenter cross-sectional study. Ann Gen Psychiatry. 20230812th ed. 2023 Aug 12;22(1):30.

294. Yap AU, Natu VP. Inter-relationships between pain-related temporomandibular disorders, somatic and psychological symptoms in Asian youths. J Oral Rehabil. 20200617th ed. 2020 Sep;47(9):1077–83.

295. Yap AU, Kim S, Lee BM, Jo JH, Park JW. Correlates of jaw functional limitation, somatization and psychological distress among different temporomandibular disorder diagnostic subtypes. J Oral Rehabil. 20231018th ed. 2024 Feb;51(2):287–95.

296. Yap AU, Dewi NL, Marpaung C. Psychological characteristics of young adults with temporomandibular disorders, somatization and combined conditions: A multidimensional evaluation. J Oral Rehabil. 20230821st ed. 2023 Dec;50(12):1382–92.

297. Yazici Güleç M, Güleç H, Simşek G, Turhan M, Aydin Sünbül E. Psychometric properties of the Turkish version of the Patient Health Questionnaire-Somatic, Anxiety, and Depressive Symptoms. Compr Psychiatry. 20111013th ed. 2012 Jul;53(5):623–9.

298. Yeon PS, Kim IO, Kang SN, Lee NE, Kim GY, Min GM, et al. Effects of Urban Forest Therapy Program on Depression Patients. Int J Env Res Public Health [Internet]. 20221228th ed. 2022 Dec 28;20(1). Available from: https://www.ncbi.nlm.nih.gov/pubmed/36612825

299. Zeng S, Yu Y, Lu S, Zhang S, Su X, Dang G, et al. Neuro-11: a new questionnaire for the assessment of somatic symptom disorder in general hospitals. Gen Psychiatr. 20230830th ed. 2023;36(4):e101082.

© 2024 Hybelius J et al. *JAMA Network Open.*

300. Zhang Y, Fritzsche K, Leonhart R, Zhao X, Zhang L, Wei J, et al. Dysfunctional illness perception and illness behaviour associated with high somatic symptom severity and low quality of life in general hospital outpatients in China. J Psychosom Res. 20140622nd ed. 2014 Sep;77(3):187–95.

301. Zhang L, Fritzsche K, Liu Y, Wang J, Huang M, Wang Y, et al. Validation of the Chinese version of the PHQ-15 in a tertiary hospital. BMC Psychiatry. 20160405th ed. 2016 Apr 5;16:89.

302. Zhang J, Pan Y, Hong J, Guo H, Wang M, Liu X, et al. Differences of medically unexplained symptoms among patients of different ages and sexes in the psychological clinic of a general hospital and the influencing factors of MUS: A cross-sectional study. Front Psychiatry. 20220804th ed. 2022;13:930212.

303. Zhang Y, Baumeister D, Spanidis M, Engel F, Berens S, Gauss A, et al. How symptoms of simple acute infections affect the SSS-8 and SSD-12 as screening instruments for somatic symptom disorder in the primary care setting. Front Psychiatry. 20230417th ed. 2023;14:1114782.

304. Zhu C, Ou L, Geng Q, Zhang M, Ye R, Chen J, et al. Association of somatic symptoms with depression and anxiety in clinical patients of general hospitals in Guangzhou, China. Gen Hosp Psychiatry. 20111015th ed. 2012 Mar;34(2):113–20.

305. Zhou Y, Xu J, Rief W. Are comparisons of mental disorders between Chinese and German students possible? An examination of measurement invariance for the PHQ-15, PHQ-9 and GAD-7. BMC Psychiatry. 20201001st ed. 2020 Oct 1;20(1):480.

306. Zolotareva AA. Medically Unexplained Symptoms among Adults from Russia: An Assessment using the Patient Health Questionnaire-15. Psychol Russ. 20230615th ed. 2023;16(2):33–47.

307. Zwerenz R, Becker J, Johansson R, Frederick RJ, Andersson G, Beutel ME. Transdiagnostic, Psychodynamic Web-Based Self-Help Intervention Following Inpatient Psychotherapy: Results of a Feasibility Study and Randomized Controlled Trial. JMIR Ment Health. 20171016th ed. 2017 Oct 16;4(4):e41.

308. de Gucht V, Woestenburg DHA, Wilderjans TF. The Different Faces of (High) Sensitivity, Toward a More Comprehensive Measurement Instrument. Development and Validation of the Sensory Processing Sensitivity Questionnaire (SPSQ). J Assess. 20220217th ed. 2022 Nov;104(6):784–99.

309. de Vroege L, Hoedeman R, Nuyen J, Sijtsma K, van der Feltz-Cornelis CM. Validation of the PHQ-15 for somatoform disorder in the occupational health care setting. J Occup Rehabil. 2012 Mar;22(1):51–8.

310. van Niekerk L, Johnstone L, Matthewson M. Predictors of self-compassion in endometriosis: the role of psychological health and endometriosis symptom burden. Hum Reprod. 2022 Jan 28;37(2):264–73.

311. van Niekerk LM, Bromfield H, Matthewson M. Physical and psychological correlates of self and body compassion in women with polycystic ovary syndrome. J Health Psychol. 20211206th ed. 2022 Sep;27(11):2566–80.

312. van Niekerk LM, Muscella G, Quinn M. A validation of the body compassion scale in females. J Health Psychol. 20230316th ed. 2023 Sep;28(10):900–12.

313. van Ravesteijn H, Lucassen P, Bor H, van Weel C, Speckens A. Mindfulness-based cognitive therapy for patients with medically unexplained symptoms: a randomized controlled trial. Psychother Psychosom. 20130809th ed. 2013;82(5):299–310.

314. van Selms MKA, Reda B, Visscher CM, Manfredini D, Lobbezoo F. The effect of singing on pain and psychological well-being in a patient population with pain-related temporomandibular disorders. J Oral Rehabil. 20220624th ed. 2022 Sep;49(9):841–8.

315. van Wouwe NC, Mohanty D, Lingaiah A, Wylie SA, LaFaver K. Impaired Action Control in Patients With Functional Movement Disorders. J Neuropsychiatry Clin Neurosci. 20191007th ed. 2020 Winter;32(1):73–8.

© 2024 Hybelius J et al. *JAMA Network Open.*

316. van der Feltz-Cornelis CM, Allen SF, van Eck van der Sluijs JF. Childhood sexual abuse predicts treatment outcome in conversion disorder/functional neurological disorder. An observational longitudinal study. Brain Behav. 20200207th ed. 2020 Mar;10(3):e01558.

317. van der Feltz-Cornelis CM, Sweetman J, van Eck van der Sluijs JF, Kamp CAD, de Vroege L, de Beurs E. Diagnostic accuracy of the Dutch version of the Somatic Symptom Disorder - B Criteria Scale (SSD-12) compared to the Whiteley Index (WI) and PHQ-15 in a clinical population. J Psychosom Res. 20230812th ed. 2023 Oct;173:111460.

318. Kroenke K, Spitzer RL, Williams JBW. The PHQ-15: Validity of a New Measure for Evaluating the Severity of Somatic Symptoms. Psychosom Med. 2002 Apr;64(2):258–66.

319. Whiting PF, Rutjes AWS, Westwood ME, Mallett S, Deeks JJ, Reitsma JB, et al. QUADAS-2: A Revised Tool for the Quality Assessment of Diagnostic Accuracy Studies. Ann Intern Med. 2011 Oct 18;155(8):529–36.

320. Cao J, Wei J, Fritzsche K, Toussaint AC, Li T, Zhang L, et al. Detecting DSM-5 somatic symptom disorder in general hospitals in China: B-criteria instrument has better accuracy—A secondary analysis. Front Psychiatry [Internet]. 2022 Oct 20 [cited 2024 Apr 2];13. Available from: https://www.frontiersin.org/journals/psychiatry/articles/10.3389/fpsyt.2022.935597/full

321. Gierk B, Kohlmann S, Kroenke K, Spangenberg L, Zenger M, Brähler E, et al. The Somatic Symptom Scale–8 (SSS-8): A Brief Measure of Somatic Symptom Burden. JAMA Intern Med. 2014 Mar 1;174(3):399–407.

322. Nehme A, Moussa S, Fekih-Romdhane F, Yakın E, Hallit S, Obeid S, et al. Expressive suppression moderates the relationship between PTSD from COVID-19 and somatization and validation of the Arabic version of Patient Health Questionnaire-15 (PHQ-15). PLOS ONE. 2024 Jan 25;19(1):e0293081.

323. Li T, Wei J, Fritzsche K, Toussaint AC, Zhang L, Zhang Y, et al. Validation of the Chinese version of the Somatic Symptom Scale-8 in patients from tertiary hospitals in China. Front Psychiatry. 2022;13:940206.

© 2024 Hybelius J et al. *JAMA Network Open.*
