# Supplementary material for: Measurement Properties of the Patient Health Questionnaire–15 and Somatic Symptom Scale–8: A Systematic Review and Meta-Analysis
Source: JAMA Netw Open. 2024 Nov 20;7(11):e2446603. doi: 10.1001/jamanetworkopen.2024.46603 (PMC11579800; doi:10.1001/jamanetworkopen.2024.46603)
Supplement: Supplement 2. — Data Sharing Statement [file jamanetwopen-e2446603-s002.pdf]

Data Sharing Statement

Hybelius. Measurement Properties of the Patient Health Questionnaire–15 and Somatic Symptom Scale–8. *JAMA Netw Open*. Published November 20, 2024.
doi:10.1001/jamanetworkopen.2024.46603

Data
Data available: Yes
Data types: Other (please specify)
Additional Information: Tabulated study-level data will be made available upon reasonable request.
How to access data: jonna.hybelius@ki.se
When available: With publication

Supporting Documents
Document types: Other (please specify)
Additional Information: For pre-specified hypotheses and targets, including the statistical analysis plan and full search terms, please refer to the supplementary material and the pre-registration.
How to access documents: Supplementary material.
When available: With publication

Additional Information
Who can access the data: Anyone requesting the data, given reasonable request.
Types of analyses: Any purpose.
Mechanisms of data availability: Aggregate data, via corresponding author.
